# Supplementary material for: Genetic demultiplexing of pooled single-cell RNA-sequencing samples in cancer facilitates effective experimental design
Source: Gigascience. 2021 Sep 22;10(9):giab062. doi: 10.1093/gigascience/giab062 (PMC8458035; doi:10.1093/gigascience/giab062)
Supplement: giab062_GIGA-D-21-00074_Original_Submission [file giab062_giga-d-21-00074_original_submission.pdf]

GigaScience

Genetic demultiplexing of pooled single-cell RNA-sequencing samples in cancer facilitates effective experimental design

--Manuscript Draft--

**Manuscript Number:**	GIGA-D-21-00074
**Full Title:**	Genetic demultiplexing of pooled single-cell RNA-sequencing samples in cancer facilitates effective experimental design
**Article Type:**	Research
**Funding Information:**	Foundation for the National Institutes of Health (R01CA237170) — Dr Casey S Greene<br>Foundation for the National Institutes of Health (P30 CA042014) — Dr Jennifer Anne Doherty
**Abstract:**	Abstract<br><br>Background: Pooling cells from multiple biological samples prior to library preparation within the same single-cell RNA sequencing experiment provides several advantages, including lower library preparation costs and reduced unwanted technological variation, such as batch effects. Computational demultiplexing tools based on natural genetic variation between individuals provide a simple approach to demultiplex samples, which does not require complex additional experimental procedures. However, these tools have not been evaluated in cancer, where somatic variants, which could differ between cells from the same sample, may obscure the signal in natural genetic variation.<br>Results: Here, we performed in silico benchmark evaluations by combining raw sequencing reads from multiple single-cell samples in high-grade serous ovarian cancer, which has a high copy number burden, and lung adenocarcinoma, which has a high tumor mutational burden, confirming that genetic demultiplexing tools can be effectively deployed on cancer tissue using a pooled experimental design.<br>Conclusions: This strategy provides significant cost savings through pooled library preparation. To facilitate similar analyses at the experimental design phase, we provide freely accessible code and a reproducible Snakemake workflow built around the best-performing tools found in our in silico benchmark evaluations, available at https://github.com/lmweber/snp-dmx-cancer .
**Corresponding Author:**	Stephanie Hicks<br>Johns Hopkins University Bloomberg School of Public Health<br>Baltimor, UNITED STATES
**Corresponding Author Secondary Information:**	
**Corresponding Author's Institution:**	Johns Hopkins University Bloomberg School of Public Health
**Corresponding Author's Secondary Institution:**	
**First Author:**	Lukas M Weber
**First Author Secondary Information:**	
**Order of Authors:**	Lukas M Weber
	Ariel A Hippen
	Peter F Hickey
	Kristofer C Berrett
	Jason Gertz
	Jennifer Anne Doherty
	Casey S Greene

Powered by Editorial Manager® and ProduXion Manager® from Aries Systems Corporation

	Stephanie C Hicks
**Order of Authors Secondary Information:**	
**Additional Information:**	
**Question**	**Response**
Are you submitting this manuscript to a special series or article collection?	No
**Experimental design and statistics**<br><br>Full details of the experimental design and statistical methods used should be given in the Methods section, as detailed in our Minimum Standards Reporting Checklist. Information essential to interpreting the data presented should be made available in the figure legends.<br><br>Have you included all the information requested in your manuscript?	Yes
**Resources**<br><br>A description of all resources used, including antibodies, cell lines, animals and software tools, with enough information to allow them to be uniquely identified, should be included in the Methods section. Authors are strongly encouraged to cite Research Resource Identifiers (RRIDs) for antibodies, model organisms and tools, where possible.<br><br>Have you included the information requested as detailed in our Minimum Standards Reporting Checklist?	Yes
**Availability of data and materials**<br><br>All datasets and code on which the conclusions of the paper rely must be either included in your submission or deposited in publicly available repositories (where available and ethically appropriate), referencing such data using a unique identifier in the references and in	Yes

the “Availability of Data and Materials” section of your manuscript.<br><br>Have you have met the above requirement as detailed in our Minimum Standards Reporting Checklist?	

Manuscript
Click here to access/download;Manuscript;Weber2021_demultiplexing_paper

Genetic demultiplexing of pooled single-cell RNA-sequencing samples in cancer facilitates effective experimental design

Lukas M. Weber[1], Ariel A. Hippen[2], Peter F. Hickey[3], Kristofer C. Berrett[4], Jason Gertz[4], Jennifer Anne Doherty[4], Casey S. Greene[5], Stephanie C. Hicks[1]*

[1] Department of Biostatistics, Johns Hopkins Bloomberg School of Public Health, Baltimore, MD, USA
[2] Department of Systems Pharmacology and Translational Therapeutics, Perelman School of Medicine, University of Pennsylvania, PA, USA
[3] Advanced Technology & Biology Division, Walter and Eliza Hall Institute of Medical Research, Melbourne, Australia
[4] Huntsman Cancer Institute and Department of Population Health Sciences, University of Utah, UT, USA
[5] Department of Biochemistry and Molecular Genetics, University of Colorado School of Medicine, CO, USA

* Corresponding author

Short title: Genetic demultiplexing of scRNA-seq in cancer

Keywords: genetic demultiplexing, single-cell RNA sequencing, cancer, high-grade serous ovarian cancer, lung adenocarcinoma, tumor mutational burden, computational methods, simulations, benchmarking

Date: 3 March 2021

Abstract

Background: Pooling cells from multiple biological samples prior to library preparation within the same single-cell RNA sequencing experiment provides several advantages, including lower library preparation costs and reduced unwanted technological variation, such as batch effects. Computational demultiplexing tools based on natural genetic variation between individuals provide a simple approach to demultiplex samples, which does not require complex additional experimental procedures. However, these tools have not been evaluated in cancer, where somatic variants, which could differ between cells from the same sample, may obscure the signal in natural genetic variation.

Results: Here, we performed *in silico* benchmark evaluations by combining raw sequencing reads from multiple single-cell samples in high-grade serous ovarian cancer, which has a high copy number burden, and lung adenocarcinoma, which has a high tumor mutational burden, confirming that genetic demultiplexing tools can be effectively deployed on cancer tissue using a pooled experimental design.

Conclusions: This strategy provides significant cost savings through pooled library preparation. To facilitate similar analyses at the experimental design phase, we provide freely accessible code and a reproducible Snakemake workflow built around the best-performing tools found in our *in silico* benchmark evaluations, available at https://github.com/lmweber/snp-dmx-cancer.

Background

Sample pooling prior to library preparation is an effective strategy for experimental design in single-cell RNA sequencing (scRNA-seq) studies, which allows researchers to assess and address unwanted technological variation such as batch effects [1,2] and reduces library preparation costs [3–5]. Several strategies involve pooling cells, labeled or otherwise identifiable in some way, from multiple biological samples, followed by combined library preparation and sequencing, and computational demultiplexing to recover the sample identities of each cell. While sample pooling creates doublets consisting of cells from multiple individuals, with the doublet rate depending on the concentration of loaded cells [5], demultiplexing approaches can also identify doublets at the demultiplexing step without relying on downstream doublet identification tools [6–10]. Depending on the method used, these techniques can also avoid the phenomenon of sample index swapping, which occurs when individually prepared libraries are subsequently pooled for sequencing [11–15].

Existing demultiplexing approaches differ in their experimental procedures, computational methodology for demultiplexing, and demultiplexing accuracy. In barcoding-based approaches (e.g. MULTI-seq [16] and cell hashing [17], and GMM-Demux for doublet identification [18]), cells are experimentally tagged with universal oligonucleotides or antibodies together with sample-specific labels, which can give highly accurate demultiplexing performance, but these approaches make sample preparation more complex and increase costs due to reagent purchases as well as additional library preparation and sequencing. Alternatively, genetic variation-based approaches rely only on natural genetic variation between samples from different individuals (such as single nucleotide polymorphisms, SNPs), which does not require additional experimental procedures at the single-cell level. Initial genetic variation-based demultiplexing methods, such as demuxlet [5], require a known genotype reference for each sample obtained using SNP arrays, whole exome sequencing, or bulk RNA sequencing. Recently, new methods have been developed, such as Vireo [3], scSplit [4], souporcell [19], and freemuxlet [20], which can use probabilistic models to infer the genotype directly from the single-cell reads. Depending

on the method, there is also the option to improve performance by providing either sample-specific genotypes, such as from matched bulk RNA sequencing, or a list of population SNPs, such as from the 1000 Genomes Project [21] for human samples.

Recently, genetic variation-based scRNA-seq demultiplexing tools have been applied to pooled samples from cancer cell lines [22,23], using known genotype references [5,22] and pools consisting of up to dozens of cell lines. However, systematic evaluations have not yet been performed in cancer for methods that do not require a genotype reference [3,4], and using pooled samples from the same cancer type from different individuals, which are likely to be harder to distinguish than cell lines from distinct cancer types. Cancer is characterized by widespread additional somatic mutations, including single nucleotide variants (SNVs) [24], as well as structural variation affecting the frequency of SNVs, which could interfere with the SNP signal used to distinguish individuals in this application of demultiplexing. The frequency of additional somatic SNVs, known as the tumor mutational burden (TMB), can vary widely between cancer types [25], as well as between patients and cancer subtypes [26,27]. However, the TMB is typically small relative to the overall population SNP burden [24]. For example, population SNPs with minor allele frequency (MAF) >1% are thought to occur on the order of once per 1000 nucleotides on average, or 1000 SNPs per Mb [28]. By contrast, high-TMB cancers have been defined as having around >10 or >20 additional mutations (SNVs) per Mb [26,27] -- approximately two orders of magnitude lower frequency than the population SNPs. In the case of typical scRNA-seq protocols that sequence the 3' end of transcripts, only SNPs within the sequenced region (e.g. 100-200 nucleotides) can be detected, but the same arguments may be applied to compare the proportion of cancer SNVs against background SNPs. Therefore, it seems reasonable to expect that the natural genetic variation signal would not be severely obscured by the TMB, and that genetic variation-based demultiplexing tools should still perform well for pooled tissue samples from the same cancer type from different individuals. However, this assumption has not been rigorously tested. Due to the finite and irreplaceable nature of tumor samples, we computationally evaluated demultiplexing algorithms to confirm that genetic variation-based demultiplexing performs adequately when applied to scRNA-seq

pooling experimental designs in cancer, before committing samples to this experimental design strategy. In addition, we were interested in evaluating the degree to which these tools can reliably identify doublets consisting of cells from multiple individuals, including in experimental designs with extremely high proportions of doublets. Reliable doublet identification would allow the use of “super-loading” experimental designs, such as loading cells at very high concentration and subsequently removing identifiable doublets, providing substantial cost savings during library preparation [5,17,29]. In the future, these tools may also be well-suited for cell atlas initiatives, which are expected to cover large numbers of samples, including eventually those from cancer [30,31].

Here, we performed a benchmark evaluation of genetic variation-based demultiplexing in cancer scRNA-seq data using *in silico* simulations constructed from experimental scRNA-seq datasets with known sample identity for each cell. We evaluated two demultiplexing algorithms and five strategies for selecting the genotype reference list of SNPs used in the demultiplexing algorithms, including strategies that do not require a matched genotype reference. In addition, we included varying proportions of simulated doublets by combining raw sequencing reads from multiple cell barcodes, which creates both identifiable doublets (from different individuals) and unidentifiable doublets (from the same individual). In the benchmark evaluation, we considered scRNA-seq samples from cancers that should be difficult to characterize: high-grade serous ovarian cancer (HGSOC) and lung adenocarcinoma. HGSOC is characterized by loss of TP53, and generally has medium to high SNV burden and high copy number variation (CNV) burden (particularly for focal copy number alterations), relative to other cancers [25,32], while lung adenocarcinoma is characterized by high SNV burden [25]. Our results demonstrate that genetic variation-based demultiplexing provides high recall at acceptable precision-recall tradeoffs in both high CNV and high SNV cancer types, even with extremely high simulated doublet proportions. Our results demonstrate that these tools support experimental designs that incorporate sample pooling. We provide a reproducible Snakemake [33] workflow based on the best-performing combination of tools for estimating a genotype reference list of SNPs and demultiplexing samples identified in our benchmark, to facilitate experimental design efforts. The Snakemake workflow is modular, allowing

users to substitute alternative tools. The workflow requires a set of scRNA-seq pilot samples, access to a Linux computing cluster, some familiarity with the Linux command line, and optionally matched bulk RNA-seq samples (for the highest demultiplexing performance and doublet identification). All code for the benchmark evaluation and Snakemake workflow is freely accessible at https://github.com/lmweber/snp-dmx-cancer.

Data Description

Genetic demultiplexing in HGSOC and lung adenocarcinoma

We evaluated the performance of genetic demultiplexing algorithms for scRNA-seq samples from HGSOC (high CNV) and lung adenocarcinoma (high SNV) using a set of benchmark evaluations and Snakemake [33] workflow built around freely available tools including Cell Ranger [34], samtools [35], bcftools [36], Unix string manipulation tools (sed and awk), cellSNP [37], and Vireo [3] (**Methods** and **Figure 1**). The HGSOC samples were collected at the Huntsman Cancer Institute, and the lung adenocarcinoma dataset is a published dataset sourced from [38]. **Table 1** provides a summary of the scRNA-seq datasets. Additional details on data collection and accessibility are provided in **Methods**.

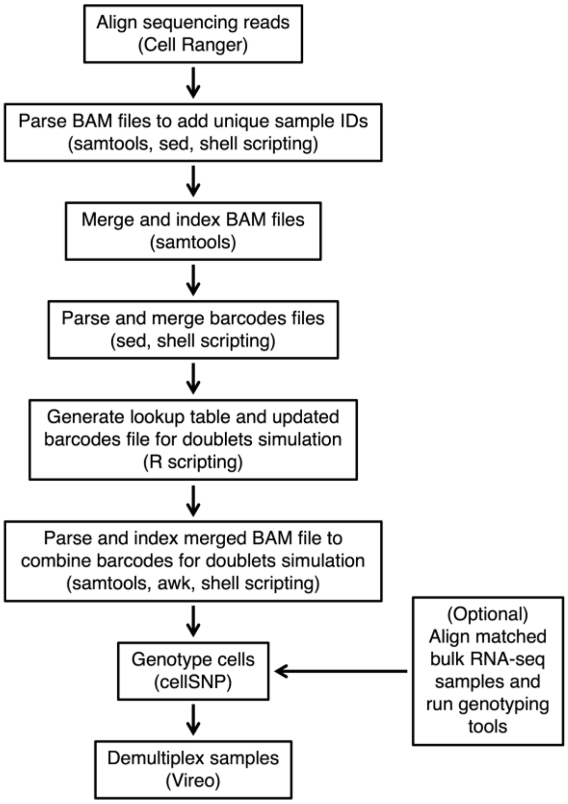

Figure 1. Schematic illustrating the steps in the Snakemake workflow. The workflow is designed to be modular, allowing users to substitute alternative tools. The Snakemake workflow runs a complete analysis for one dataset (HGSOC) and doublets simulation scenario (20% doublets). Our benchmark evaluations include a second dataset (lung adenocarcinoma) and additional doublet simulation scenarios (30% doublets, no doublets). The optional step to run genotyping tools (e.g. on matched bulk RNA-seq samples) improved performance in our benchmark. Tools used in each step are shown in parentheses.

HGSOC dataset		Lung adenocarcinoma dataset	
Sample ID	Number of cells	Sample ID	Number of cells
X2	7123	T08	4093
X3	1533	T09	4267
X4	6546	T20	4521
		T25	4428
		T28	5789
		T31	7069

Table 1. Summary of number of samples and number of cells per sample for scRNA-seq samples in HGSOC (GSE158937 and phs002262.v1.p1) and lung adenocarcinoma [38] (EGAD00001005054) datasets. The numbers of cells per sample listed are the numbers of cells provided by Cell Ranger [34] following sequencing read alignment. The HGSOC dataset additionally includes matched bulk RNA-seq samples for each sample. The lung adenocarcinoma dataset includes matched bulk whole exome sequencing samples for each sample, but not matched bulk RNA-seq samples.

Analyses

High precision and recall performance using genetic demultiplexing

Using the HGSOC scRNA-seq and matched bulk RNA-seq data, we found the highest recall (defined as the proportion of true singlet cells for each sample that are identified as singlets and assigned to the correct sample) and best precision-recall tradeoff (where precision is defined as the proportion of identified cells for each sample that are true singlet cells from the correct sample), when using bcftools [36] to generate a genotype reference list of SNPs from the matched bulk RNA-seq samples, together with cellSNP/Vireo [3,37] for demultiplexing, in all simulation scenarios (30% doublets, 20% doublets, or no doublets) (**Figure 2 a-c**). This scenario (labeled “bulkBcftools_cellSNPVireo” and colored light blue in **Figure 2**) achieves 99.9%, 99.9%, and 99.0% recall (values averaged across three scRNA-seq samples). However, in this scenario, the precision drops (77.4%, 85.9%, and 100%) (values averaged across three scRNA-seq samples) as the percentage of doublets increases with 30%, 20%, and no doublets (**Figure 2 a-c**, panels from left to right), respectively. In general, we prefer higher recall at the expense of somewhat lower precision, so that we do not lose informative singlet cells, and since additional doublet detection tools [6–10] can potentially be applied during downstream analyses to further improve precision.

We also found that using bcftools [36] to generate a genotype reference list of SNPs from the matched bulk RNA-seq samples together with demuxlet (labeled “bulkBcftools_demuxlet” and colored green in **Figure 2**) resulted in somewhat higher precision (84.3%, 91.3%) with a large reduction to recall (52.1%, 53.0%) in the 30% and 20% doublet scenarios, respectively (**Figure 2 a-b**). However, no further improvement in precision was observed (99.9%) with a large reduction in recall (52.8%) for the no doublets scenario (**Figure 2 c**).

In the scenarios where matched bulk RNA-seq samples are not available, the next best-performing scenarios were obtained using the genotype reference from the 1000 Genomes Project [21] (provided

by the authors of cellSNP/Vireo) with no filtering of SNPs ("unfiltered") and genotype reference from the 1000 Genomes Project filtered to retain only SNPs in the 3' untranslated region (UTR) ("filtered"), together with cellSNP/Vireo for demultiplexing (labeled "1000GenomesUnfilt_cellSNPVireo" and "1000GenomesFilt_cellSNPVireo" and colored in orange and purple, respectively) (**Figure 2 a-c**). The "unfiltered" scenario achieved recall 99.0%, 99.0%, and 97.9%, and precision 74.5%, 84.0%, and 100%, with 30% doublets, 20% doublets, and no doublets respectively (**Figure 2 a-c**, panels from left to right). The "filtered" scenario achieved recall 95.3%, 95.0%, and 94.3%, and precision 73.8%, 83.7%, and 100%, respectively. Alternatively, when we evaluated the scenario to call SNPs directly from the scRNA-seq samples and use cellSNP/Vireo for demultiplexing (labeled "singlecellCellSNP_cellSNPVireo" and colored in dark blue), we found comparable recall (92.1%, 91.9%, 91.5%) with a slight loss in precision (72.7%, 82.3%, 99.7%) as the percentage of doublets increases with 30%, 20%, and no doublets, respectively (**Figure 2 a-c**, panels from left to right).

Using the high-TMB lung adenocarcinoma scRNA-seq (without matched bulk RNA-seq) dataset, we only considered the scenario using the genotype reference from the 1000 Genomes Project (filtered) together with cellSNP/Vireo (labeled "1000GenomesFilt_cellSNPVireo" and colored in purple in **Figure 2**), as this resulted in the highest precision and recall in the HGSOC evaluation when using either the genotype reference from 1000 Genomes Project or directly calling SNPs from the scRNA-seq samples, while also keeping runtimes lower than the 1000 Genomes (unfiltered) scenario. In this scenario (labeled "1000GenomesFilt_cellSNPVireo"), we found comparable ranges of precision and recall values as for the matching scenario in the HGSOC dataset (**Figure 2 d-f**). These results demonstrate that we can also achieve excellent demultiplexing performance even in a higher-TMB cancer setting.

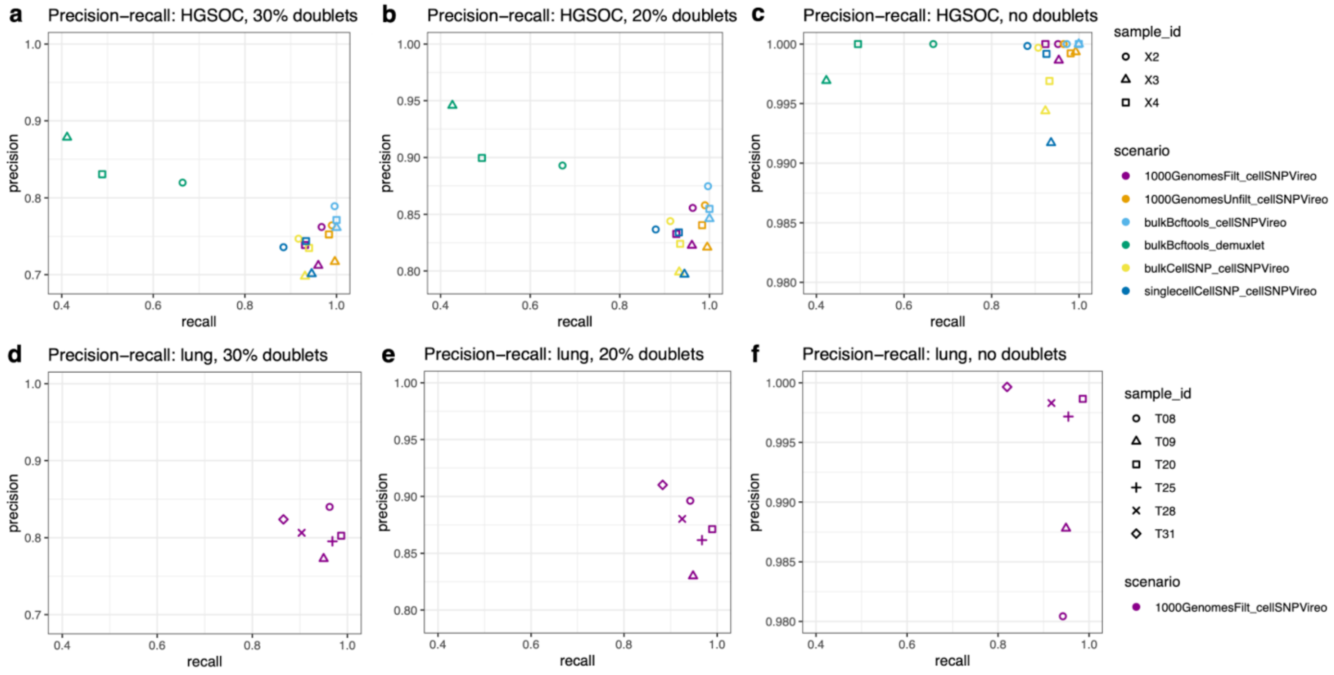

Figure 2. Performance evaluations for benchmark scenarios, for HGSOC dataset **(a-c)** and lung adenocarcinoma dataset **(d-f)**, across three proportions of simulated doublets (30%, 20%, and no doublets). Performance is evaluated in terms of precision (y-axis) and recall (x-axis) for recovering the sample identities of singlet cells for each scRNA-seq sample. Benchmark scenarios are labeled by color and with the naming scheme "genotypeMethod_demultiplexingMethod". Samples within each dataset are identified with shapes. Note that y-axis limits (precision) are the same between rows of panels (datasets), but differ between columns of panels (doublet proportions) for improved visibility.

Computational runtime of genetic demultiplexing workflow steps and genotyping tools

We evaluated the computational runtimes for the various components in our benchmark scenarios and Snakemake workflow using the HGSOC data. First, we found the computational runtimes for the various steps in the genetic demultiplexing workflow vary across multiple orders of magnitude and depended on whether the tool could be parallelized. The parallelizable tools (Cell Ranger and cellSNP) were run using 10 processor cores to decrease runtime, while the remaining tools used a single core. All evaluations of runtimes were performed on a high-performance Linux computing cluster. In the Snakemake workflow (**Figure 3 a**), the slowest steps were running Cell Ranger (approximately 6 hours per sample using 10 cores) and parsing the merged BAM file containing aligned reads to combine cell barcodes into simulated doublets (approximately 1 day). For the cellSNP step in the workflow, runtime depended on the choice of genotype reference list of SNPs (**Figure 3 b**). In particular, filtering the genotype reference from the 1000 Genomes Project [21] (provided by the authors of cellSNP/Vireo) to retain only SNPs in the 3' UTR reduced runtime from approximately 2.5 hours to less than 10 minutes ("1000GenomesUnfilt_cellSNP" vs. "1000GenomesFilt_cellSNP"), at the cost of only a small drop in performance (**Figure 2**). The runtime shown for the cellSNP step in **Figure 3 a** corresponds to the highest-performing scenario from **Figure 2** ("bulkBcftools_cellSNP").

We also evaluated computational runtimes for the genotyping tools used to generate the genotype reference lists of SNPs from either the matched bulk RNA-seq samples or directly from the scRNA-seq samples (**Figure 3 c**). Here, we found by far the slowest option was to use cellSNP to generate the genotype reference directly from the scRNA-seq samples (between 1 and 4.5 days per sample using 10 cores), while generating the genotype reference from the bulk RNA-seq samples took either approximately 2 minutes per sample using cellSNP (10 cores) or 5 hours using bcftools.

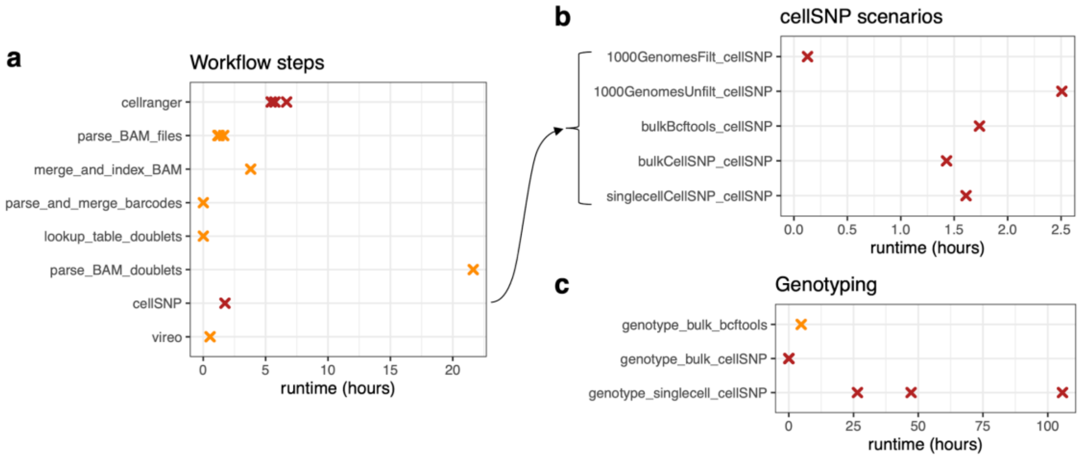

Figure 3. Computational runtimes (in hours) of genetic demultiplexing workflow steps and genotyping tools. **(a)** Runtimes for steps in the complete Snakemake workflow, for a single dataset (HGSOC) and doublets simulation scenario (20%). Parallelized tools (Cell Ranger and cellSNP; points indicated in dark red) were run using 10 processor cores, and all other tools using a single core (points indicated in orange), on a high-performance Linux computing cluster. For steps where samples were processed individually, separate points are shown for each sample. **(b)** Runtimes for alternative options for running cellSNP in the workflow, depending on the choice of genotype reference (1000 Genomes filtered, 1000 Genomes unfiltered, matched bulk RNA-seq using bcftools, matched bulk RNA-seq using cellSNP, and single-cell RNA-seq using cellSNP). The cellSNP step in (a) matches the row “bulkBcftools_cellSNP” in (b), which was the highest-performing scenario from Figure 2. **(c)** Runtimes for alternative options to generate genotype reference file. Note that horizontal axis scales differ between panels for improved visibility.

Discussion

Pooled single-cell experimental designs before library preparation together with genetic variation-based computational sample demultiplexing are a convenient and effective strategy for reducing library preparation costs and potential batch effects in scRNA-seq studies. Here, we performed an *in silico* benchmark evaluation based on real scRNA-seq datasets to confirm that these tools can be effectively applied to pooled cancer samples from different individuals. We selected HGSOC and lung adenocarcinoma, cancer types characterized by a relatively high TMB. Previous benchmark evaluations [3,4] have only evaluated these tools in non-cancer datasets, which are not affected by additional mutational SNV burden that could potentially obscure the natural genetic variation SNP signal used to distinguish individuals, while previous evaluations in cancer [22,23] have relied on matched genotype references and focused on cancer cell lines, which are likely to be easier to distinguish than samples of the same cancer type from different individuals. Our benchmark evaluations include high proportions of simulated doublets (up to 30%), confirming that these tools can be used to identify singlet cells in "super-loading" experimental designs to achieve considerable cost savings in library preparation [5,17,29]. As an illustration of expected cost savings due to lower library preparation costs in a multiplexed experimental design, we estimated library preparation and sequencing costs for designs with 4 to 8 samples, using the "Cost Per Cell" online calculator provided by the Satija Lab [29] (**Supplementary Figure 1**). We assumed 4,000 desired cells per sample after demultiplexing, i.e. after discarding identifiable doublets consisting of cells from multiple samples, but including the smaller number of non-identifiable doublets (multiple cells from the same sample, which have the same SNP profiles and cannot be distinguished using genetic demultiplexing). These designs result in cost savings of approximately 60% of the estimated cost for the experiment when using full multiplexing (all samples prepared as a single library and sequenced together) compared to no multiplexing (**Supplementary Figure 1**).

In our HGSOC dataset, we achieved the best demultiplexing performance (and relatively efficient runtimes) when using matched bulk RNA-seq samples to generate a genotype reference list of SNPs using bcftools [36], together with cellSNP/Vireo [3,37] for demultiplexing. However, using a standard list of population SNPs from the 1000 Genomes Project [21] (which does not require matched bulk RNA-seq samples) provided by the authors of cellSNP/Vireo also achieved good performance. In this case, filtering the population SNPs to retain only SNPs in the 3' UTR significantly reduced runtime, at the cost of only slightly lower demultiplexing performance. For the lung adenocarcinoma dataset, performance was comparable to the matching scenario in the HGSOC dataset, confirming that performance was not seriously affected by the higher TMB, and that genetic demultiplexing can be effectively applied in this setting. Since most other cancer types have lower TMB [25], we expect these results to apply to most cancer types. We provide a freely available, modular Snakemake [33] workflow implementing the best-performing scenario from our benchmark evaluations, built around cellSNP/Vireo [3,37] and other freely accessible tools, as well as additional R and shell scripts to reproduce all analyses in our benchmark evaluation (https://github.com/lmweber/snp-dmx-cancer), to allow other researchers to perform similar analyses for experimental design, planning, and budgeting purposes in their own datasets.

Our study has several limitations. While the best-performing benchmark scenario achieves excellent recall, precision is somewhat lower. This could be addressed using additional downstream tools to identify doublets [6–10]. In general, we prefer higher recall at the expense of somewhat lower precision, so that we are not losing informative cells during the initial steps of the analysis. In this study, we have built our evaluations around the best-performing tools (cellSNP/Vireo [3,37] for demultiplexing and using matched bulk RNA-seq samples for genotyping) and compared against alternative tools, such as demuxlet [5] and baseline scenarios (no doublets), but we have not performed a comprehensive benchmark evaluation of all available tools, such as additional tools for demultiplexing (e.g. scSplit [4], souporcell [19], and freemuxlet [20]). However, we have implemented the Snakemake workflow to be modular, so that other users may substitute alternative tools if they prefer. We also investigated the use of salmon alevin [39] for pseudoalignment of scRNA-seq reads (instead of Cell Ranger), but found that

this was not compatible with the demultiplexing tools since pseudoalignment occurs at the transcriptomic instead of genomic level. However, future developments may enable conversion between transcriptomic and genomic aligned reads, and we have included alternative code scripts for salmon alevin within our benchmark code repository. Our evaluations only considered two tumor types (HGSOC and lung adenocarcinoma), and performance may differ for other cancer types or tissues. However, since we were able to demonstrate good performance in lung adenocarcinoma, one of the highest TMB cancers, we anticipate these results will also be applicable for other cancer types, which will generally have lower TMB. For the lung adenocarcinoma dataset, matched bulk whole exome sequencing data were also available for these six samples, which could be used to further improve performance using additional genotyping tools.

More fundamentally, due to the reliance on genetically distinct SNP profiles, genetic demultiplexing tools are expected to work well for human samples from unrelated individuals, but are not applicable to biological samples from inbred mice or hereditary related human populations, or samples from the same individual [3]. For the same reason, genetic demultiplexing does not allow identifying doublets consisting of cells from the same individual, although these are only a subset of total doublets, and decrease as a proportion of total doublets with increasing number of multiplexed samples. We also have not considered the question of identifying doublets consisting of distinct cell types (from either the same or different individuals), which may be identified using downstream analysis tools. For some experiments, a useful design strategy may also be to combine genetic-based and barcoding-based multiplexing, e.g. multiple treatments on samples from the same individual. Genetic demultiplexing may also fail in the case of experimental problems during library preparation, such as inefficient cell dissociation, or large amounts of ambient RNA from lysed cells or other cell debris. Our Snakemake workflow can be used to demultiplex up to around 12 pooled samples without a genotype reference (limited by the demultiplexing algorithm Vireo) -- beyond this, the demultiplexing performance of the Vireo algorithm has been shown to decrease [3]. For larger experiments, if matched bulk RNA-seq samples are not available, multiple sample pools could be used, with demultiplexing done separately

for each pool [3]. Splitting an experiment across multiple pools and demultiplexing within each pool also represents an opportunity to implement improved experimental designs to reduce batch effects and confounding. Finally, the Snakemake workflow is relatively computationally intensive, and requires access to a high-performance Linux computing cluster or server.

Methods

Benchmark evaluations and workflow

We begin by describing in detail our benchmark evaluation framework, and note that our additional Snakemake [33] workflow is built around the combination of tools that resulted in the best performance from the benchmark evaluation. Specifically, the benchmark and workflow make use of several freely available tools, including Cell Ranger [34], samtools [35], bcftools [36], Unix string manipulation tools (sed and awk), cellSNP [37], and Vireo [3]. The Snakemake workflow is designed to be modular, allowing other alternative or new tools to be substituted. All code for the benchmark evaluation and Snakemake workflow is freely available at https://github.com/lmweber/snp-dmx-cancer.

In our benchmark evaluation, we considered two genetic demultiplexing algorithms: (i) Vireo [3] together with cellSNP [37], and (ii) demuxlet [5] as an alternative genetic-based demultiplexing tool. We evaluated five scenarios for obtaining the genotype reference list of SNPs used in the demultiplexing algorithm: (i) list of population SNPs from the 1000 Genomes Project [21] provided by the authors of cellSNP/Vireo; (ii) list of population SNPs from the 1000 Genomes Project with an additional filtering step to retain only SNPs in the 3' untranslated region (UTR) for faster runtime (this strategy is appropriate for 3'-tag sequencing protocols, but could also be adapted for 5'-tag or full-transcript sequencing); (iii) sample genotyping from matched bulk RNA-seq samples using bcftools [36]; (iv) sample genotyping from matched bulk RNA-seq samples using cellSNP [37]; and (v) sample

genotyping from scRNA-seq samples using cellSNP [37]. Scenario (ii) was used for both datasets (HGSOC and lung adenocarcinoma), and the remaining scenarios were applied to the HGSOC dataset only. Scenarios (iii) and (iv) require matched bulk RNA-seq samples, while scenarios (i) and (v) have slow runtimes. Specifically, for the HGSOC dataset, we evaluated performance across several combinations of methods for genotyping and demultiplexing (labeled as "genotypeMethod_demultiplexingMethod" in Results). For the lung adenocarcinoma dataset, we used the list of population SNPs from the 1000 Genomes Project provided by the authors of cellSNP/Vireo, filtered to retain only SNPs in the 3' UTR.

We used two datasets for the benchmark evaluations. The first dataset consists of three unique molecular identifier (UMI)-based scRNA-seq HGSOC samples measured on the 10x Genomics platform [40], obtained from separate, unrelated individuals at the Huntsman Cancer Institute at the University of Utah. We also obtained matched bulk RNA-seq samples from the same three individuals for sample genotyping. The raw data is available by controlled access via the Database of Genotypes and Phenotypes (dbGaP) (phs002262.v1.p1), and processed gene count tables are available from the Gene Expression Omnibus (GEO) (GSE158937). The second dataset consists of six UMI-based scRNA-seq higher-TMB lung adenocarcinoma samples measured on the 10x Genomics platform, previously published by [38]. Raw data for all samples in this study are available by controlled access from the European Genome-phenome Archive (EGA) (EGAD00001005054). For our study, we used six samples identified as having TMB >25 mutations / Mb (see [38], Figure 2d and Methods). **Table 1** provides a summary of the scRNA-seq datasets.

Performance was evaluated in terms of precision and recall for demultiplexing each scRNA-seq sample. We also recorded computational runtime for each step in the workflow and benchmark scenarios. Recall is defined as the proportion of true singlet cells for each sample that are identified as singlets and assigned to the correct sample. Precision is defined as the proportion of identified cells for each sample that are true singlet cells from the correct sample. Runtime was evaluated using the Unix

`date` command. We used R version 4.0.2 for random number generation and evaluation steps performed in R, and created figures using ggplot2 [41].

For our benchmark evaluation, we developed three *in silico* simulation scenarios for each dataset -- containing either 30% simulated doublets, 20% simulated doublets, or no doublets. Doublets were simulated by combining cell barcode labels from random sets of two cells in the raw sequencing reads mapped using Cell Ranger [34], so that either 30% or 20% of the final barcodes represent doublets. For example, starting with 15,202 original cells in the HGSOC dataset, 3,508 randomly selected cells were combined with 3,508 other cells to create simulated doublets, leaving 11,694 final cell barcodes, of which 3,508 (30%) represent doublets. The 30% doublets scenario represents the upper end of our planned strategy for a “super-loading” experimental design, i.e. loading multiplexed cells at extremely high concentration to reduce library preparation costs and subsequently removing identifiable doublets [5,17,29]; the 20% doublets scenario represents an intermediate super-loading scenario; and the no doublets scenario serves as a best-case baseline scenario to evaluate performance of the demultiplexing tools.

Single-cell RNA sequencing of ovarian tumors

De-identified HGSOC samples were processed after cryopreservation in liquid nitrogen where tissue chunks were stored in RPMI media with 10% fetal bovine serum and 10% DMSO. Samples were thawed and dissociated to single cells using the Miltenyi Human Tumor Dissociation Kit and the GentleMACS dissociator. Samples were incubated on the GentleMACS at 37°C for 1 hour with the setting of 1,865 rounds per run. A 70 µm MACS smart strainer was used to deplete cell doublets before loading onto the 10x Genomics Chromium Controller. Library preparation was performed using the 10x Genomics 3’ Gene Expression Library Prep v3 and libraries were sequenced on an Illumina NovaSeq instrument.

Availability of source code and requirements

All code scripts to reproduce the benchmarking evaluations, generate figures in the manuscript, and run the Snakemake workflow are freely accessible from GitHub at https://github.com/lmweber/snp-dmx-cancer. All tools used within the benchmarking evaluations and workflow are freely available, as described in Methods. Software versions used were Cell Ranger 4.0.0, bcftools 1.10.2-91-g365d117, demuxlet 3ab507c, cellsnp-lite 1.2.0, and Vireo 0.5.0.

Data availability

Raw and processed sequencing data generated in this study (HGSOC dataset) are available from the Database of Genotypes and Phenotypes (dbGaP) (raw data consisting of FASTQ files, accession phs002262.v1.p1) and Gene Expression Omnibus (GEO) (processed data files containing gene count tables, accession GSE158937). The lung adenocarcinoma dataset was previously published by [36], and is available from the European Genome-phenome Archive (EGA) (EGAD00001005054).

Acknowledgments

We thank Yuanhua Huang for assistance with running Vireo and cellSNP; Davis McCarthy for advice regarding Vireo; and attendees from the Stephanie Hicks and Kasper Hansen joint lab meetings at Johns Hopkins University for helpful feedback and discussions. We thank Hae-Ock Lee and Myung-Ju Ahn of the Samsung Medical Center for providing access to the lung adenocarcinoma dataset. Research reported in this publication utilized the Biorepository and Molecular Pathology Shared Resource and the High-Throughput Genomics Shared Resource at the Huntsman Cancer Institute at

University of Utah and was supported by NIH/NCI award P30 CA042014. The content is solely the responsibility of the authors and does not necessarily represent the official views of the NIH.

Author contributions

LMW: Software, Formal analysis, Investigation, Data curation, Writing - Original Draft, Visualization

AAH: Software

PFH: Software, Writing - Review & Editing

KCB: Investigation

JG: Investigation, Resources, Writing - Review & Editing

JAD: Resources, Writing - Review & Editing, Funding acquisition

CSG: Conceptualization, Resources, Writing - Review & Editing, Funding acquisition

SCH: Conceptualization, Resources, Writing - Original Draft, Writing - Review & Editing, Supervision, Funding acquisition

Ethics approval and consent to participate

Ovarian cancer tissue was obtained and studied under written informed consent at the Huntsman Cancer Institute through approved University of Utah Institutional Review Board protocols IRB_00010924 and IRB_00118086. Analysis of human data in this study was also approved by the University of Pennsylvania Institutional Review Board (IRB protocol 832353) and the Johns Hopkins Bloomberg School of Public Health Institutional Review Board (IRB00013099).

Competing interests

The authors declare no conflicts of interest.

Funding

LMW, AHA, KCB, JG, JAD, CSG, and SCH were supported by the National Institutes of Health grant from the National Cancer Institute R01CA237170. JAD is also supported by Huntsman Cancer Foundation and National Institutes of Health grant from the National Cancer Institute P30 CA042014 (to N. Ulrich).

References

1. Hicks SC, Townes FW, Teng M, Irizarry RA. Missing data and technical variability in single-cell RNA-sequencing experiments. Biostatistics. 2018;19: 562–578.

2. Tung P-Y, Blischak JD, Hsiao CJ, Knowles DA, Burnett JE, Pritchard JK, et al. Batch effects and the effective design of single-cell gene expression studies. Scientific Reports. 2017;7: 39921.

3. Huang Y, McCarthy DJ, Stegle O. Vireo: Bayesian demultiplexing of pooled single-cell RNA-seq data without genotype reference. Genome Biology. 2019;20: 273.

4. Xu J, Falconer C, Nguyen Q, Crawford J, McKinnon BD, Mortlock S, et al. Genotype-free demultiplexing of pooled single-cell RNA-seq. Genome Biology. 2019;20: 290.

5. Kang HM, Subramaniam M, Targ S, Nguyen M, Maliskova L, McCarthy E, et al. Multiplexed droplet single-cell RNA-sequencing using natural genetic variation. Nature Biotechnology. 2018;36: 89–94.

6. Germain P-L, Sonrel A, Robinson MD. pipeComp, a general framework for the evaluation of computational pipelines, reveals performant single cell RNA-seq preprocessing tools. Genome Biology. 2020;21.

7. Bais AS, Kostka D. scds: computational annotation of doublets in single-cell RNA sequencing data. Bioinformatics. 2019;36: 1150–1158.

8. Wolock SL, Lopez R, Klein AM. Scrublet: Computational Identification of Cell Doublets in Single-Cell Transcriptomic Data. Cell Systems. 2019;8: 281–291.e9.

9. McGinnis CS, Murrow LM, Gartner ZJ. DoubletFinder: Doublet Detection in Single-Cell RNA Sequencing Data Using Artificial Nearest Neighbors. Cell Systems. 2019;8: 329–337.e4.

10. Lun ATL, McCarthy DJ, Marioni JC. A step-by-step workflow for low-level analysis of single-cell RNA-seq data with Bioconductor. F1000Research. 2016;5: 2122.

11. Farouni R, Djambazian H, Ferri LE, Ragoussis J, Najafabadi HS. Model-based analysis of sample index hopping reveals its widespread artifacts in multiplexed single-cell RNA-sequencing. Nature Communications. 2020;11: 2704.

12. Griffiths JA, Richard AC, Bach K, Lun ATL, Marioni JC. Detection and removal of barcode swapping in single-cell RNA-seq data. Nature Communications. 2018;9: 2667.

13. Costello M, Fleharty M, Abreu J, Farjoun Y, Ferriera S, Holmes L, et al. Characterization and remediation of sample index swaps by non-redundant dual indexing on massively parallel sequencing platforms. BMC Genomics. 2018;19: 332.

14. Sinha R, Stanley G, Gulati GS, Ezran C, Travaglini KJ. Index switching causes “spreading-of-signal” among multiplexed samples in Illumina HiSeq 4000 DNA sequencing. bioRxiv (https://www.biorxiv.org/content/101101/125724v1) (doi: https://doi.org/101101/125724). 2017.

15. Illumina. Effects of index misassignment on multiplexing and downstream analysis. White Paper (https://www.illumina.com/content/dam/illumina-marketing/documents/products/whitepapers/index-

hopping-white-paper-770-2017-004.pdf). 2018.

16. McGinnis CS, Patterson DM, Winkler J, Conrad DN, Hein MY, Srivastava V, et al. MULTI-seq: sample multiplexing for single-cell RNA sequencing using lipid-tagged indices. Nature Methods. 2019;16: 619–626.

17. Stoeckius M, Zheng S, Houck-Loomis B, Hao S, Yeung BZ, Mauck WM 3rd, et al. Cell Hashing with barcoded antibodies enables multiplexing and doublet detection for single cell genomics. Genome Biology. 2018;19: 224.

18. Xin H, Lian Q, Jiang Y, Luo J, Wang X, Erb C, et al. GMM-Demux: sample demultiplexing, multiplet detection, experiment planning, and novel cell-type verification in single cell sequencing. Genome Biology. 2020;21.

19. Heaton H, Talman AM, Knights A, Imaz M, Gaffney DJ, Durbin R, et al. Souporcell: robust clustering of single-cell RNA-seq data by genotype without reference genotypes. Nature Methods. 2020;17: 615–620.

20. Zhang F, Kang HM. popscle: A suite of population scale analysis tools for single-cell genomics data (freemuxlet). Software package (https://github.com/statgen/popscle). 2020.

21. 1000 Genomes Project Consortium, Auton A, Brooks LD, Durbin RM, Garrison EP, Kang HM, et al. A global reference for human genetic variation. Nature. 2015;526: 68–74.

22. McFarland JM, Paolella BR, Warren A, Geiger-Schuller K, Shibue T, Rothberg M, et al. Multiplexed single-cell transcriptional response profiling to define cancer vulnerabilities and therapeutic mechanism of action. Nature Communications. 2020;11: 4296.

23. Kinker GS, Greenwald AC, Tal R, Orlova Z, Cuoco MS, McFarland JM, et al. Pan-cancer single-cell RNA-seq identifies recurring programs of cellular heterogeneity. Nature Genetics. 2020;52: 1208–1218.

24. Spencer DH, Zhang B, Pfeifer J. Chapter 8 - Single Nucleotide Variant Detection Using Next Generation Sequencing. In: Kulkarni S, Pfeifer J, editors. Clinical Genomics. 2015. pp. 109–127.

25. ICGC/TCGA Pan-Cancer Analysis of Whole Genomes Consortium. Pan-cancer analysis of whole genomes. Nature. 2020;578: 82–93.

26. Fancello L, Gandini S, Pelicci PG, Mazzarella L. Tumor mutational burden quantification from targeted gene panels: major advancements and challenges. Journal for Immunotherapy of Cancer. 2019;7: 183.

27. Chalmers ZR, Connelly CF, Fabrizio D, Gay L, Ali SM, Ennis R, et al. Analysis of 100,000 human cancer genomes reveals the landscape of tumor mutational burden. Genome Medicine. 2017;9: 34.

28. Sherry ST, Ward MH, Kholodov M, Baker J, Phan L, Smigielski EM, et al. dbSNP: the NCBI database of genetic variation. Nucleic Acids Research. 2001;29: 308–311.

29. Hafemeister C, Satija R. Cost Per Cell: Multiplexing cost calculator. Website

(https://satijalab.org/costpercell). 2018.

30. Regev A, Teichmann SA, Lander ES, Amit I, Benoist C, Birney E, et al. The Human Cell Atlas. eLife. 2017;6: 1–30.

31. Taylor DM, Aronow BJ, Tan K, Bernt K, Salomonis N, Greene CS, et al. The Pediatric Cell Atlas: Defining the Growth Phase of Human Development at Single-Cell Resolution. Developmental Cell. 2019;49: 10–29.

32. Ciriello G, Miller ML, Aksoy BA, Senbabaoglu Y, Schultz N, Sander C. Emerging landscape of oncogenic signatures across human cancers. Nature Genetics. 2013;45: 1127–1133.

33. Köster J, Rahmann S. Snakemake — a scalable bioinformatics workflow engine. Bioinformatics. 2012;28: 2520–2522.

34. 10x Genomics. Cell Ranger. Software (https://support10xgenomics.com/single-cell-gene-expression/software/overview/welcome). 2020.

35. Li H, Handsaker B, Wysoker A, Fennell T, Ruan J, Homer N, et al. The Sequence Alignment/Map format and SAMtools. Bioinformatics. 2009;25: 2078–2079.

36. Li H. A statistical framework for SNP calling, mutation discovery, association mapping and population genetical parameter estimation from sequencing data. Bioinformatics. 2011;27: 2987–2993.

37. Huang Y. cellSNP. Software package (https://github.com/single-cell-genetics/cellSNP). 2020.

38. Kim N, Kim HK, Lee K, Hong Y, Cho JH, Choi JW, et al. Single-cell RNA sequencing demonstrates the molecular and cellular reprogramming of metastatic lung adenocarcinoma. Nature Communications. 2020;11: 2285.

39. Srivastava A, Malik L, Smith T, Sudbery I, Patro R. Alevin efficiently estimates accurate gene abundances from dscRNA-seq data. Genome Biology. 2019;20: 65.

40. Zheng GXY, Terry JM, Belgrader P, Ryvkin P, Bent ZW, Wilson R, et al. Massively parallel digital transcriptional profiling of single cells. Nature Communications. 2017;8: 14049.

41. Wickham H. ggplot2: Elegant Graphics for Data Analysis. Springer-Verlag New York; 2016.

Supplementary Figures

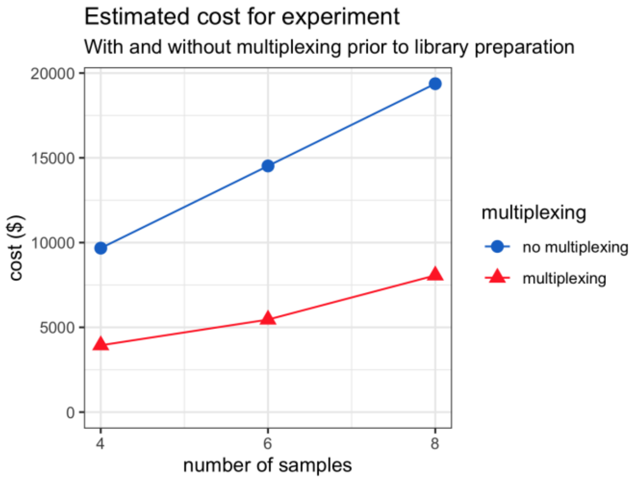

Supplementary Figure 1. Illustration of expected cost savings from multiplexed experimental design prior to library preparation. The figure shows the total of estimated library preparation and sequencing costs, with either no multiplexing or full multiplexing (all samples prepared as a single library and sequenced together), for experiments with 4, 6, or 8 samples. The calculations assume 4,000 desired cells per sample after demultiplexing, after discarding identifiable doublets consisting of cells from multiple samples; library preparation costs of $2,000 per sample or multiplexed set of samples; sequencing costs of $1,500 per 400 million reads with an additional 30% cost due to unaligned reads and adapters; and approximately 20,000 reads per cell. Calculations were performed using the “Cost Per Cell” online calculator provided by the Satija Lab [29].

Figure 1

Click here to access/download;Figure;Figure1_wo

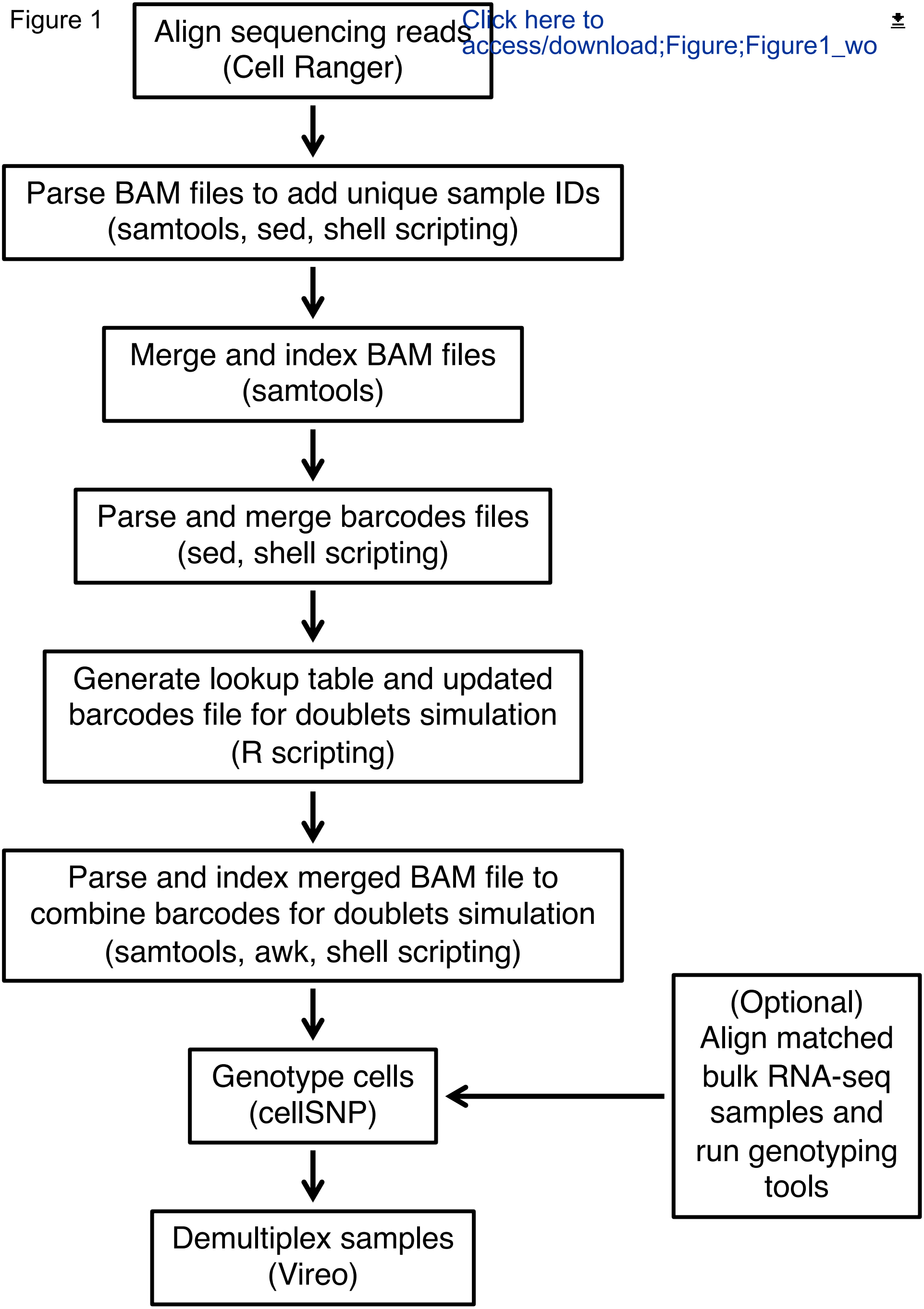

Figure 2

Click here to access/download;Figure;Figure2_precision_recall.pdf

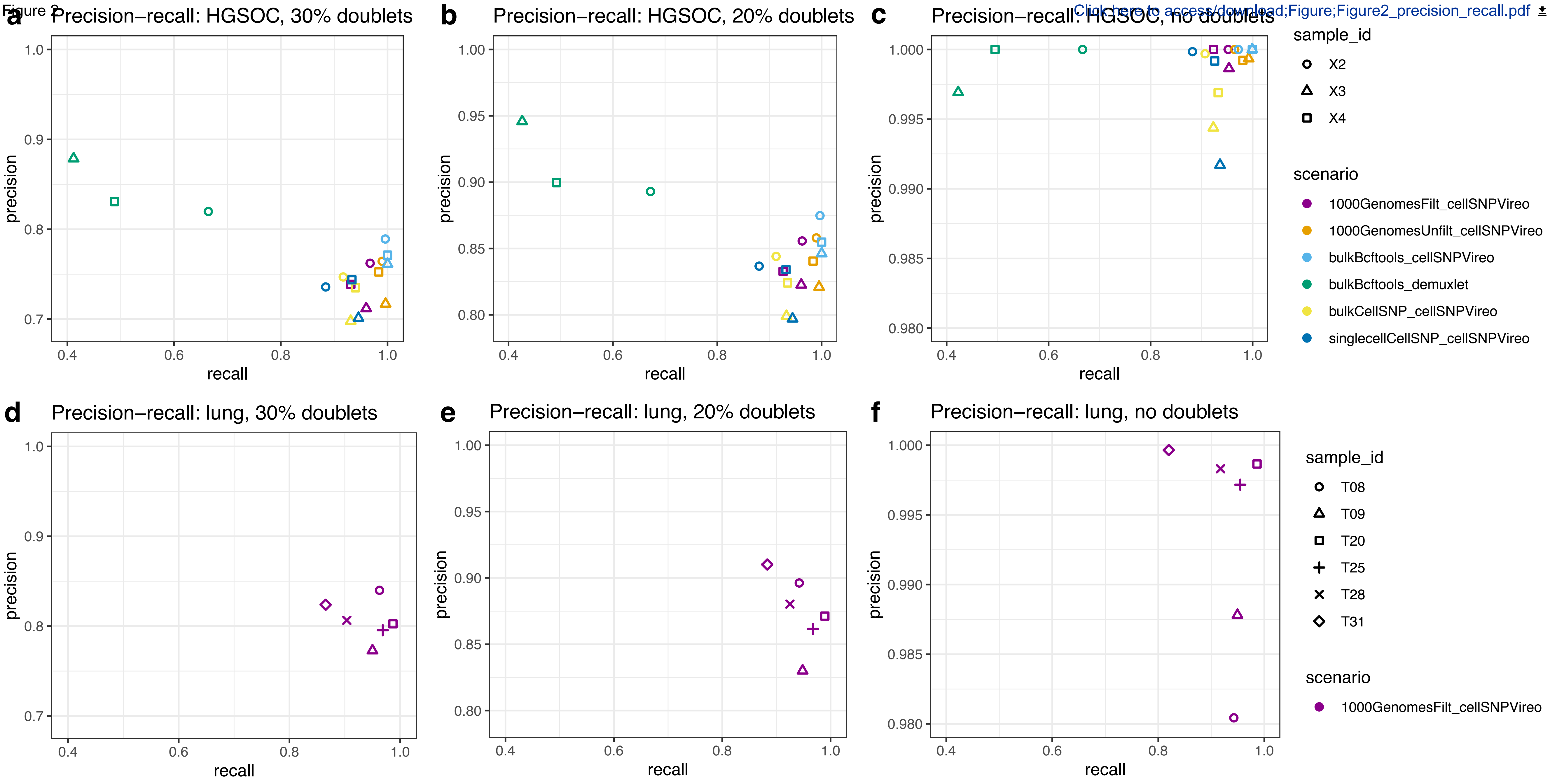

Figure 3

Click here to access/download;Figure;Figure3_runtimes.pdf

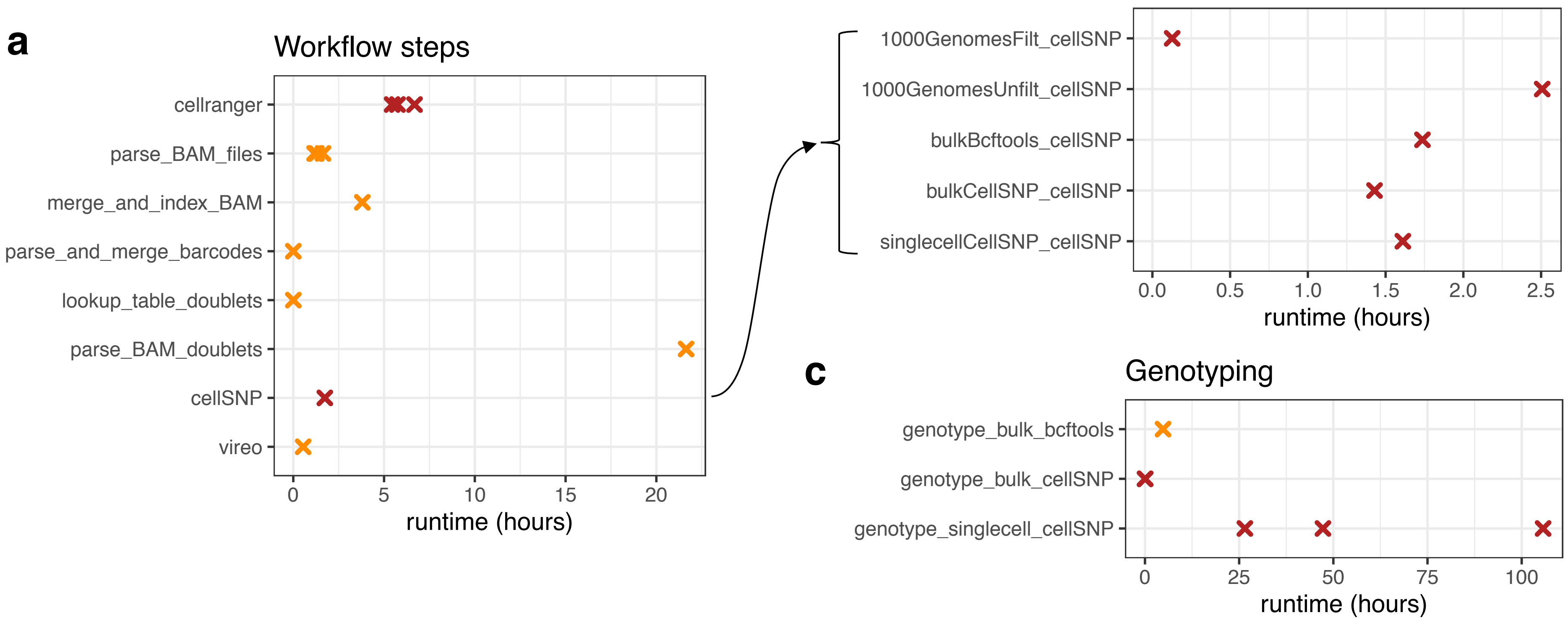

Supplementary Material - Figure S1

Click here to access/download
Supplementary Material
Figure_S1_cost_savings.pdf

Personal Cover Click here to access/download;Personal Cover;In_silico_demultiplexing_cover_letter.pdf

Simon Edmunds, PhD
Editor-in-Chief, *GigaScience*

Dear Dr. Edmunds,

Please find enclosed our manuscript submission titled "Genetic demultiplexing of pooled single-cell RNA-sequencing samples in cancer facilitates effective experimental design", for consideration by *GigaScience* as a Research article.

Recent experimental designs have been proposed for single-cell RNA-sequencing (scRNA-seq) where tissue samples are pooled prior to library preparation (instead of after library preparation and prior to sequencing, which is the standard approach in scRNA-seq). Previous work has shown computational demultiplexing algorithms (e.g. Vireo[1] and demuxlet[2]) for this experimental design work well in healthy tissues as well as cancer cell lines from distinct cancer types. Advantages of these demultiplexing algorithms include significant cost savings during library preparation, as well as the ability to reduce batch effects as samples are not processed in separate libraries. However, to the best of our knowledge, this has not yet been shown for the most difficult setting of pooled cancer samples from multiple individuals within the same cancer type, where additional somatic variants might adversely affect the demultiplexing performance.

To address this, here we investigate whether these recently developed computational demultiplexing methods that rely on genetic variation between biological samples from different individuals can be used with these new experimental designs for scRNA-seq in the cancer setting.
Using the raw sequencing scRNA-seq reads from two cancer types with high copy number variation (CNV) and high single nucleotide variation (SNV) burden (HGSOC and lung adenocarcinoma, respectively), we performed a series of simulations with doublets (two cells per droplet) that are likely to occur when overloading the sequencing machines with combined samples. Our evaluations showed excellent demultiplexing performance (recall: 99.9% and precision: 85.9%, averaged across 3 samples and assuming 20% doublets), especially when matched bulk RNA-seq samples are available for genotyping. We believe this is a useful result for readers of GigaScience and the larger single-cell community as it will give researchers the justification to proceed with genetic variation-based demultiplexing designs for scRNA-seq experiments in cancer.

In addition, we have taken great care to develop a reproducible simulation framework and analysis workflow for our benchmark evaluations. This includes a Snakemake workflow consisting of the best-performing set of tools identified in our benchmark evaluation (which can be run with a single command on a Linux cluster), as well as reproducible scripts for all other benchmark scenarios and evaluations. We provide these resources to allow other researchers to adapt our framework to perform similar pilot evaluations for experimental design planning purposes in their own experiments. Our workflow is modular, allowing alternative tools to be substituted. All code is freely available from our code repository on GitHub (https://github.com/lmweber/snp-dmx-cancer).

---

[1] Huang et al. (2019), Genome Biology: https://pubmed.ncbi.nlm.nih.gov/31836005/
[2] Kang et al. (2018), Nature Biotechnology: https://pubmed.ncbi.nlm.nih.gov/29227470/

The raw data files (FASTQ files) used as input for our simulations contain patient-identifiable information (genetic variants), and are available by controlled access from the Database of Genotypes and Phenotypes (dbGaP) (accession phs002262.v1.p1) and the European Genome-phenome Archive (EGA) (EGAD00001005054). In addition, we have made non-identifiable gene count tables publicly available from the Gene Expression Omnibus (GEO) (GSE158937).

All authors have approved the manuscript for publication, and declare that they do not have any competing interests. The manuscript is not currently under consideration for publication with any other journal.

We are confident that your readers will find this study to be a compelling and scientifically useful result for the single-cell and cancer research communities, as well as a useful and adaptable simulation framework and code and data resource, and will therefore find the submitted manuscript highly relevant.

Thank you for your consideration of the manuscript.

Best wishes,

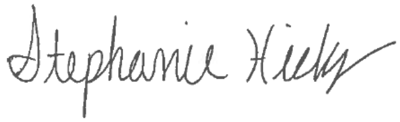

Stephanie Hicks
Department of Biostatistics
Johns Hopkins Bloomberg School of Public Health
Johns Hopkins Data Science Lab
