# Supplementary material for: Genetic demultiplexing of pooled single-cell RNA-sequencing samples in cancer facilitates effective experimental design
Source: Gigascience. 2021 Sep 22;10(9):giab062. doi: 10.1093/gigascience/giab062 (PMC8458035; doi:10.1093/gigascience/giab062)
Supplement: giab062_GIGA-D-21-00074_Revision_1 [file giab062_giga-d-21-00074_revision_1.pdf]

GigaScience

Genetic demultiplexing of pooled single-cell RNA-sequencing samples in cancer facilitates effective experimental design

--Manuscript Draft--

**Manuscript Number:**	GIGA-D-21-00074R1
**Full Title:**	Genetic demultiplexing of pooled single-cell RNA-sequencing samples in cancer facilitates effective experimental design
**Article Type:**	Research
**Funding Information:**	Foundation for the National Institutes of Health (R01CA237170) — Dr Casey S Greene<br>Foundation for the National Institutes of Health (P30 CA042014) — Dr Jennifer Anne Doherty
**Abstract:**	Abstract<br><br>Background: Pooling cells from multiple biological samples prior to library preparation within the same single-cell RNA sequencing experiment provides several advantages, including lower library preparation costs and reduced unwanted technological variation, such as batch effects. Computational demultiplexing tools based on natural genetic variation between individuals provide a simple approach to demultiplex samples, which does not require complex additional experimental procedures. However, these tools have not been evaluated in cancer, where somatic variants, which could differ between cells from the same sample, may obscure the signal in natural genetic variation.<br>Results: Here, we performed in silico benchmark evaluations by combining raw sequencing reads from multiple single-cell samples in high-grade serous ovarian cancer, which has a high copy number burden, and lung adenocarcinoma, which has a high tumor mutational burden. Our results confirm that genetic demultiplexing tools can be effectively deployed on cancer tissue using a pooled experimental design, although high proportions of ambient RNA from cell debris reduce performance.<br>Conclusions: This strategy provides significant cost savings through pooled library preparation. To facilitate similar analyses at the experimental design phase, we provide freely accessible code and a reproducible Snakemake workflow built around the best-performing tools found in our in silico benchmark evaluations, available at https://github.com/lmweber/snp-dmx-cancer.
**Corresponding Author:**	Stephanie Hicks<br>Johns Hopkins University Bloomberg School of Public Health<br>Baltimor, UNITED STATES
**Corresponding Author Secondary Information:**	
**Corresponding Author's Institution:**	Johns Hopkins University Bloomberg School of Public Health
**Corresponding Author's Secondary Institution:**	
**First Author:**	Lukas M Weber
**First Author Secondary Information:**	
**Order of Authors:**	Lukas M Weber<br>Ariel A Hippen<br>Peter F Hickey<br>Kristofer C Berrett<br>Jason Gertz<br>Jennifer Anne Doherty

Powered by Editorial Manager® and ProduXion Manager® from Aries Systems Corporation

	Casey S Greene
	Stephanie C Hicks
**Order of Authors Secondary Information:**	
**Response to Reviewers:**	Response to Reviewers

Reviewer 1:

Reviewer #1: The authors have investigated methods to demultiplex samples from single-cell RNA sequencing data based on SNPs. Pooling of cancer samples is certainly an important tool for cost reduction and minimizing batch effects as the authors have pointed out. It nice to see the well documented, freely available github repository.

Major Points:

The authors have tested demultiplexing based on matched reference data, publicly available SNPs and without any SNP reference. It has to be noted however, that without a matching reference the samples can be demultiplexed but not assigned to a specific donor. This has to be mentioned in the text as this is a crucial feature.

This point has now been mentioned more clearly in the Introduction (paragraph 2) and Discussion (final paragraph).

In the cancer context it would be important to test also reference data from a SNP array in addition to bulk RNA-seq data. It would be interesting to see which matched reference would perform better. Could the authors provide such an analysis for the HGSOC dataset?

This is an important point, which we had not previously considered. Since we did not have access to SNP array data for the HGSOC samples, we designed an additional simulation strategy based on the existing samples, to test the performance when using a SNP array. In brief, we found that using reference data from a SNP array performs remarkably well and we describe the additional analyses and figures that have now been added to the manuscript in response to this excellent suggestion.

We downloaded the set of SNPs from a widely used SNP array (the Infinium Multi-Ethnic Global-8 v1.0 array from the Multi-Ethnic Global Array or MEGA Consortium, which includes 1.7 million SNPs), and then calculated the sets of overlapping SNPs between the SNP array and (i) our bulk RNA-seq genotype reference (605,367 total SNPs before calculating overlap) and (ii) the 1000 Genomes reference filtered to 3' UTRs (84,853 total SNPs before calculating overlap). Then, we re-ran the benchmark evaluations using these smaller sets of overlapping SNPs. We note that this strategy effectively simulates a worst-case scenario for the SNP array, since we are using only a small subset of the SNPs from the array (stating this another way, we anticipate the following results would improve if we had access to the full 1.7 million SNP array). Compared to the SNP array, the overlapping sets contain 2.6% of SNPs (bulk RNA-seq) or 0.8% of SNPs (1000 Genomes filtered); while compared to our original references the overlapping sets contain 7.6% (bulk RNA-seq) or 16.5% (1000 Genomes filtered) of the original SNPs (see Supplementary Table 1 for a summary of the set sizes).

Despite the extreme reduction in number of SNPs compared to the full array or the original references, demultiplexing performance for cellSNP/Vireo remains remarkably high, especially when using the bulk RNA-seq reference SNPs. However, the performance of demuxlet drops substantially. (See Supplementary Figure 3; Results and Methods). Since we are using only a small subset of the array SNPs, these results give us confidence that good demultiplexing performance can be achieved when using SNP arrays in the cancer context.

Powered by Editorial Manager® and ProduXion Manager® from Aries Systems Corporation

The increased robustness of cellSNP/Vireo compared to demuxlet is also consistent with our observations for the additional supplementary analyses, e.g. adding ambient RNA from simulated debris or lysed cells, as described below in comments for Reviewer 2.

Could the authors run a real demultiplex experiment in addition to mixing reads in silico (e.g. for the HGSOC samples)?

We thank the reviewer for this comment and agree an experimental evaluation (as opposed to an in silico evaluation) would strengthen the results. Unfortunately, it was not possible to generate additional experimental data within the revision timeline for this manuscript. However, we have now included additional analyses in our revised manuscript that significantly strengthen the evidence for our central claim: genetic variation-based demultiplexing works well in the cancer context.

In particular, this claim is supported by the additional results of (i) subset of SNPs from SNP array (see above), (ii) simulated ambient RNA from debris or lysed cells (see comments for Reviewer 2 below), and (iii) confirmation of baseline performance in non-cancer data (comments for Reviewer 2), all of which demonstrate that cellSNP/Vireo maintain performance in settings that we had not previously tested.

Finally, we have now expanded the Discussion to note that additional experimental data could be used to further strengthen these claims, if it becomes available. (We have also mentioned that the main difficulty when working with experimental data will be in determining a reliable ground truth.)

Can the authors comment on the number of doublets identified with their technique and run at least one doublet detection tool in comparison? For HGSOC (n=3) half of all doublet should be mixed doublets and half harbours cells from the same sample.

We have added additional results and discussion to address this point, which was also raised by Reviewer 2. (Previously, this information was only included implicitly within the precision and recall calculations.) In Supplementary Tables 3 and 4, we have included tables comparing the true and false doublet calls by sample for the top-performing set of tools (cellSNP/Vireo with bulk RNA-seq reference) and the comparison with demuxlet (with bulk RNA-seq reference) for the HGSOC dataset with 30% doublets. This shows that the doublet calls from cellSNP/Vireo are relatively pure (average of 99.2% true identifiable doublets among called doublets), while the reduction in overall precision is due to additional true identifiable doublets incorrectly called as singlets. By contrast, demuxlet returns a much lower percentage (31.9%) of true identifiable doublets among the doublet calls.

We also ran a recent doublet detection tool (scDblFinder; https://bioconductor.org/packages/scDblFinder) as an illustration to see if we could use these tools to identify any remaining doublets after demultiplexing. However, this did not perform well, giving large proportions of both false positives and false negatives (Supplementary Table 5). We hypothesize that this may be due to the nature of cancer samples, where clustering on expression profiles returns more varied clusters than in non-cancer data. While there are benchmark evaluations of doublet detection tools in the non-cancer setting (e.g. https://doi.org/10.1016/j.cels.2020.11.008), we feel a comprehensive evaluation of doublet detection tools applied here (e.g. using interactive plots and investigating additional possible reasons for reduced performance in cancer compared to non-cancer data) is outside the scope of this manuscript. Finally, we have extended the Discussion to mention these limitations more clearly, and to suggest directions for future work in this area.

Minor points:

In the discussion the authors state that they compared the best performing tools against alternative tools (page 15, line 344) - however it is essentially cellSNPVireo vs demuxlet (one vs another). Should be clearer in the text.

Powered by Editorial Manager® and ProduXion Manager® from Aries Systems Corporation

We have clarified this sentence, and expanded the Introduction and Discussion to make it more clear that we are comparing these two tools, and not performing a comprehensive benchmark for all available tools.

Can the authors comment on hashing and how hashing would perform in comparison to genetic demultiplexing in the discussion?

In general, we would expect cell hashing to be the gold standard in terms of performance, while the performance of genetic variation-based demultiplexing depends largely on the computational tool used and the quality of the genotype reference. However, genetic variation-based demultiplexing also provides significant advantages in terms of simpler sample preparation and cost savings during library preparation. We have added additional text to expand on this in the Discussion, and have also mentioned cell hashing approaches in the Introduction.

Reviewer 2:

Reviewer #2: This paper addresses an important and untested question of whether single cell genotype demultiplexing works on cancer samples.

1. The authors use in-silico mixtures of individual single cell cancer experiments with varying percentages of doublet cell barcodes. While it is understandable due to the "finite and irreplaceable nature of tumor cells", it potentially reduces the value, accuracy, and applicability to real data especially due to my next issue. To me this is not a deal breaker, but a distinct limitation.

We agree that demonstrating demultiplexing performance on real experimental data would further strengthen our conclusions, compared to relying on simulations. However, while acknowledging this limitation, our expanded set of simulation scenarios provides strong evidence for our central claim that genetic variation-based demultiplexing tools perform well for cancer data.

In particular, as mentioned above for Reviewer 1, our additional supplementary analyses on (i) using a subset of overlapping SNPs from the MEGA SNP array (see comments for Reviewer 1), (ii) ambient RNA from debris or lysed cells, and (iii) confirmation of performance in healthy samples, significantly strengthen our evidence for our conclusions compared to our original manuscript. We thank the reviewers for these suggestions.

We have also ensured that our wording in the Introduction and Discussion makes it clear that our results are based on simulations generated by modifying real data.

2. Ambient RNA from lysed cells prior to partitioning is not addressed in either simulation or discussion. I think that it should be at least discussed as necrosis is not uncommon in cancer samples. It is important to note that as ambient RNA increases, demuxlet quickly begins classifying almost everything as a doublet while other methods (vireo, souporcell) are more robust to this type of noise.

This was an important omission in our previous in silico benchmark evaluation, and we thank the reviewer for this suggestion. We have now added additional scenarios that include ambient RNA from simulated cell debris or lysed cells by computationally assigning all sequencing reads from a certain percentage of cell barcodes (10%, 20%, or 40%) to other randomly selected cell barcodes, and re-running the demultiplexing tools (results included as new Figure 2 and Supplementary Figures 1-2). These results demonstrate that overall demultiplexing performance decreases in terms of recall, although the effect is minimized when using the top-performing set of tools (cellSNP/Vireo with bulk RNA-seq reference). As suggested above, the performance of demuxlet drops much more substantially, suggesting that cellSNP/Vireo is more robust to this type of noise. We have included additional discussion of this issue in Results

and Discussion. In particular, this suggests the importance of considering experimental techniques such as straining to reduce cell debris in cancer samples, when possible.

3. A full normal sample is not shown for comparison to cancer samples. This could be taken from free available datasets. For instance the Hipsci datasets from my souporcell paper could be used. You would want to try to normalize for UMI/cell with subsampling and match roughly the number of cells per individual and then of course match the doublet rate. The number and type of cancer samples tested are fairly limited but adequate for an initial evaluation.

We thank the reviewer for this comment. We have now incorporated additional simulations based on the five individual iPSC cell lines in the souporcell paper (https://doi.org/10.1038/s41592-020-0820-1), as a healthy (non-cancer) baseline comparison, including both 20% and 30% doublet scenarios. Normalizing the UMI counts per sample became too computationally intensive, so we have instead reported additional details (number of cells per sample, detected genes per cell, UMI counts per cell) for all three datasets (HGSOC, lung, cell line) (Supplementary Table 2) to ensure that comprehensive information is provided for readers to compare the datasets. Performance in the cell line data (Supplementary Figure 4) was comparable to the main benchmark scenarios, giving us confidence that our simulation framework can be reliably applied in both cancer and non-cancer settings, and providing a useful additional baseline comparison that is consistent with previous published results in non-cancer data.

3. I think the author's results actually warrant a stronger conclusion in the question of fully supervised and genotype model based methods (demuxlet, which assumes allele fractions must match the stated genotype) vs cluster center based and thus free allele fraction methods (vireo, souporcell etc). This also has a theoretical advantage in cancer samples which will have non-standard allele fractions around copy number alterations and somatic mutations. Vireo clearly outperforms demuxlet in accurate doublet detection (demuxlet's rigid model system results in over-calling doublets which is presumably what is crushing demuxlet's recall in some samples).

We have now provided additional details on performance in terms of types of doublet calls for both cellSNP/Vireo and demuxlet (Supplementary Tables 3 and 4), as well as the additional results for ambient RNA and subsets of SNPs from an array (Figure 2 and Supplementary Figures 1-3). As suggested by the reviewer, cellSNP/Vireo consistently outperforms demuxlet by a wide margin, especially in these more difficult simulation scenarios. We have included additional wording in Results and Discussion to make these comparisons between the two tools more clear for readers.

4. The paper mentions that the authors prefer high recall over precision but does not discuss the potential downsides of low precision.

We have included additional text to clarify this. Specifically, we prefer to retain all true singlet cells (high recall) if possible, since there is the possibility of applying additional downstream analyses (visualizations, doublet detection tools) to identify any remaining doublets (which is one of the possible sources of reduced precision). In addition, we have included illustrative tables of results comparing the types of doublet calls for both cellSNP/Vireo (top-performing scenario with bulk RNA-seq reference, HGSOC, 30% doublets; Supplementary Table 3) and demuxlet (with bulk RNA-seq reference; Supplementary Table 4), as suggested below, which provides additional information on the types of errors. As suggested by Reviewer 1, we also ran a recent doublet detection tool (scDblFinder; https://bioconductor.org/packages/scDblFinder), but this did not perform well, giving large proportions of both false positives and false negatives (Supplementary Table 5), possibly due to the more varied expression in cancer data compared to non-cancer affecting clustering performance. While benchmarks of doublet detection tools exist for non-cancer data (e.g. https://doi.org/10.1016/j.cels.2020.11.008), in our view a comprehensive evaluation of doublet detection tools applied here is outside the scope of this manuscript.

Powered by Editorial Manager® and ProduXion Manager® from Aries Systems Corporation

	5. Most importantly I think it would also be useful to discuss how low recall and precision could be attained and in outlier samples discuss which error modes were observed. You could attain low recall due to 1. high unassigned cells. 2. overcalling doublets. 3. misassigned cells. And you could attain low precision through 1. misassigned cells 2. undercalling doublets. These different error modes are very different and could affect downstream processing and inference in different ways. I think that it is important to tease out which of these phenomenon is going on with each sample. I grant that it does not lend itself to standard statistical metrics such as precision/recall as there are more than 2 states of truth (single cell correct sample, single cell incorrect sample, doublet cell). A confusion matrix heatmap would be one option but showing this for many samples would be information overload. At the very least a discussion of the outlier samples should have a breakdown of these numbers with a discussion of why this happens (as explained previously). This would be useful for the demuxlet runs on HGSDC explaining low recall and for the relatively low recall on all runs for the HGSDC 30% doublet run.<br><br>We thank the reviewer for this suggestion. This information was previously only implicitly contained within the precision and recall calculations, and we agree it is informative to provide an illustration of these error modes for readers. We have included tables of results comparing the types of true and false singlet and doublet calls for two illustrative scenarios: the top-performing set of tools (cellSNP/Vireo with bulk RNA-seq reference) in HGSOC with 30% doublets (Supplementary Table 3), and the comparison for demuxlet (with bulk RNA-seq reference, HGSOC, 30% doublets) (Supplementary Table 4). This illustrates the differences in the types of doublet calls between the two tools. For cellSNP/Vireo, the doublet calls are relatively pure, with 99.2% of called doublets being true identifiable doublets. By contrast, for demuxlet, this percentage is much lower at 31.9%. These tables also illustrate the distinction between identifiable doublets (two cells from different donors, with different SNP profiles) and non-identifiable doublets (two cells from the same donor, which cannot be distinguished by their SNP profiles). For cellSNP/Vireo, almost all the non-identifiable doublets are assigned to the correct individual sample, while for demuxlet these contain additional false positive doublet calls.<br><br>6. One specific comment I have is with regards to the following sentence. "More fundamentally, due to the reliance on genetically distinct SNP profiles, genetic demultiplexing tools are expected to work well for human samples from unrelated individuals, but are not applicable to biological samples from inbred mice or hereditary related human populations, or samples from the same individual [3]" Almost all of this is true, but in our souporcell paper we demultiplexed a maternal/fetal sample so these tools are applicable to related humans. Of course power decreases the more related the individuals are (in the case of inbreeding). Perhaps the wording could be changed.<br><br>We have re-worded this sentence to clarify this issue, as suggested.
**Additional Information:**	
**Question**	**Response**
Are you submitting this manuscript to a special series or article collection?	No
**Experimental design and statistics**<br><br>Full details of the experimental design and statistical methods used should be given in the Methods section, as detailed in our Minimum Standards Reporting Checklist. Information essential to interpreting the data presented should be made available in the figure legends.	Yes

Have you included all the information requested in your manuscript?	
**Resources**<br><br>A description of all resources used, including antibodies, cell lines, animals and software tools, with enough information to allow them to be uniquely identified, should be included in the Methods section. Authors are strongly encouraged to cite Research Resource Identifiers (RRIDs) for antibodies, model organisms and tools, where possible.<br><br>Have you included the information requested as detailed in our Minimum Standards Reporting Checklist?	Yes
**Availability of data and materials**<br><br>All datasets and code on which the conclusions of the paper rely must be either included in your submission or deposited in publicly available repositories (where available and ethically appropriate), referencing such data using a unique identifier in the references and in the "Availability of Data and Materials" section of your manuscript.<br><br>Have you have met the above requirement as detailed in our Minimum Standards Reporting Checklist?	Yes

Powered by Editorial Manager® and ProduXion Manager® from Aries Systems Corporation

Manuscript

Click here to access/download;Manuscript;In_silico_SNP_demultiplexing_for_

Genetic demultiplexing of pooled single-cell RNA-sequencing samples in cancer facilitates effective experimental design

Lukas M. Weber[1], Ariel A. Hippen[2], Peter F. Hickey[3], Kristofer C. Berrett[4], Jason Gertz[4], Jennifer Anne Doherty[4], Casey S. Greene[5], Stephanie C. Hicks[1]*

ORCIDs:
Lukas M. Weber [0000-0002-3282-1730];
Ariel A. Hippen [0000-0001-9336-6543];
Peter F. Hickey [0000-0002-8153-6258];
Kristofer C. Berrett [0000-0002-6330-5181];
Jason Gertz [0000-0001-7568-6789];
Jennifer Anne Doherty [0000-0002-1454-8187];
Casey S. Greene [0000-0001-8713-9213];
Stephanie C. Hicks [0000-0002-7858-0231];

[1] Department of Biostatistics, Johns Hopkins Bloomberg School of Public Health, Baltimore, MD, USA
[2] Department of Systems Pharmacology and Translational Therapeutics, Perelman School of Medicine, University of Pennsylvania, PA, USA
[3] Advanced Technology & Biology Division, Walter and Eliza Hall Institute of Medical Research, Melbourne, Australia
[4] Huntsman Cancer Institute and Department of Population Health Sciences, University of Utah, UT, USA
[5] Department of Biochemistry and Molecular Genetics, University of Colorado School of Medicine, CO, USA

* Corresponding author

Keywords: genetic demultiplexing, single-cell RNA sequencing, cancer, high-grade serous ovarian cancer, lung adenocarcinoma, tumor mutational burden, computational methods, simulations, benchmarking

Date: 19 July 2021

Abstract

Background: Pooling cells from multiple biological samples prior to library preparation within the same single-cell RNA sequencing experiment provides several advantages, including lower library preparation costs and reduced unwanted technological variation, such as batch effects. Computational demultiplexing tools based on natural genetic variation between individuals provide a simple approach to demultiplex samples, which does not require complex additional experimental procedures. However, these tools have not been evaluated in cancer, where somatic variants, which could differ between cells from the same sample, may obscure the signal in natural genetic variation. **Results:** Here, we performed *in silico* benchmark evaluations by combining raw sequencing reads from multiple single-cell samples in high-grade serous ovarian cancer, which has a high copy number burden, and lung adenocarcinoma, which has a high tumor mutational burden. Our results confirm that genetic demultiplexing tools can be effectively deployed on cancer tissue using a pooled experimental design, although high proportions of ambient RNA from cell debris reduce performance. **Conclusions:** This strategy provides significant cost savings through pooled library preparation. To facilitate similar analyses at the experimental design phase, we provide freely accessible code and a reproducible Snakemake workflow built around the best-performing tools found in our *in silico* benchmark evaluations, available at https://github.com/lmweber/snp-dmx-cancer.

Introduction

Sample pooling prior to library preparation is an effective strategy for experimental design in single-cell RNA sequencing (scRNA-seq) studies, which allows researchers to assess and address unwanted technological variation such as batch effects [1,2] and reduces library preparation costs [3–5]. Several strategies involve pooling cells, labeled or otherwise identifiable in some way, from multiple biological samples, followed by combined library preparation and sequencing, and computational demultiplexing to recover the sample identities of each cell. While sample pooling creates doublets consisting of cells from multiple individuals, with the doublet rate depending on the concentration of loaded cells [5], demultiplexing approaches can also identify doublets at the demultiplexing step without relying on downstream doublet identification tools [6–10]. Depending on the method used, these techniques can also avoid the phenomenon of sample index swapping, which occurs when individually prepared libraries are subsequently pooled for sequencing [11–15].

Existing demultiplexing approaches differ in their experimental procedures, computational methodology for demultiplexing, and demultiplexing accuracy. In barcoding-based approaches (e.g. MULTI-seq [16] and cell hashing [17], and GMM-Demux for doublet identification [18]), cells are experimentally tagged with universal oligonucleotides or antibodies together with sample-specific labels, which can give highly accurate demultiplexing performance. However, these approaches make sample preparation more complex, and increase costs due to reagent purchases as well as additional library preparation and sequencing. Alternatively, genetic variation-based approaches rely only on natural genetic variation between samples from different individuals (such as single nucleotide polymorphisms, SNPs), which does not require additional experimental procedures at the single-cell level. Initial genetic variation-based demultiplexing methods, such as demuxlet [5], require a known genotype reference for each sample obtained using SNP arrays, whole exome sequencing, or bulk RNA sequencing. Recently, new methods have been developed, such as Vireo [3], scSplit [4], souporcell [19], and freemuxlet [20], which can use probabilistic models to infer the genotype directly from the single-cell reads. Note that

without an external genotype reference, these methods can demultiplex cells into individual samples but cannot assign cells to specific donors, since the donor identities of the inferred genotypes are arbitrary. Depending on the method, there is also the option to improve performance by providing either external sample-specific genotypes, such as from matched bulk RNA sequencing, or a list of population SNPs, such as from the 1000 Genomes Project [21] for human samples.

Recently, genetic variation-based scRNA-seq demultiplexing tools have been applied to pooled samples from cancer cell lines [22,23], using known genotype references [5,22] and pools consisting of up to dozens of cell lines. However, systematic evaluations have not yet been performed in cancer for methods that do not require a genotype reference [3,4], and using pooled samples from the same cancer type from different individuals, which are likely to be more difficult to distinguish than cell lines from distinct cancer types. Cancer is characterized by widespread additional somatic mutations, including single nucleotide variants (SNVs) [24], as well as structural variation affecting the frequency of SNVs, which could interfere with the SNP signal used to distinguish individuals in this application of demultiplexing. The frequency of additional somatic SNVs, known as the tumor mutational burden (TMB), can vary widely between cancer types [25], as well as between patients and cancer subtypes [26,27]. However, the TMB is typically small relative to the overall population SNP burden [24]. For example, population SNPs with minor allele frequency (MAF) >1% are thought to occur on the order of once per 1000 nucleotides on average, or 1000 SNPs per Mb [28]. By contrast, high-TMB cancers have been defined as having around >10 or >20 additional mutations (SNVs) per Mb [26,27] -- approximately two orders of magnitude lower frequency than the population SNPs. In the case of typical scRNA-seq protocols that sequence the 3’ end of transcripts, only SNPs within the sequenced region (e.g. 100-200 nucleotides) can be detected, but the same arguments may be applied to compare the proportion of cancer SNVs against background SNPs. Therefore, it seems reasonable to expect that the natural genetic variation signal would not be severely obscured by the TMB, and that genetic variation-based demultiplexing tools should still perform well for pooled tissue samples from the same cancer type from different individuals. However, this assumption has not been rigorously tested. Due to the finite and

irreplaceable nature of tumor samples, we computationally evaluated demultiplexing algorithms to confirm that genetic variation-based demultiplexing performs adequately when applied to scRNA-seq pooling experimental designs in cancer, before committing samples to this experimental design strategy. In addition, we were interested in evaluating the degree to which these tools can reliably identify doublets consisting of cells from multiple individuals, including in experimental designs with extremely high proportions of doublets. Reliable doublet identification would allow the use of "super-loading" experimental designs, such as loading cells at very high concentration and subsequently removing identifiable doublets, providing substantial cost savings during library preparation [5,17,29]. In the future, these tools may also be well-suited for cell atlas initiatives, which are expected to cover large numbers of samples, including eventually those from cancer [30,31].

Here, we performed a benchmark evaluation of genetic variation-based demultiplexing in cancer scRNA-seq data using *in silico* simulations constructed from experimental scRNA-seq datasets with known sample identity for each cell. We evaluated two demultiplexing algorithms (Vireo [3] and demuxlet [5]) and five strategies for selecting the genotype reference list of SNPs used in the demultiplexing algorithms, including strategies that do not require a matched genotype reference. We also included varying proportions of simulated doublets by combining raw sequencing reads from multiple cell barcodes, which creates both identifiable doublets (from different individuals) and unidentifiable doublets (from the same individual). In addition, we tested performance for scenarios including a proportion of ambient RNA from simulated debris or lysed cells, by computationally assigning all reads from a percentage of cells to other randomly selected cells. In the benchmark evaluation, we considered scRNA-seq samples from two cancers that are potentially difficult to characterize: high-grade serous ovarian cancer (HGSOC) and lung adenocarcinoma. HGSOC is characterized by loss of TP53, and generally has medium to high SNV burden and high copy number variation (CNV) burden (particularly for focal copy number alterations), relative to other cancers [25,32], while lung adenocarcinoma is characterized by high SNV burden [25]. In addition, we compared against a baseline performance for healthy cells from cell lines [19]. Our results demonstrate that genetic

variation-based demultiplexing provides high recall at acceptable precision-recall tradeoffs in both high CNV and high SNV cancer types, even with extremely high simulated doublet proportions. However, high proportions of ambient RNA from debris can reduce performance. Our results demonstrate that these tools support experimental designs that incorporate sample pooling. We provide a reproducible Snakemake [33] workflow based on the best-performing combination of tools for estimating a genotype reference list of SNPs and demultiplexing samples identified in our benchmark, to facilitate experimental design efforts. The Snakemake workflow is modular, allowing users to substitute alternative tools. The workflow requires a set of scRNA-seq pilot samples, access to a Linux computing cluster, some familiarity with the Linux command line, and optionally matched bulk RNA-seq samples (for the highest demultiplexing performance and doublet identification). All code for the benchmark evaluation and Snakemake workflow is freely accessible at https://github.com/lmweber/snp-dmx-cancer.

Results

Genetic demultiplexing in HGSOC and lung adenocarcinoma

We evaluated the performance of genetic demultiplexing algorithms for scRNA-seq samples from HGSOC (high CNV) and lung adenocarcinoma (high SNV) using a set of benchmark evaluations and Snakemake [33] workflow built around freely available tools including Cell Ranger [34], samtools [35], bcftools [36], Unix string manipulation tools (sed and awk), cellSNP [37], and Vireo [3] (**Methods** and **Figure 1**). The HGSOC samples were collected at the Huntsman Cancer Institute, and the lung adenocarcinoma dataset is a published dataset sourced from [38]. **Table 1** provides a summary of the scRNA-seq cancer datasets. Additional details on data collection and accessibility are provided in **Methods**.

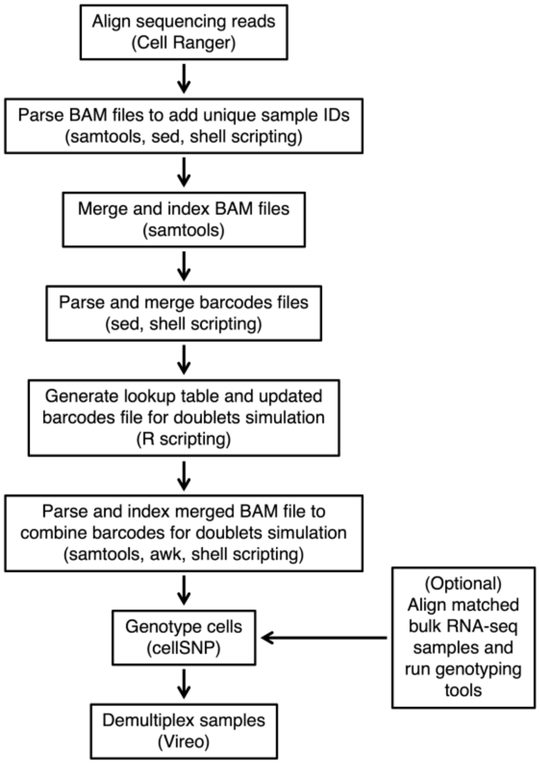

Figure 1. Schematic illustrating the steps in the Snakemake workflow. The workflow is designed to be modular, allowing users to substitute alternative tools. The Snakemake workflow runs a complete analysis for one dataset (HGSOC) and doublets simulation scenario (20% doublets). Our main benchmark evaluations include a second dataset (lung adenocarcinoma) and additional doublet simulation scenarios (30% doublets, no doublets). Additional and supplementary results include simulated proportions of ambient RNA from cell debris (10% and 20% debris), genotype references containing a subset of SNPs from a SNP array, and a healthy (non-cancer) cell line dataset. The optional step to run genotyping tools (e.g. on matched bulk RNA-seq samples) improved performance in our benchmark evaluations. Tools used in each step are shown in parentheses.

HGSOC dataset		Lung adenocarcinoma dataset	
Sample ID	Number of cells	Sample ID	Number of cells
X2	7,123	T08	4,093
X3	1,533	T09	4,267
X4	6,546	T20	4,521
		T25	4,428
		T28	5,789
		T31	7,069

Table 1. Summary of number of samples and number of cells per sample for scRNA-seq samples in HGSOC (GSE158937 and phs002262.v1.p1) and lung adenocarcinoma [38] (EGAD00001005054) datasets. The numbers of cells per sample listed are the numbers of cells provided by Cell Ranger [34] following sequencing read alignment. The HGSOC dataset additionally includes matched bulk RNA-seq samples for each sample. The lung adenocarcinoma dataset includes matched bulk whole exome sequencing samples (not used here) for each sample, but not matched bulk RNA-seq samples. Additional details for both datasets (as well as the healthy non-cancer cell line dataset from [19]) are provided in **Supplementary Table 2**.

High precision and recall performance using genetic demultiplexing

Using the HGSOC scRNA-seq and matched bulk RNA-seq data, we found the highest recall (defined as the proportion of true singlet cells for each sample that are identified as singlets and assigned to the correct sample) and best precision-recall tradeoff (where precision is defined as the proportion of identified cells for each sample that are true singlet cells from the correct sample), when using bcftools [36] to generate a genotype reference list of SNPs from the matched bulk RNA-seq samples, together with cellSNP/Vireo [3,37] for demultiplexing, in all simulation scenarios (no doublets, 20% doublets, or 30% doublets) (**Figure 2 a-c**). This scenario (labeled "bulkBcftools_cellSNPVireo" and colored light blue in **Figure 2**) achieves 99.0%, 99.9%, and 99.9% recall (values averaged across three scRNA-seq samples). However, in this scenario, the precision drops (100%, 85.9%, and 77.4%) (values averaged

across three scRNA-seq samples) as the percentage of doublets decreases with no doublets, 20% doublets, and 30% doublets (**Figure 2 a-c**, panels from left to right), respectively.

In general, we prefer higher recall at the expense of somewhat lower precision, so that we do not lose true singlet cells during the initial demultiplexing. If reduced precision is due to additional doublets that have been misclassified as singlets, these can potentially be identified and removed through downstream analyses, such as inspecting visualizations of unique molecular identifier (UMI) counts or detected genes per cell, or applying downstream doublet detection tools [6–10]. As an illustration, we investigated the types of incorrect calls leading to reduced precision in our top-performing scenario (cellSNP/Vireo with bulk RNA-seq reference) for the 30% doublets simulation in the HGSOC dataset (**Supplementary Table 3**). This showed that the doublet calls were relatively pure: of the cell barcodes called as doublets by Vireo, 99.2% were true identifiable doublets consisting of cells from distinct donors. Almost all the non-identifiable doublets (consisting of two cells from the same donor, which have the same germline SNPs) were assigned to the correct donor. By contrast, for demuxlet (HGSOC, 30% doublets, bulk RNA-seq reference), only 31.9% of the cell barcodes identified as doublets were true identifiable doublets (**Supplementary Table 4**), suggesting that cellSNP/Vireo can more reliably identify true doublets than demuxlet in these cancer samples (possibly due to non-standard allele fractions in cancer). We also applied a downstream doublet detection tool (scDblFinder [39]), but found that this did not perform well, returning large fractions of false positives and false negatives in both HGSOC and lung datasets (cellSNP/Vireo with bulk RNA-seq reference; 20% and 30% doublet scenarios) (**Supplementary Table 5**), suggesting that further analysis is required to reliably identify remaining doublets in cancer samples.

In the comparisons with demuxlet, we found that using bcftools [36] to generate a genotype reference list of SNPs from the matched bulk RNA-seq samples together with demuxlet (labeled “bulkBcftools_demuxlet” and colored green in **Figure 2**) resulted in somewhat higher precision (91.3%, 84.3%) with a large reduction to recall (53.0%, 52.1%) in the 20% and 30% doublet scenarios,

respectively (**Figure 2 b-c**). However, no further improvement in precision was observed (99.9%) with a large reduction in recall (52.8%) for the no doublets scenario (**Figure 2 a**).

In the scenarios where matched bulk RNA-seq samples are not available, the next best-performing scenarios were obtained using the genotype reference from the 1000 Genomes Project [21] (provided by the authors of cellSNP/Vireo) with no filtering of SNPs ("unfiltered") and genotype reference from the 1000 Genomes Project filtered to retain only SNPs in the 3' untranslated region (UTR) ("filtered", which speeds up runtime for 3'-tag sequencing protocols), together with cellSNP/Vireo for demultiplexing (labeled "1000GenomesUnfilt_cellSNPVireo" and "1000GenomesFilt_cellSNPVireo" and colored in orange and purple, respectively) (**Figure 2 a-c**). The "unfiltered" scenario achieved recall 97.9%, 99.0%, and 99.0% and precision 100%, 84.0%, and 74.5% with no doublets, 20% doublets, and 30% doublets respectively (**Figure 2 a-c**). Surprisingly, there is only a minor loss in performance in the "filtered" scenario, which achieved recall 94.3%, 95.0%, and 95.3% and precision 100%, 83.7%, and 73.8% respectively. Alternatively, when we evaluated the scenario to call SNPs directly from the scRNA-seq samples and use cellSNP/Vireo for demultiplexing (labeled "singlecellCellSNP_cellSNPVireo" and colored in dark blue), we found comparable recall (91.5%, 91.9%, and 92.1%) with a slight loss in precision (99.7%, 82.3%, and 72.7%) as the percentage of doublets decreases with no doublets, 20% doublets, and 30% doublets respectively (**Figure 2 a-c**).

Using the high-TMB lung adenocarcinoma scRNA-seq (without matched bulk RNA-seq) dataset, we only considered the scenario using the genotype reference from the 1000 Genomes Project (filtered) together with cellSNP/Vireo (labeled "1000GenomesFilt_cellSNPVireo" and colored in purple in **Figure 2**), as this resulted in the highest precision and recall in the HGSOC evaluation when using either the genotype reference from 1000 Genomes Project or directly calling SNPs from the scRNA-seq samples, while also keeping runtimes lower (details in **Figure 4**) than the 1000 Genomes (unfiltered) scenario. In this scenario (labeled "1000GenomesFilt_cellSNPVireo"), we found comparable ranges of precision and recall values as for the matching scenario in the HGSOC dataset (**Figure 2 d-f**). These results

demonstrate that we can also achieve excellent demultiplexing performance even in a higher-TMB cancer setting.

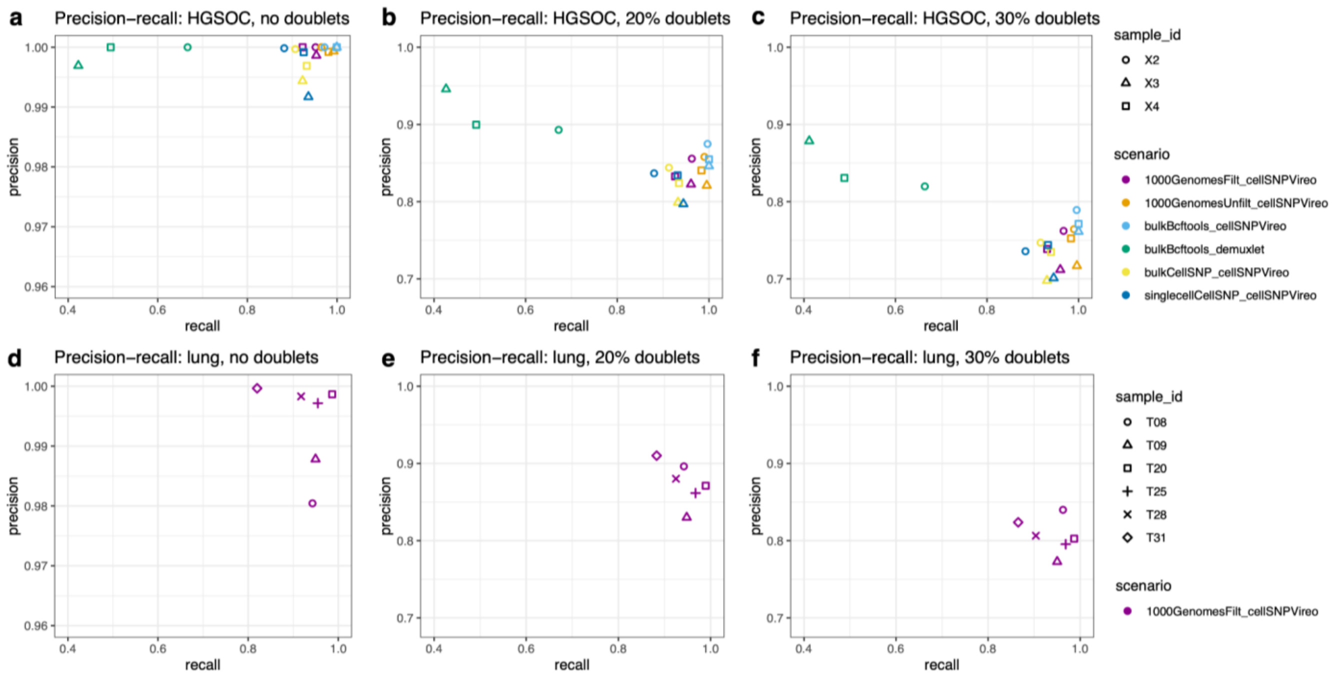

Figure 2. Performance evaluations for benchmark scenarios, for HGSOC dataset **(a-c)** and lung adenocarcinoma dataset **(d-f)**, across three proportions of simulated doublets (no doublets, 20% doublets, and 30% doublets). Performance is evaluated in terms of precision (y-axis) and recall (x-axis) for recovering the sample identities of true singlet cells from each scRNA-seq sample. Benchmark scenarios are labeled by color and with the naming scheme “genotypeMethod_demultiplexingMethod”. Samples within each dataset are identified with shapes. Note that y-axis limits (precision) for (a) and (d) differ from the other panels for improved visibility.

Reduced recall performance due to ambient RNA from simulated cell debris

Single-cell samples may contain proportions of ambient RNA from cell debris and lysed cells, with increased proportions in complex or necrotic samples [40], such as in cancer. Since SNPs from the ambient RNA may interfere with the set of SNPs observed for each droplet (cell barcode), this may

affect SNP-based demultiplexing performance. To evaluate this effect in cancer samples, we created additional simulations where either 10%, 20%, or 40% of final cell barcodes were assumed to represent debris or lysed cells, and assigned all sequencing reads from these cells randomly to other cell barcodes (**Figure 3** and **Supplementary Figures 1-2**). The range of debris proportions was selected to be in the higher range of previously published results for non-cancer cell line data [19]. For these simulations, we included the top-performing and computationally efficient scenarios from the main benchmark (cellSNP/Vireo with bulk RNA-seq and 1000 Genomes filtered references), as well as demuxlet for comparison (HGSOC dataset). These results showed a reduction in recall performance, although the reduction was smallest for the top-performing scenario ("bulkBcftools_cellSNPVireo", light blue). For the 10% debris simulations (**Figure 3**), we observed average recall of 85.1%, 86.2%, and 86.7% across samples (no doublets, 20% doublets, and 30% doublets scenarios) when using the bulk RNA-seq reference, and 61.7%, 60.3%, and 60.8% respectively when using the 1000 Genomes filtered reference, for the HGSOC dataset. For the lung dataset, recall decreased to an average of 57.0%, 54.2%, and 59.4% across samples (no doublets, 20% doublets, and 30% doublets) using the 1000 Genomes filtered reference. The performance of demuxlet dropped substantially, with average recall of 7.8%, 6.7%, and 6.4% across samples (no doublets, 20% doublets, and 30% doublets) using the bulk RNA-seq reference for the HGSOC dataset, suggesting that demuxlet is more sensitive to ambient RNA than cellSNP/Vireo. Additional results with higher proportions of simulated debris (20% or 40% of final cell barcodes, **Supplementary Figures 1-2**) showed greater reductions in recall performance. While precision performance was also somewhat reduced compared to the main results, the effect was much smaller than for recall (**Figure 3** and **Supplementary Figures 1-2**). Overall, these results demonstrate that ambient RNA reduces demultiplexing performance, although the effect is minimized when using the top-performing set of tools ("bulkBcftools_cellSNPVireo", i.e. cellSNP/Vireo with a bulk RNA-seq reference, when this is available).

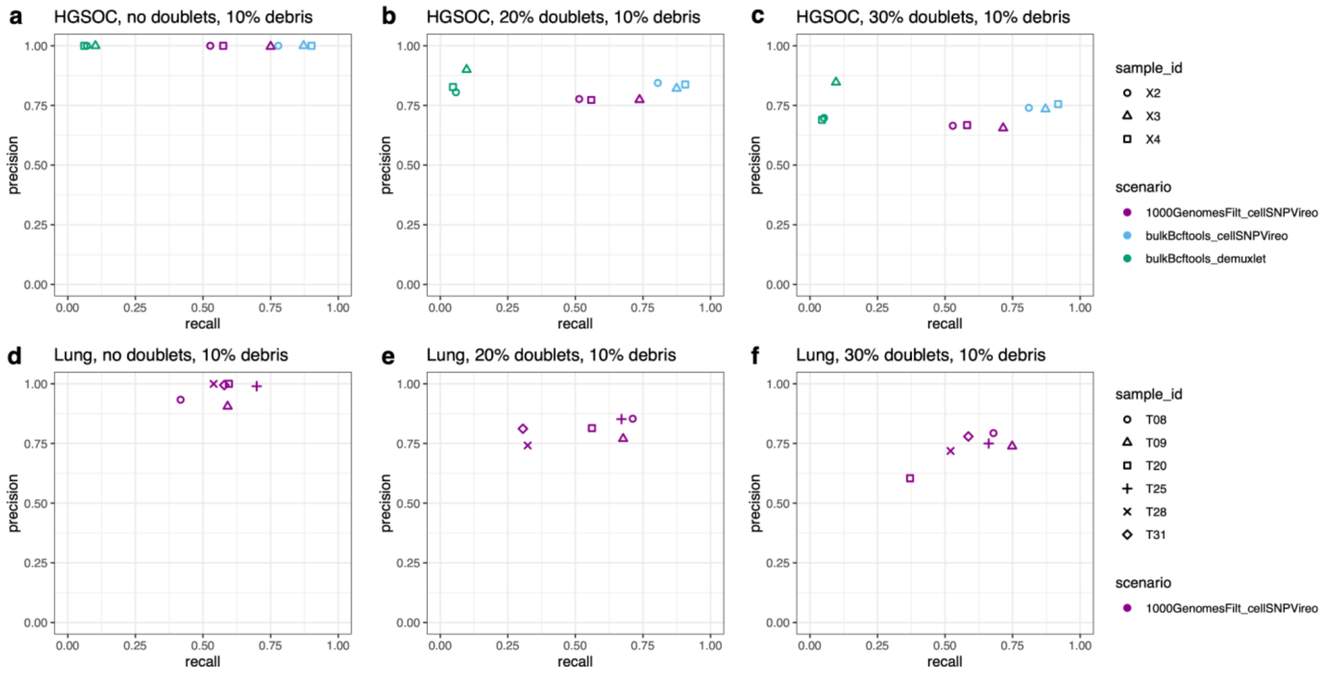

Figure 3. Performance evaluations for benchmark scenarios including ambient RNA from simulated cell debris. Top-performing and computationally efficient scenarios for HGSOC dataset **(a-c)** and lung adenocarcinoma dataset **(d-f)**, across three proportions of simulated doublets (no doublets, 20% doublets, 30% doublets), after introducing ambient RNA from simulated cell debris by assigning all reads from 10% of final cell barcodes to other randomly selected cell barcodes. Performance is evaluated in terms of precision (y-axis) and recall (x-axis) for recovering the sample identities of true singlet cells from each scRNA-seq sample. Benchmark scenarios are labeled by color and with the naming scheme “genotypeMethod_demultiplexingMethod”. Samples within each dataset are identified with shapes. Axis limits range from 0 to 1 for all panels.

Performance remains high when using subset of SNPs from SNP array

Since SNP arrays are often used for genotyping in cancer studies, we were also interested in demultiplexing performance when using SNPs from a SNP array, instead of the bulk RNA-seq or 1000 Genomes references used in the main results. To test expected performance when using SNP arrays, we generated additional simulations using a subset of SNPs from a common SNP array that also overlapped with our other genotype references. We selected the Infinium Multi-Ethnic Global-8 v1.0

array from the Multi-Ethnic Genotyping Array (MEGA) consortium, calculated the overlapping sets of SNPs from this array with either the 1000 Genomes filtered ("1000GenomesFiltMEGA"), 1000 Genomes unfiltered ("1000GenomesUnfiltMEGA"), or bulk RNA-seq ("bulkBcftoolsMEGA") reference (for the HGSOC dataset). This left either 16.5% of the 84,853 filtered 1000 Genomes SNPs, 8.6% of the 7,414,539 unfiltered 1000 Genomes SNPs, or 7.6% of the 605,367 bulk RNA-seq SNPs, or alternatively (compared to the array) 0.8%, 36.8%, or 2.6% of the original 1,733,345 array SNPs respectively (see **Supplementary Table 1** for a summary of the overlapping set sizes). Despite the large reduction in number of SNPs used for demultiplexing, performance for cellSNP/Vireo remained remarkably high, with almost no reduction in recall or precision performance in the top-performing scenario when using the overlapping SNPs from the bulk RNA-seq reference ("bulkBcftoolsMEGA") for the HGSOC dataset (average recall across samples 96.9%, 98,7%, and 99.0%, and average precision across samples 100%, 86.3%, and 77.7%, with no doublets, 20% doublets, and 30% doublets respectively). When using the 1000 Genomes filtered or 1000 Genomes unfiltered references ("1000GenomesFiltMEGA" or "1000GenomesUnfiltMEGA"), there was a somewhat larger reduction in recall. By contrast, the performance of demuxlet was substantially lower, suggesting that demuxlet is more sensitive to the set of SNPs used for demultiplexing (**Supplementary Figure 3**). Since the set of SNPs used in the top-performing scenario ("bulkBcftoolsMEGA_cellSNPVireo") is much smaller than the full set of SNPs from the array, and performance is only slightly reduced, these results suggest that demultiplexing performance with cellSNP/Vireo is likely to remain high when using the full array.

High performance in baseline comparison for non-cancer cell lines

As a baseline comparison for healthy (non-cancer) data, we evaluated performance in a dataset consisting of 5 samples of induced pluripotent stem cell (iPSC) cell lines from the Human iPSC Initiative (HipSci), which was previously published by [19]. This dataset contained an average of around 9,000 cells per sample, with relatively high unique molecular identifier (UMI) counts per cell (**Supplementary**

Table 2). We generated simulation scenarios containing no doublets, 20% doublets, and 30% doublets, and evaluated demultiplexing performance using cellSNP/Vireo with the 1000 Genomes 3' UTRs filtered genotype reference. We observed similar demultiplexing performance in terms of precision and recall as in the main results for the corresponding scenarios (**Supplementary Figure 4**). These results provide a baseline comparison confirming that these demultiplexing tools perform well in non-cancer data, which is consistent with previous published results [3,19], as well as a confirmation that our simulation framework can be successfully applied in both cancer and non-cancer settings.

Computational runtime of genetic demultiplexing workflow steps and genotyping tools

We evaluated the computational runtimes for the various components in our benchmark scenarios and Snakemake workflow using the HGSOC data. First, we found the computational runtimes for the various steps in the genetic demultiplexing workflow vary across multiple orders of magnitude and depended on whether the tool could be parallelized. The parallelizable tools (Cell Ranger and cellSNP) were run using 10 processor cores to decrease runtime, while the remaining tools used a single core. All evaluations of runtimes were performed on a high-performance Linux computing cluster. In the Snakemake workflow (**Figure 4 a**), the slowest steps were running Cell Ranger (approximately 6 hours per sample using 10 cores) and parsing the merged BAM file containing aligned reads to combine cell barcodes into simulated doublets (approximately 1 day). For the cellSNP step in the workflow, runtime depended on the choice of genotype reference list of SNPs (**Figure 4 b**). In particular, filtering the genotype reference from the 1000 Genomes Project [21] (provided by the authors of cellSNP/Vireo) to retain only SNPs in the 3' UTRs reduced runtime from approximately 2.5 hours to less than 10 minutes ("1000GenomesUnfilt_cellSNP" vs. "1000GenomesFilt_cellSNP"), at the cost of only a small drop in performance (**Figure 2**). The runtime shown for the cellSNP step in **Figure 4 a** corresponds to the highest-performing scenario from **Figure 2** ("bulkBcftools_cellSNP").

We also evaluated computational runtimes for the genotyping tools used to generate the genotype reference lists of SNPs from either the matched bulk RNA-seq samples or directly from the scRNA-seq samples (**Figure 4 c**). Here, we found by far the slowest option was to use cellSNP to generate the genotype reference directly from the scRNA-seq samples (between 1 and 4.5 days per sample using 10 cores), while generating the genotype reference from the bulk RNA-seq samples took either approximately 2 minutes per sample using cellSNP (10 cores) or 5 hours using bcftools.

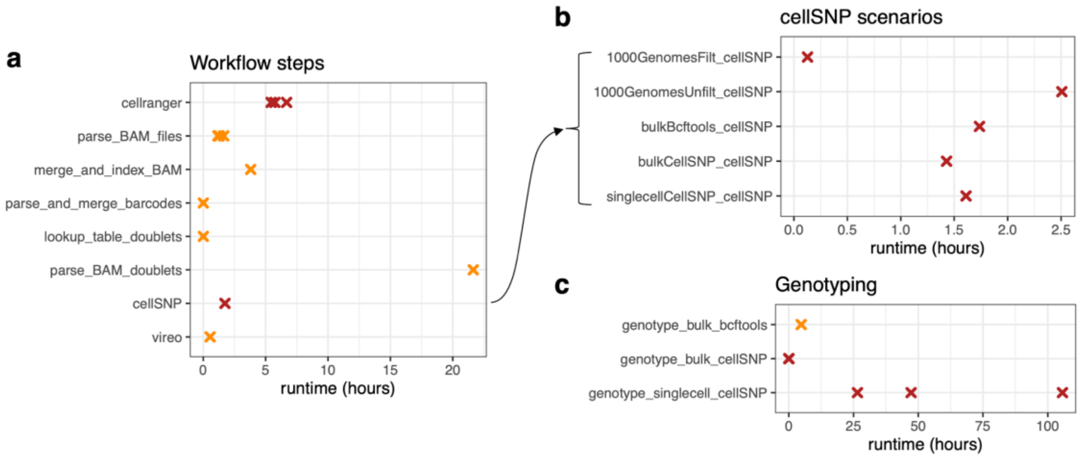

Figure 4. Computational runtimes (in hours) of genetic demultiplexing workflow steps and genotyping tools. **(a)** Runtimes for steps in the complete Snakemake workflow, for a single dataset (HGSOC) and doublets simulation scenario (20%). Parallelized tools (Cell Ranger and cellSNP; points indicated in dark red) were run using 10 processor cores, and all other tools using a single core (points indicated in orange), on a high-performance Linux computing cluster. For steps where samples were processed individually, separate points are shown for each sample. **(b)** Runtimes for alternative options for running cellSNP in the workflow, depending on the choice of genotype reference (1000 Genomes filtered, 1000 Genomes unfiltered, matched bulk RNA-seq using bcftools, matched bulk RNA-seq using cellSNP, and single-cell RNA-seq using cellSNP). The cellSNP step in (a) matches the row “bulkBcftools_cellSNP” in (b), which was the highest-performing scenario from Figure 2. **(c)** Runtimes for alternative options to generate genotype reference file. Horizontal axis scales differ between panels for improved visibility.

Discussion

Pooled single-cell experimental designs before library preparation together with genetic variation-based computational sample demultiplexing are a convenient and effective strategy for reducing library preparation costs and potential batch effects in scRNA-seq studies. Compared to barcoding-based approaches such as MULTI-seq [16] and cell hashing [17], demultiplexing performance may be lower depending on the quality of the genotype reference used, however genetic variation-based methods provide significant advantages in terms of simpler sample preparation and cost savings during library preparation. Here, we performed an *in silico* benchmark evaluation based on real scRNA-seq tumor tissue datasets to confirm that these tools can be effectively applied to pooled cancer samples from different individuals. We compared two demultiplexing tools (Vireo [3] and demuxlet [5]) and five genotype references. We selected HGSOC and lung adenocarcinoma, two cancer types characterized by a relatively high TMB. Previous benchmark evaluations [3,4,19] have only evaluated these tools in non-cancer datasets, which are not affected by additional mutational SNV burden that could potentially obscure the natural genetic variation SNP signal used to distinguish individuals, while previous evaluations in cancer [22,23] have relied on matched genotype references and focused on cancer cell lines, which are likely to be easier to distinguish than samples of the same cancer type from different individuals. Our benchmark evaluations include high proportions of simulated doublets (up to 30%), confirming that these tools can be used to identify singlet cells in "super-loading" experimental designs to achieve considerable cost savings in library preparation [5,17,29]. Additional analyses showed that performance remained high when using subsets of SNPs from a SNP array for the genotype reference, as well as in a baseline comparison using a healthy (non-cancer) cell line dataset [19]. However, introducing proportions of ambient RNA from simulated cell debris or lysed cells reduced performance in terms of recall, although this effect was minimized when using the matched bulk RNA-seq genotype reference. This suggests that, in cancer samples with significant proportions of ambient RNA, e.g. from cell debris due to necrosis, it may be important to consider applying experimental techniques such as

straining to remove cell debris. As an illustration of expected cost savings due to lower library preparation costs in a multiplexed experimental design, we estimated library preparation and sequencing costs for designs with 4 to 8 samples, using the "Cost Per Cell" online calculator provided by the Satija Lab [29] (**Supplementary Figure 4**). We assumed 4,000 desired cells per sample after demultiplexing, i.e. after discarding identifiable doublets consisting of cells from multiple samples, but including the smaller number of non-identifiable doublets (multiple cells from the same sample, which have the same SNP profiles and cannot be distinguished using genetic demultiplexing). These designs result in cost savings of approximately 60% of the estimated cost for the experiment when using full multiplexing (all samples prepared as a single library and sequenced together) compared to no multiplexing (**Supplementary Figure 4**).

In our HGSOC dataset, we achieved the best demultiplexing performance (and relatively efficient runtimes) when using matched bulk RNA-seq samples to generate a genotype reference list of SNPs using bcftools [36], together with cellSNP/Vireo [3,37] for demultiplexing. However, using a standard list of population SNPs from the 1000 Genomes Project [21] (which does not require matched bulk RNA-seq samples) provided by the authors of cellSNP/Vireo also achieved good performance. In this case, filtering the population SNPs to retain only SNPs in the 3' UTRs significantly reduced runtime, at the cost of only slightly lower demultiplexing performance. For the lung adenocarcinoma dataset, performance was comparable to the matching scenario in the HGSOC dataset, confirming that performance was not seriously affected by the higher TMB, and that genetic demultiplexing can be effectively applied in this setting. Since most other cancer types have lower TMB [25], we expect these results to apply to most cancer types. We provide a freely available, modular Snakemake [33] workflow implementing the best-performing scenario from our benchmark, built around cellSNP/Vireo [3,37] and other freely accessible tools, as well as additional R and shell scripts to reproduce all analyses in our benchmark evaluations and additional analyses (https://github.com/lmweber/snp-dmx-cancer), to allow other researchers to perform similar analyses for experimental design, planning, and budgeting purposes in their own datasets.

Our study has several limitations. While the best-performing benchmark scenario achieves excellent recall, precision is somewhat lower. While most doublet calls from cellSNP/Vireo in this scenario were true identifiable doublets, additional true identifiable doublets were incorrectly called as singlets, reducing precision for each demultiplexed sample. Although downstream doublet detection tools [6–10] could be applied to remove any remaining doublets, we found that this did not perform well in an initial analysis. Further work could consider a systematic evaluation of downstream doublet detection tools in the context of cancer, to complement previous results in non-cancer data [41]. In this study, we have built our Snakemake workflow around the best-performing tools (cellSNP/Vireo [3,37] for demultiplexing and using matched bulk RNA-seq samples for genotyping) and compared against demuxlet [5] and baseline scenarios (no doublets), but we have not performed a comprehensive benchmark evaluation of all available tools, such as additional tools for demultiplexing (e.g. scSplit [4], souporcell [19], and freemuxlet [20]). However, we have implemented the Snakemake workflow to be modular, so that other users may substitute alternative tools if they prefer. We also investigated the use of salmon alevin [42] for pseudoalignment of scRNA-seq reads (instead of Cell Ranger), but found that this was not compatible with the demultiplexing tools since pseudoalignment occurs at the transcriptomic instead of genomic level. However, future developments may enable conversion between transcriptomic and genomic aligned reads, and we have included alternative code scripts for salmon alevin within our code repository. Our evaluations considered only two tumor types (HGSOC and lung adenocarcinoma), and performance may differ for other cancer types or tissues. However, since we were able to demonstrate good performance in lung adenocarcinoma, one of the highest TMB cancers, we anticipate these results will also be applicable for other cancer types, which will generally have lower TMB. For the lung adenocarcinoma dataset, matched bulk whole exome sequencing data were also available for these six samples, which could be used to further improve performance using additional genotyping tools. Future work could also consider generating additional experimental data to further benchmark these tools, instead of relying on *in silico* evaluations, although in this case it may be difficult to generate a reliable ground truth.

More fundamentally, due to the reliance on genetically distinct SNP profiles, genetic demultiplexing tools work well for human samples from unrelated individuals, but performance is expected to decrease for genetically similar samples such as hereditary related human populations [19] or inbred mice, and these methods are not applicable to samples from the same individual [3]. Without a sample-specific genotype reference, it is also not possible to assign cells to specific donors, since the donor identities of inferred genotypes are arbitrary. Similarly, genetic demultiplexing does not allow identifying doublets consisting of cells from the same individual, although these are only a subset of total doublets, and decrease as a proportion of total doublets with increasing number of multiplexed samples. We also have not considered the question of identifying doublets consisting of distinct cell types (from either the same or different individuals), which may be identified using downstream analysis tools. For some experiments, a useful design strategy may also be to combine genetic-based and barcoding-based multiplexing, e.g. multiple treatments on samples from the same individual. Our Snakemake workflow can be used to demultiplex up to around 12 pooled samples without a genotype reference (limited by the demultiplexing algorithm Vireo) -- beyond this, the demultiplexing performance of the Vireo algorithm has been shown to decrease [3]. For larger experiments, if matched bulk RNA-seq samples are not available, multiple sample pools could be used, with demultiplexing done separately for each pool [3]. Splitting an experiment across multiple pools and demultiplexing within each pool also represents an opportunity to implement improved experimental designs to reduce batch effects and confounding. Finally, the Snakemake workflow is relatively computationally intensive, and requires access to a high-performance Linux computing cluster or server.

Methods

Benchmark evaluations and workflow

We begin by describing in detail our benchmark evaluation framework, and note that our additional Snakemake [33] workflow is built around the combination of tools that resulted in the best performance from the benchmark evaluation. Specifically, the benchmark and workflow make use of several freely available tools, including Cell Ranger [34], samtools [35], bcftools [36], Unix string manipulation tools (sed and awk), cellSNP [37], and Vireo [3]. The Snakemake workflow is designed to be modular, allowing other alternative or new tools to be substituted. All code for the benchmark evaluation and Snakemake workflow is freely available at https://github.com/lmweber/snp-dmx-cancer.

In our benchmark evaluation, we considered two genetic demultiplexing algorithms: (i) Vireo [3] together with cellSNP [37], and (ii) demuxlet [5] as an alternative genetic-based demultiplexing tool. We evaluated five scenarios for obtaining the genotype reference list of SNPs used in the demultiplexing algorithm: (i) list of population SNPs from the 1000 Genomes Project [21] provided by the authors of cellSNP/Vireo; (ii) list of population SNPs from the 1000 Genomes Project with an additional filtering step to retain only SNPs in the 3' untranslated regions (UTRs) for faster runtime (this strategy is appropriate for 3'-tag sequencing protocols, but could also be adapted for 5'-tag or full-transcript sequencing); (iii) sample genotyping from matched bulk RNA-seq samples using bcftools [36]; (iv) sample genotyping from matched bulk RNA-seq samples using cellSNP [37]; and (v) sample genotyping from scRNA-seq samples using cellSNP [37]. Scenario (ii) was used for both datasets (HGSOC and lung adenocarcinoma), and the remaining scenarios were applied to the HGSOC dataset only. Scenarios (iii) and (iv) require matched bulk RNA-seq samples, while scenarios (i) and (v) have slow runtimes. Specifically, for the HGSOC dataset, we evaluated performance across several combinations of methods for genotyping and demultiplexing (labeled as "genotypeMethod_demultiplexingMethod" in Results). For the lung adenocarcinoma dataset, we used

the list of population SNPs from the 1000 Genomes Project provided by the authors of cellSNP/Vireo, filtered to retain only SNPs in the 3' UTRs.

For the main benchmark evaluations, we used two cancer datasets. The first dataset consists of three unique molecular identifier (UMI)-based scRNA-seq HGSOC samples measured on the 10x Genomics platform [43], obtained from separate, unrelated individuals at the Huntsman Cancer Institute at the University of Utah. We also obtained matched bulk RNA-seq samples from the same three individuals for sample genotyping. The raw data is available by controlled access via the Database of Genotypes and Phenotypes (dbGaP) (phs002262.v1.p1), and processed gene count tables are available from the Gene Expression Omnibus (GEO) (GSE158937). The second dataset consists of six UMI-based scRNA-seq higher-TMB lung adenocarcinoma samples measured on the 10x Genomics platform, previously published by [38]. Raw data for all samples in this study are available by controlled access from the European Genome-phenome Archive (EGA) (EGAD00001005054). For our study, we used six samples identified as having TMB >25 mutations / Mb (see [38], Figure 2d and Methods). **Table 1** and **Supplementary Table 2** provide a summary of the scRNA-seq datasets.

Performance was evaluated in terms of precision and recall for demultiplexing each scRNA-seq sample. We also recorded computational runtime for each step in the workflow and benchmark scenarios. Recall is defined as the proportion of true singlet cells for each sample that are identified as singlets and assigned to the correct sample. Precision is defined as the proportion of identified cells for each sample that are true singlet cells from the correct sample. Runtime was evaluated using the Unix `date` command. We used R version 4.1 for random number generation and evaluation steps performed in R, and created figures using ggplot2 [44].

For our benchmark evaluation, we developed three *in silico* simulation scenarios for each dataset -- containing either no doublets, 20% simulated doublets, or 30% simulated doublets. Doublets were simulated by combining cell barcode labels from random sets of two cells in the raw sequencing reads

mapped using Cell Ranger [34], so that either 20% or 30% of the final barcodes represent doublets. For example, starting with 15,202 original cells in the HGSOC dataset, 3,508 randomly selected cells were combined with 3,508 other cells to create simulated doublets, leaving 11,694 final cell barcodes, of which 3,508 (30%) represent doublets. The 30% doublets scenario represents the upper end of our planned strategy for a “super-loading” experimental design, i.e. loading multiplexed cells at extremely high concentration to reduce library preparation costs and subsequently removing identifiable doublets [5,17,29]; the 20% doublets scenario represents an intermediate super-loading scenario; and the no doublets scenario serves as a best-case baseline scenario to evaluate performance of the demultiplexing tools.

Ambient RNA from simulated cell debris

For the scenarios containing ambient RNA from simulated cell debris or lysed cells, we selected a percentage of cell barcodes (10%, 20%, or 40%) after doublet creation, and assigned all sequencing reads from these cell barcodes to other randomly selected cell barcodes, in each of the no doublets, 20% doublets, and 30% doublets scenarios. The debris percentages (10%, 20%, and 40%) were selected to be in the higher range of previously published results for non-cancer data [19], since ambient RNA is expected to be relatively abundant in complex or necrotic samples [40] such as cancer.

Subset of SNPs from SNP array

For the simulated SNP array analyses, we selected a widely used SNP array (Infinium Multi-Ethnic Global-8 v1.0 array from the Multi-Ethnic Genotyping Array Consortium (MEGA) Consortium), and calculated the overlapping sets of SNPs between the total 1.7 million SNPs from the array and our existing genotype references (see **Supplementary Table 1** for a summary of the overlapping set sizes). Then, we re-ran our benchmark evaluations using the subsets of SNPs from the overlaps as the genotype references.

Healthy (non-cancer) cell line data

For a baseline comparison with healthy (non-cancer) data, we combined sequencing reads from 5 samples of induced pluripotent stem cell (iPSC) cell lines from the Human iPSC Initiative (HipSci), which were previously published by [19], and added the same percentages of doublets (no doublets, 20% doublets, or 30% doublets) as in our main analyses. The raw data are available from the European Nucleotide Archive (ENA) (ERS2630502-ERS2630506). Compared to our cancer samples, these samples contained relatively higher numbers of cells per sample, as well as higher unique molecular identifier (UMI) counts per cell (details are provided in **Supplementary Table 2**).

Single-cell RNA sequencing of ovarian tumors

De-identified HGSOC samples were processed after cryopreservation in liquid nitrogen where tissue chunks were stored in RPMI media with 10% fetal bovine serum and 10% DMSO. Samples were thawed and dissociated to single cells using the Miltenyi Human Tumor Dissociation Kit and the GentleMACS dissociator. Samples were incubated on the GentleMACS at 37°C for 1 hour with the setting of 1,865 rounds per run. A 70 µm MACS smart strainer was used to deplete cell doublets before loading onto the 10x Genomics Chromium Controller. Library preparation was performed using the 10x Genomics 3’ Gene Expression Library Prep v3 and libraries were sequenced on an Illumina NovaSeq instrument.

Availability of source code and requirements

Project name: snp-dmx-cancer

Project home page: https://github.com/lmweber/snp-dmx-cancer

Operating system: Linux

Programming language: Shell, R

Other requirements: High performance computing (HPC) cluster with Sun Grid Engine (SGE) scheduler

License: MIT

Code availability

All code, scripts to reproduce the benchmark evaluations, supplementary analyses, generate figures in the manuscript, and run the Snakemake workflow are freely accessible from GitHub [45]. An archival copy of our GitHub repository is available via the *GigaScience* database GigaDB [46]. All tools used within the benchmark evaluations and workflow are freely available, as described in Methods. Software versions used were Cell Ranger 4.0.0, bcftools 1.10.2-91-g365d117, demuxlet 3ab507c, cellsnp-lite 1.2.0, and Vireo 0.5.0.

Data availability

Raw and processed sequencing data generated in this study (HGSOC dataset) are available from the Database of Genotypes and Phenotypes (dbGaP) (raw data consisting of FASTQ files, accession phs002262.v1.p1) and Gene Expression Omnibus (GEO) (processed data files containing gene count tables, accession GSE158937). The lung adenocarcinoma dataset was previously published by [36], and is available from the European Genome-phenome Archive (EGA) (EGAD00001005054). The healthy (non-cancer) iPSC cell line data were previously published by [19], and are available from the European Nucleotide Archive (ENA) (ERS2630502-ERS2630506).

Acknowledgments

We thank Yuanhua Huang for assistance with running Vireo and cellSNP; Davis McCarthy for advice regarding Vireo; and attendees from the Stephanie Hicks and Kasper Hansen joint lab meetings at Johns Hopkins University for helpful feedback and discussions. We thank Hae-Ock Lee and Myung-Ju Ahn of the Samsung Medical Center for providing access to the lung adenocarcinoma dataset. Research reported in this publication utilized the Biorepository and Molecular Pathology Shared Resource and the High-Throughput Genomics Shared Resource at the Huntsman Cancer Institute at University of Utah and was supported by NIH/NCI award P30 CA042014. Computational analyses were performed using the Joint High Performance Computing Exchange (JHPCE) high-performance computing facility in the Department of Biostatistics at the Johns Hopkins Bloomberg School of Public Health. The content is solely the responsibility of the authors and does not necessarily represent the official views of the NIH.

Author contributions

LMW: Software, Formal analysis, Investigation, Data curation, Writing - Original Draft, Visualization
AAH: Software
PFH: Software, Writing - Review & Editing
KCB: Investigation
JG: Investigation, Resources, Writing - Review & Editing
JAD: Resources, Writing - Review & Editing, Funding acquisition
CSG: Conceptualization, Resources, Writing - Review & Editing, Funding acquisition
SCH: Conceptualization, Resources, Writing - Original Draft, Writing - Review & Editing, Supervision, Funding acquisition

Ethics approval and consent to participate

Ovarian cancer tissue was obtained and studied under written informed consent at the Huntsman Cancer Institute through approved University of Utah Institutional Review Board protocols IRB_00010924 and IRB_00118086. Analysis of human data in this study was also approved by the University of Pennsylvania Institutional Review Board (IRB protocol 832353) and the Johns Hopkins Bloomberg School of Public Health Institutional Review Board (IRB00013099).

Competing interests

The authors declare no conflicts of interest.

Funding

LMW, AHA, KCB, JG, JAD, CSG, and SCH were supported by the National Institutes of Health grant from the National Cancer Institute R01CA237170. JAD is also supported by Huntsman Cancer Foundation and National Institutes of Health grant from the National Cancer Institute P30 CA042014 (to N. Ulrich).

References

1. Hicks SC, Townes FW, Teng M, Irizarry RA. Missing data and technical variability in single-cell RNA-sequencing experiments. Biostatistics. 2018;19: 562–578.
2. Tung P-Y, Blischak JD, Hsiao CJ, Knowles DA, Burnett JE, Pritchard JK, et al. Batch effects and the effective design of single-cell gene expression studies. Scientific Reports. 2017;7: 39921.
3. Huang Y, McCarthy DJ, Stegle O. Vireo: Bayesian demultiplexing of pooled single-cell RNA-seq data without genotype reference. Genome Biology. 2019;20: 273.
4. Xu J, Falconer C, Nguyen Q, Crawford J, McKinnon BD, Mortlock S, et al. Genotype-free demultiplexing of pooled single-cell RNA-seq. Genome Biology. 2019;20: 290.
5. Kang HM, Subramaniam M, Targ S, Nguyen M, Maliskova L, McCarthy E, et al. Multiplexed droplet single-cell RNA-sequencing using natural genetic variation. Nature Biotechnology. 2018;36: 89–94.
6. Germain P-L, Sonrel A, Robinson MD. pipeComp, a general framework for the evaluation of computational pipelines, reveals performant single cell RNA-seq preprocessing tools. Genome Biology. 2020;21.
7. Bais AS, Kostka D. scds: computational annotation of doublets in single-cell RNA sequencing data. Bioinformatics. 2019;36: 1150–1158.
8. Wolock SL, Lopez R, Klein AM. Scrublet: Computational Identification of Cell Doublets in Single-Cell Transcriptomic Data. Cell Systems. 2019;8: 281–291.e9.
9. McGinnis CS, Murrow LM, Gartner ZJ. DoubletFinder: Doublet Detection in Single-Cell RNA Sequencing Data Using Artificial Nearest Neighbors. Cell Systems. 2019;8: 329–337.e4.
10. Lun ATL, McCarthy DJ, Marioni JC. A step-by-step workflow for low-level analysis of single-cell RNA-seq data with Bioconductor. F1000Research. 2016;5: 2122.

11. Farouni R, Djambazian H, Ferri LE, Ragoussis J, Najafabadi HS. Model-based analysis of sample index hopping reveals its widespread artifacts in multiplexed single-cell RNA-sequencing. Nature Communications. 2020;11: 2704.

12. Griffiths JA, Richard AC, Bach K, Lun ATL, Marioni JC. Detection and removal of barcode swapping in single-cell RNA-seq data. Nature Communications. 2018;9: 2667.

13. Costello M, Fleharty M, Abreu J, Farjoun Y, Ferriera S, Holmes L, et al. Characterization and remediation of sample index swaps by non-redundant dual indexing on massively parallel sequencing platforms. BMC Genomics. 2018;19: 332.

14. Sinha R, Stanley G, Gulati GS, Ezran C, Travaglini KJ. Index switching causes "spreading-of-signal" among multiplexed samples in Illumina HiSeq 4000 DNA sequencing. bioRxiv (https://www.biorxiv.org/content/101101/125724v1) (doi: https://doi.org/101101/125724). 2017.

15. Illumina. Effects of index misassignment on multiplexing and downstream analysis. White Paper (https://www.illumina.com/content/dam/illumina-marketing/documents/products/whitepapers/index-hopping-white-paper-770-2017-004.pdf). 2018.

16. McGinnis CS, Patterson DM, Winkler J, Conrad DN, Hein MY, Srivastava V, et al. MULTI-seq: sample multiplexing for single-cell RNA sequencing using lipid-tagged indices. Nature Methods. 2019;16: 619–626.

17. Stoeckius M, Zheng S, Houck-Loomis B, Hao S, Yeung BZ, Mauck WM 3rd, et al. Cell Hashing with barcoded antibodies enables multiplexing and doublet detection for single cell genomics. Genome Biology. 2018;19: 224.

18. Xin H, Lian Q, Jiang Y, Luo J, Wang X, Erb C, et al. GMM-Demux: sample demultiplexing, multiplet detection, experiment planning, and novel cell-type verification in single cell sequencing. Genome Biology. 2020;21.

19. Heaton H, Talman AM, Knights A, Imaz M, Gaffney DJ, Durbin R, et al. Souporcell: robust

clustering of single-cell RNA-seq data by genotype without reference genotypes. Nature Methods. 2020;17: 615–620.

20. Zhang F, Kang HM. popscle: A suite of population scale analysis tools for single-cell genomics data (freemuxlet). Software package (https://github.com/statgen/popscle). 2020.

21. 1000 Genomes Project Consortium, Auton A, Brooks LD, Durbin RM, Garrison EP, Kang HM, et al. A global reference for human genetic variation. Nature. 2015;526: 68–74.

22. McFarland JM, Paolella BR, Warren A, Geiger-Schuller K, Shibue T, Rothberg M, et al. Multiplexed single-cell transcriptional response profiling to define cancer vulnerabilities and therapeutic mechanism of action. Nature Communications. 2020;11: 4296.

23. Kinker GS, Greenwald AC, Tal R, Orlova Z, Cuoco MS, McFarland JM, et al. Pan-cancer single-cell RNA-seq identifies recurring programs of cellular heterogeneity. Nature Genetics. 2020;52: 1208–1218.

24. Spencer DH, Zhang B, Pfeifer J. Chapter 8 - Single Nucleotide Variant Detection Using Next Generation Sequencing. In: Kulkarni S, Pfeifer J, editors. Clinical Genomics. 2015. pp. 109–127.

25. ICGC/TCGA Pan-Cancer Analysis of Whole Genomes Consortium. Pan-cancer analysis of whole genomes. Nature. 2020;578: 82–93.

26. Fancello L, Gandini S, Pelicci PG, Mazzarella L. Tumor mutational burden quantification from targeted gene panels: major advancements and challenges. Journal for Immunotherapy of Cancer. 2019;7: 183.

27. Chalmers ZR, Connelly CF, Fabrizio D, Gay L, Ali SM, Ennis R, et al. Analysis of 100,000 human cancer genomes reveals the landscape of tumor mutational burden. Genome Medicine. 2017;9: 34.

28. Sherry ST, Ward MH, Kholodov M, Baker J, Phan L, Smigielski EM, et al. dbSNP: the NCBI

database of genetic variation. Nucleic Acids Research. 2001;29: 308–311.

29. Hafemeister C, Satija R. Cost Per Cell: Multiplexing cost calculator. Website (https://satijalab.org/costpercell). 2018.

30. Regev A, Teichmann SA, Lander ES, Amit I, Benoist C, Birney E, et al. The Human Cell Atlas. eLife. 2017;6: 1–30.

31. Taylor DM, Aronow BJ, Tan K, Bernt K, Salomonis N, Greene CS, et al. The Pediatric Cell Atlas: Defining the Growth Phase of Human Development at Single-Cell Resolution. Developmental Cell. 2019;49: 10–29.

32. Ciriello G, Miller ML, Aksoy BA, Senbabaoglu Y, Schultz N, Sander C. Emerging landscape of oncogenic signatures across human cancers. Nature Genetics. 2013;45: 1127–1133.

33. Köster J, Rahmann S. Snakemake — a scalable bioinformatics workflow engine. Bioinformatics. 2012;28: 2520–2522.

34. 10x Genomics. Cell Ranger. Software (https://support10xgenomics.com/single-cell-gene-expression/software/overview/welcome). 2020.

35. Li H, Handsaker B, Wysoker A, Fennell T, Ruan J, Homer N, et al. The Sequence Alignment/Map format and SAMtools. Bioinformatics. 2009;25: 2078–2079.

36. Li H. A statistical framework for SNP calling, mutation discovery, association mapping and population genetical parameter estimation from sequencing data. Bioinformatics. 2011;27: 2987–2993.

37. Huang Y. cellSNP. Software package (https://github.com/single-cell-genetics/cellSNP). 2020.

38. Kim N, Kim HK, Lee K, Hong Y, Cho JH, Choi JW, et al. Single-cell RNA sequencing demonstrates the molecular and cellular reprogramming of metastatic lung adenocarcinoma. Nature Communications. 2020;11: 2285.

39. Germain P-L. scDblFinder. R package, version 160. 2021.

40. Young MD, Behjati S. SoupX removes ambient RNA contamination from droplet-based single-cell RNA sequencing data. GigaScience. 2020;9.

41. Xi NM, Li JJ. Benchmarking Computational Doublet-Detection Methods for Single-Cell RNA Sequencing Data. Cell Systems. 2021;12: 176–194.e6.

42. Srivastava A, Malik L, Smith T, Sudbery I, Patro R. Alevin efficiently estimates accurate gene abundances from dscRNA-seq data. Genome Biology. 2019;20: 65.

43. Zheng GXY, Terry JM, Belgrader P, Ryvkin P, Bent ZW, Wilson R, et al. Massively parallel digital transcriptional profiling of single cells. Nature Communications. 2017;8: 14049.

44. Wickham H. ggplot2: Elegant Graphics for Data Analysis. Springer-Verlag New York; 2016.

45. Weber L. snp-dmx-cancer. GitHub repository; accessed 26 Aug. 2021. https://github.com/lmweber/snp-dmx-cancer.

46. Weber LM, Hippen AA, Hickey PF, Berrett KC, Gertz J, Doherty JA, Greene CS, Hicks SC Supporting data for "Genetic demultiplexing of pooled single-cell RNA-sequencing samples in cancer facilitates effective experimental design." GigaScience Database. 2021. http://dx.doi.org/10.5524/100921

Supplementary Figures

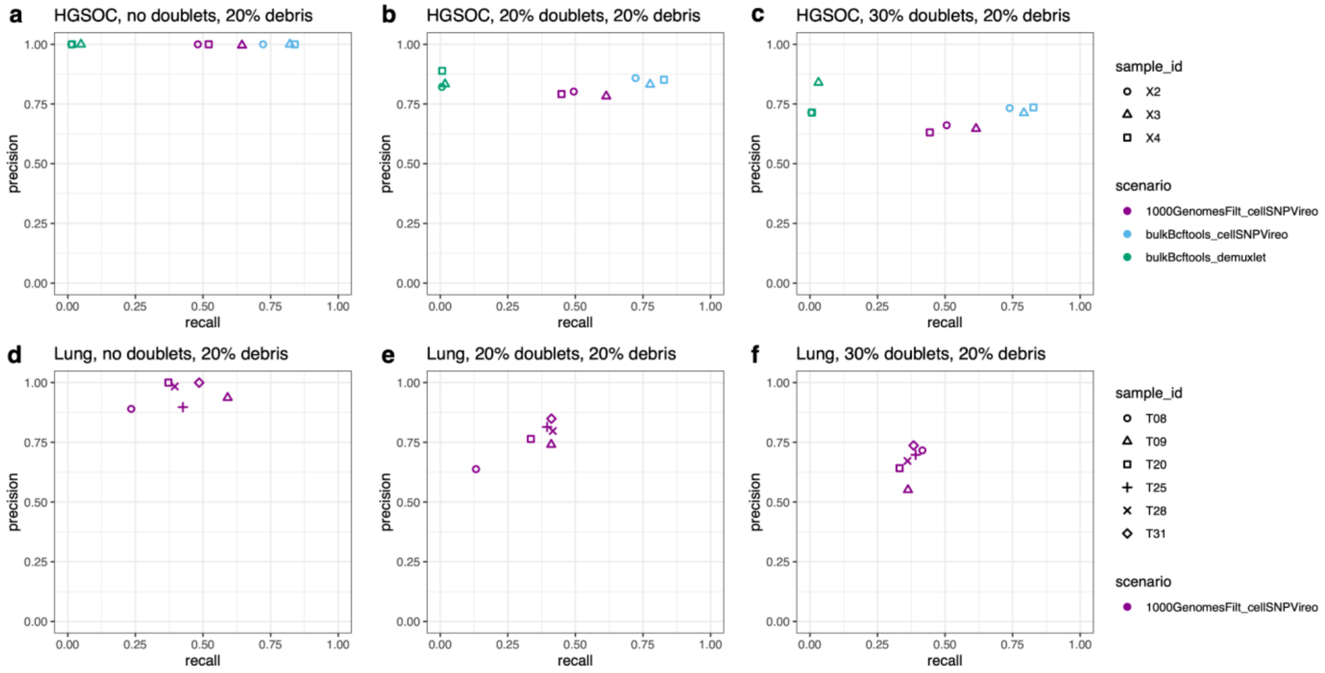

Supplementary Figure 1. Performance evaluations for benchmark scenarios including ambient RNA from simulated cell debris. Top-performing and computationally efficient scenarios for HGSOC dataset **(a-c)** and lung adenocarcinoma dataset **(d-f)**, across three proportions of simulated doublets (no doublets, 20% doublets, 30% doublets), after introducing ambient RNA from simulated cell debris by assigning all reads from 20% of final cell barcodes to other randomly selected cell barcodes. Performance is evaluated in terms of precision (y-axis) and recall (x-axis) for recovering the sample identities of true singlet cells from each scRNA-seq sample. Benchmark scenarios are labeled by color and with the naming scheme “genotypeMethod_demultiplexingMethod”. Samples within each dataset are identified with shapes. Axis limits range from 0 to 1 for all panels.

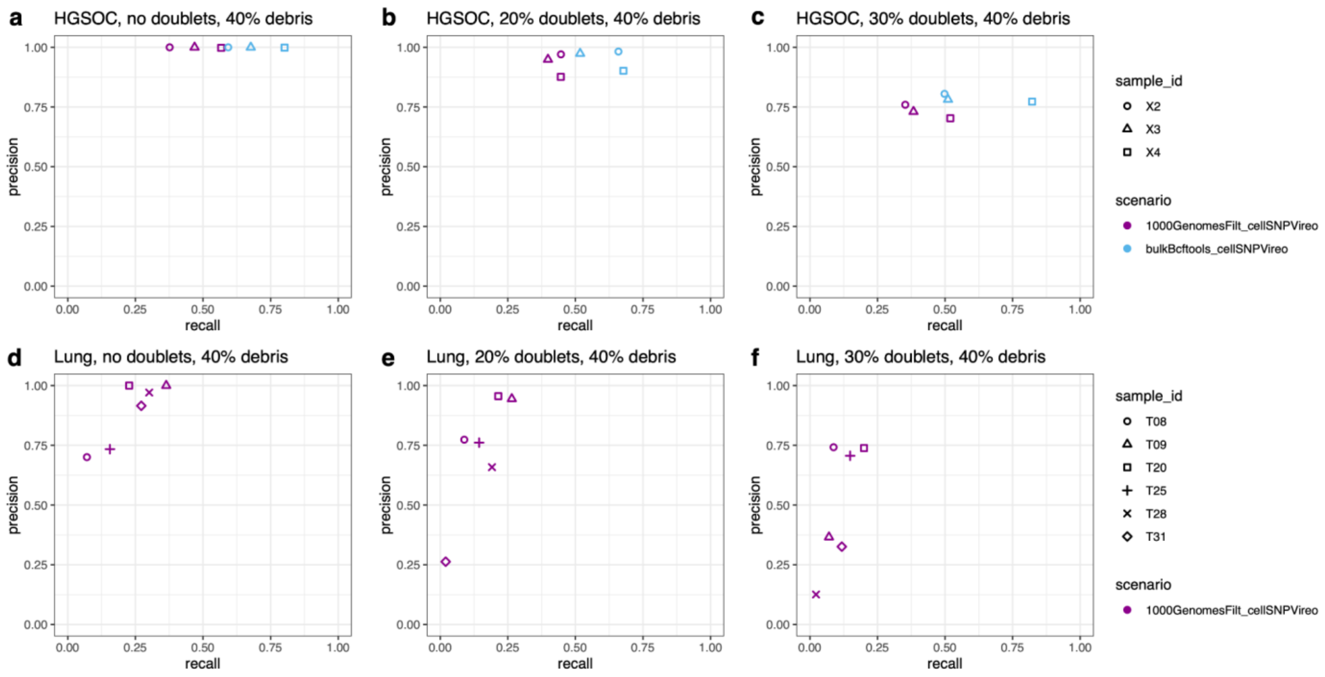

Supplementary Figure 2. Performance evaluations for benchmark scenarios including ambient RNA from simulated cell debris. Top-performing and computationally efficient scenarios for HGSOC dataset **(a-c)** and lung adenocarcinoma dataset **(d-f)**, across three proportions of simulated doublets (no doublets, 20% doublets, 30% doublets), after introducing ambient RNA from simulated cell debris by assigning all reads from 40% of final cell barcodes to other randomly selected cell barcodes. Note that unlike Figure 3 and Supplementary Figure 1, demuxlet is not included for the HGSOC dataset, since this tool did not successfully run with this higher proportion of ambient RNA. Performance is evaluated in terms of precision (y-axis) and recall (x-axis) for recovering the sample identities of true singlet cells from each scRNA-seq sample. Benchmark scenarios are labeled by color and with the naming scheme “genotypeMethod_demultiplexingMethod”. Samples within each dataset are identified with shapes. Axis limits range from 0 to 1 for all panels.

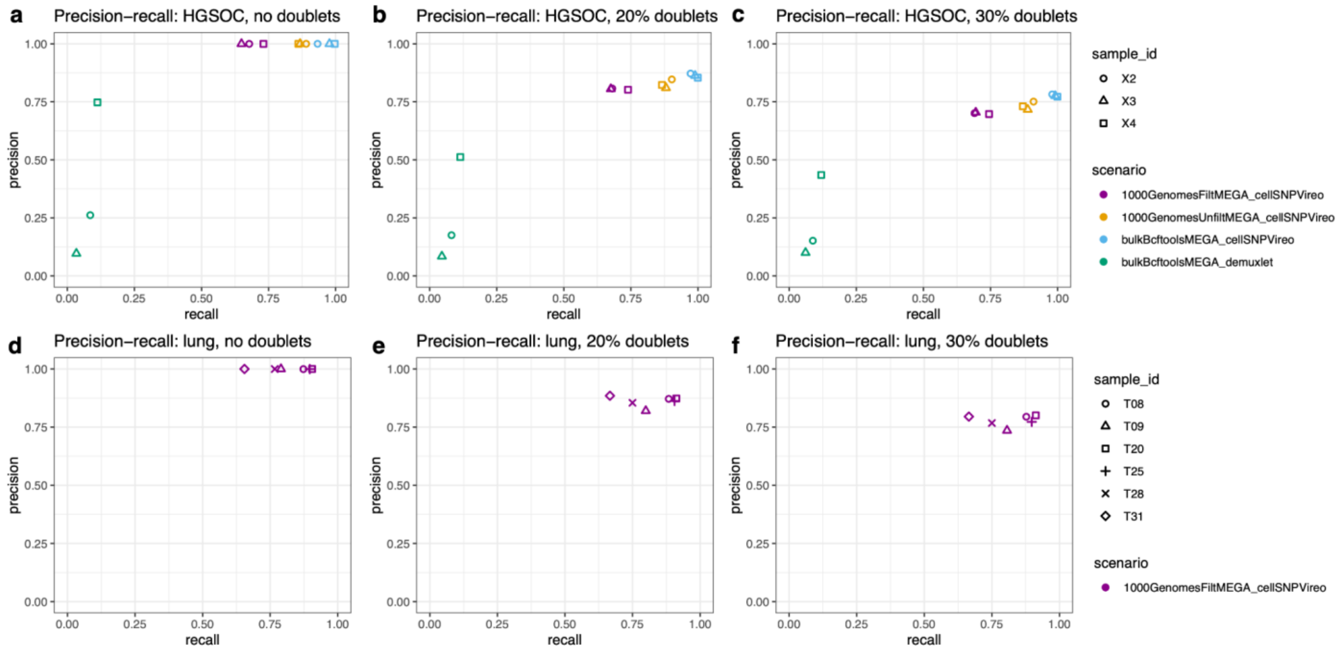

Supplementary Figure 3. Performance evaluations for benchmark scenarios using subset of SNPs from SNP array as genotype reference. Top-performing and computationally efficient scenarios for HGSOC dataset **(a-c)** and lung adenocarcinoma dataset **(d-f)**, across three proportions of simulated doublets (no doublets, 20% doublets, 30% doublets), when using a subset of SNPs from a SNP array (Infinium Multi-Ethnic Global-8 v1.0 array from the Multi-Ethnic Genotyping Array Consortium (MEGA) Consortium) overlapping with either the 1000 Genomes filtered ("1000GenomesFiltMEGA"), 1000 Genomes unfiltered ("1000GenomesUnfiltMEGA"), or bulk RNA-seq ("bulkBcftoolsMEGA") reference as the genotype reference for demultiplexing. Performance is evaluated in terms of precision (y-axis) and recall (x-axis) for recovering the sample identities of true singlet cells from each scRNA-seq sample. Benchmark scenarios are labeled by color and with the naming scheme "genotypeMethod_demultiplexingMethod". Samples within each dataset are identified with shapes. Axis limits range from 0 to 1 for all panels.

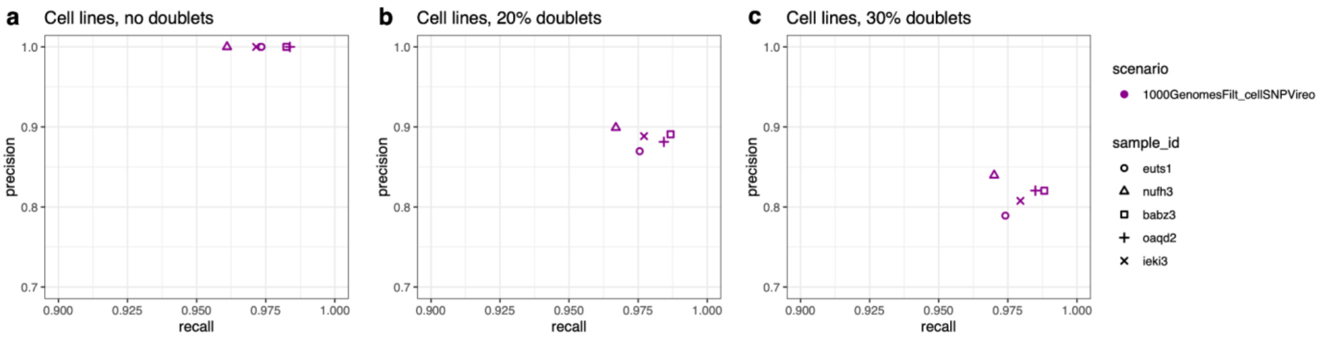

Supplementary Figure 4. Performance evaluations for healthy (non-cancer) cell line dataset. Top-performing and computationally efficient scenario from main results ("1000GenomesFilt_cellSNPVireo") for healthy (non-cancer) induced pluripotent stem cell (iPSC) cell line dataset sourced from [19], consisting of 5 samples, across three proportions of simulated doublets **(a-c)** (no doublets, 20% doublets, and 30% doublets). Performance is evaluated in terms of precision (y-axis) and recall (x-axis) for recovering the sample identities of true singlet cells from each scRNA-seq sample. Benchmark scenarios are labeled by color and with the naming scheme "genotypeMethod_demultiplexingMethod". Samples within each dataset are identified with shapes. Axis limits differ between y-axis and x-axis for improved visibility, and are the same in all panels.

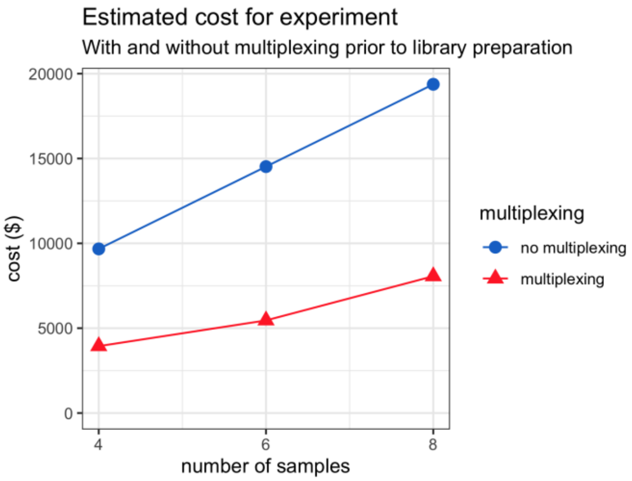

Supplementary Figure 5. Illustration of expected cost savings from multiplexed experimental design prior to library preparation. The figure shows the total of estimated library preparation and sequencing costs, with either no multiplexing or full multiplexing (all samples prepared as a single library and sequenced together), for experiments with 4, 6, or 8 samples. The calculations assume 4,000 desired cells per sample after demultiplexing, after discarding identifiable doublets consisting of cells from multiple samples; library preparation costs of $2,000 per sample or multiplexed set of samples; sequencing costs of $1,500 per 400 million reads with an additional 30% cost due to unaligned reads and adapters; and approximately 20,000 reads per cell. Calculations were performed using the "Cost Per Cell" online calculator provided by the Satija Lab [29].

Supplementary Tables

	bulk RNA-seq (3 HGSOC samples)	1000 Genomes (filtered 3' UTRs)	1000 Genomes (unfiltered)	SNP array (MEGA)
bulk RNA-seq (3 HGSOC samples)	605,367	28,501	356,812	45,843<br>(7.6% of bulk RNA-seq)<br>(2.6% of SNP array)
1000 Genomes (filtered 3' UTRs)		84,853	84,853	14,021<br>(16.5% of 1000 Genomes filtered)<br>(0.8% of SNP array)
1000 Genomes (unfiltered)			7,414,539	638,657<br>(8.6% of 1000 Genomes unfiltered)<br>(36.8% of SNP array)
SNP array (MEGA)				1,733,345

Supplementary Table 1. Number and percentage of SNPs in sets overlapping between the available genotype references (bulk RNA-seq from 3 HGSOC samples, 1000 Genomes filtered to 3' UTRs, 1000 Genomes unfiltered, and MEGA SNP array). The bulk RNA-seq and 1000 Genomes filtered references are used for the main results.

	Number of cells per sample	Median genes detected per cell	Median UMI counts per cell
HGSOC dataset			
X2	7,123	1,194	3,404
X3	1,533	2,045	6,616
X4	6,546	1,498	4,304
Lung dataset			
T08	4,093	1,078	3,427
T09	4,267	1,172	3,618
T20	4,521	1,047	2,924
T25	4,428	934	3,038
T28	5,789	979	2,720
T31	7,069	814	2,008
Cell line dataset			
euts1	5,535	4,755	24,496
nufh3	9,820	3,340	14,331
babz3	14,373	2,923	10,850
oaqd2	7,870	3,946	17,140
ieki3	8,109	4,337	20,139

Supplementary Table 2. Summary of HGSOC, lung adenocarcinoma [38], and healthy induced pluripotent stem cells (iPSC) cell line [19] datasets. Number of cells per sample, median genes detected per cell, and median unique molecular identifier (UMI) counts per cell are shown for each dataset. Median genes detected and median UMI counts per cell were higher for the cell line dataset than for the HGSOC and lung adenocarcinoma datasets.

HGSOC, 30% doublets, bulkBcftools_cellSNPVireo					
	donor1	donor0	donor2	doublet	unassigned
X2	3839	0	8	7	1
X2-X2	757	0	1	2	0
X3	0	826	0	0	0
X3-X3	0	38	0	0	0
X4	0	0	3505	0	0
X4-X4	0	0	647	0	0
dbl-X2-X3	46	107	0	162	1
dbl-X2-X4	223	0	343	859	7
dbl-X3-X4	0	114	41	160	0
% true identifiable doublets	5.5%	20.4%	8.4%	99.2%	88.9%

Supplementary Table 3. Confusion matrix for singlet, doublet, and unassigned calls for the top-performing scenario (cellSNP/Vireo with bulk RNA-seq genotype reference) for 30% doublets scenario for HGSOC dataset (matching the precision-recall values in the main results shown in **Figure 2 c**). Calls by Vireo are shown in columns (singlets: donor0, donor1, donor2 in arbitrary sample order; doublets; unassigned), and true labels from the simulation are shown in rows (singlets: X2, X3, X4; non-identifiable doublets from the same sample: X2-X2, X3-X3, X4-X4; identifiable doublets: dbl-X2-X3, dbl-X2-X4, dbl-X3-X4). Doublets consisting of two cells from the same sample are non-identifiable since these cells contain the same germline SNPs.

HGSOC, 30% doublets, bulkBcftools_demuxlet					
	X2	X3	X4	doublet	ambiguous
X2	2560	0	0	993	0
X2-X2	510	0	0	221	0
X3	0	340	0	486	0
X3-X3	0	13	0	25	0
X4	0	1	1712	1776	3
X4-X4	0	0	302	345	0
dbl-X2-X3	15	14	0	287	0
dbl-X2-X4	38	0	40	1225	0
dbl-X3-X4	0	19	7	289	0
% true identifiable doublets	1.7%	8.5%	2.3%	31.9%	0.0%

Supplementary Table 4. Confusion matrix for singlet, doublet, and ambiguous calls for demuxlet (with bulk RNA-seq genotype reference) for 30% doublets scenario for HGSOC dataset (matching the precision-recall values in the main results shown in **Figure 2 c**). Calls by demuxlet are shown in columns (singlets: X2, X3, X4; doublets; ambiguous), and true labels from the simulation are shown in rows (singlets: X2, X3, X4; non-identifiable doublets from the same sample: X2-X2, X3-X3, X4-X4; identifiable doublets: dbl-X2-X3, dbl-X2-X4, dbl-X3-X4). Doublets consisting of two cells from the same sample are non-identifiable since these cells contain the same germline SNPs.

	HGSOC, 20% doublets			HGSOC, 30% doublets	
	identified as doublet			identified as doublet	
true doublet	TRUE	FALSE	true doublet	TRUE	FALSE
TRUE	600	**1,003**	TRUE	917	**1,408**
FALSE	**3,720**	6,405	FALSE	**3,214**	4,965

	lung, 20% doublets			lung, 30% doublets	
	identified as doublet			identified as doublet	
true doublet	TRUE	FALSE	true doublet	TRUE	FALSE
TRUE	346	**2,221**	TRUE	326	**3,328**
FALSE	**2,088**	17,678	FALSE	**1,129**	14,798

Supplementary Table 5. Summary of doublets identified by applying a downstream doublet detection tool (scDblFinder) to demultiplexed cells after applying top-performing and computationally efficient demultiplexing tools (cellSNP/Vireo with bulk RNA-seq reference for HGSOC dataset; cellSNP/Vireo with 1000 Genomes 3' UTRs filtered reference for lung adenocarcinoma dataset), for 20% and 30% doublets scenarios. scDblFinder was run using default settings, and clusters representing doublets identified using thresholds of 70 (HGSOC) and 10 (lung adenocarcinoma) differentially expressed genes per cluster based on inspection of elbow plots. False positive and false negative doublet calls are shown in bold font.

Figure 1 - schematic

Click here to access/download;Figure;Figure_1_s

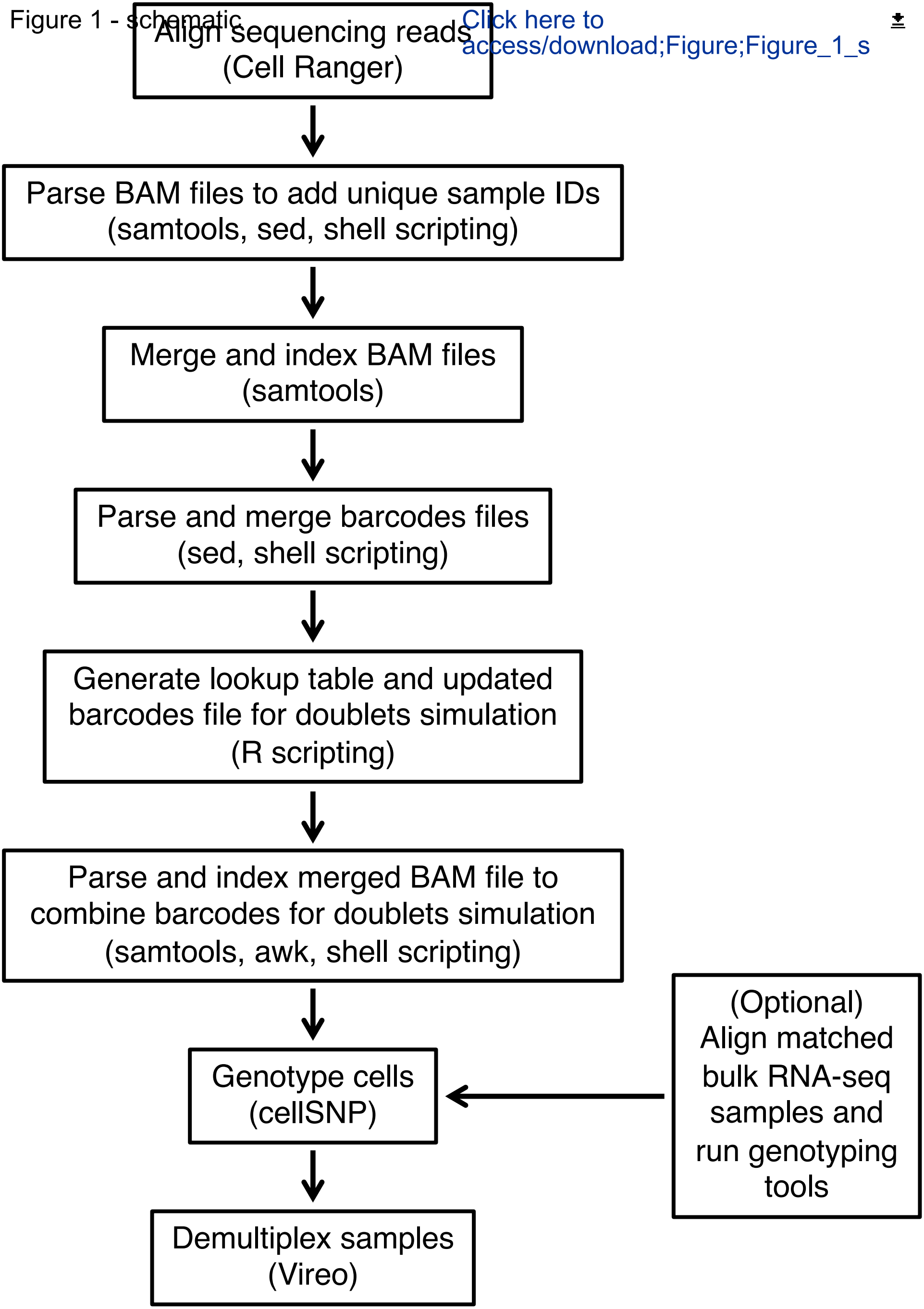

Figure 2 results

Click here to access/download;Figure;Figure_2_results_main.pdf

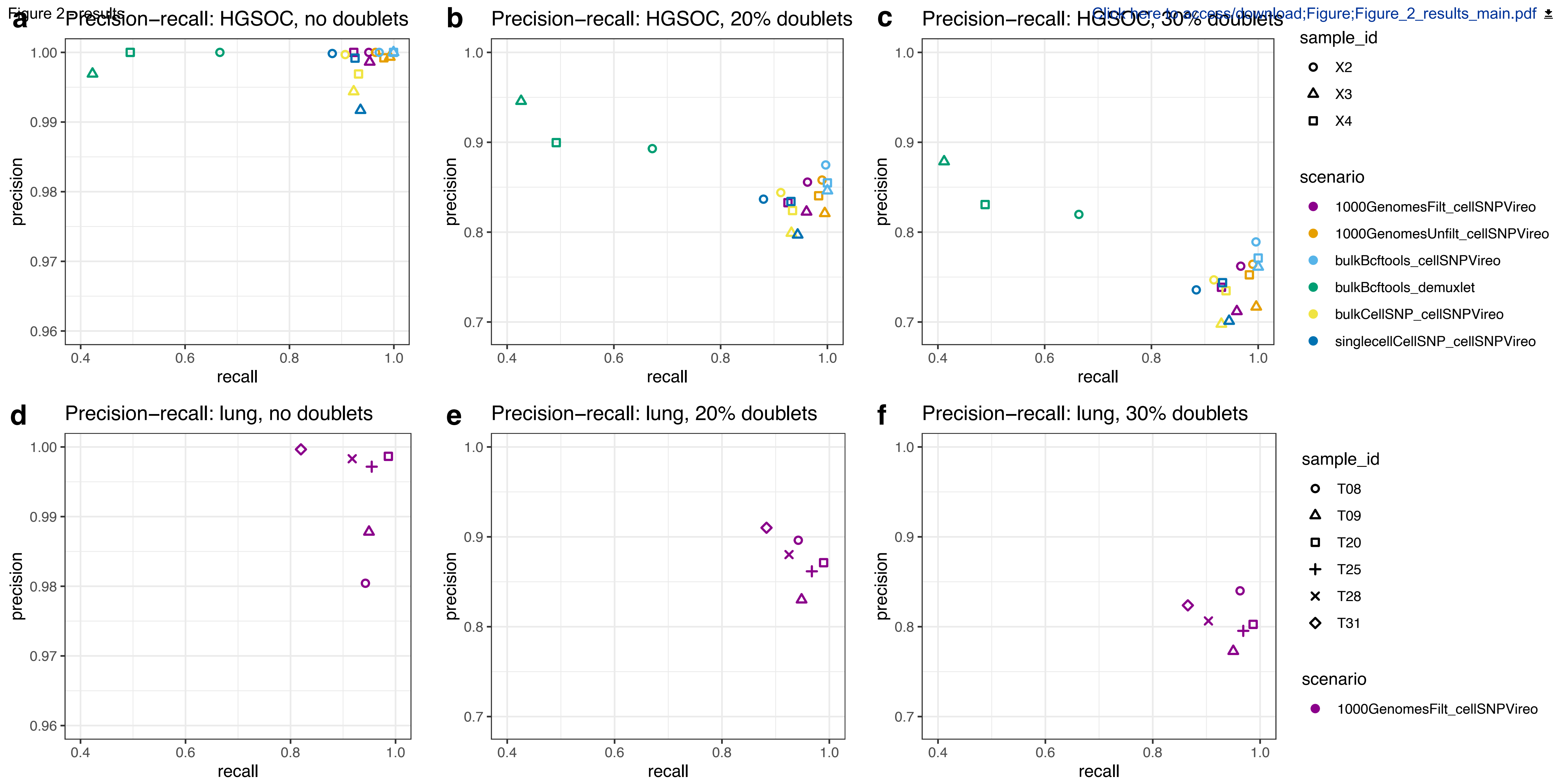

Figure 3 - results

Click here to access/download;Figure;Figure_3_results_debris.pdf

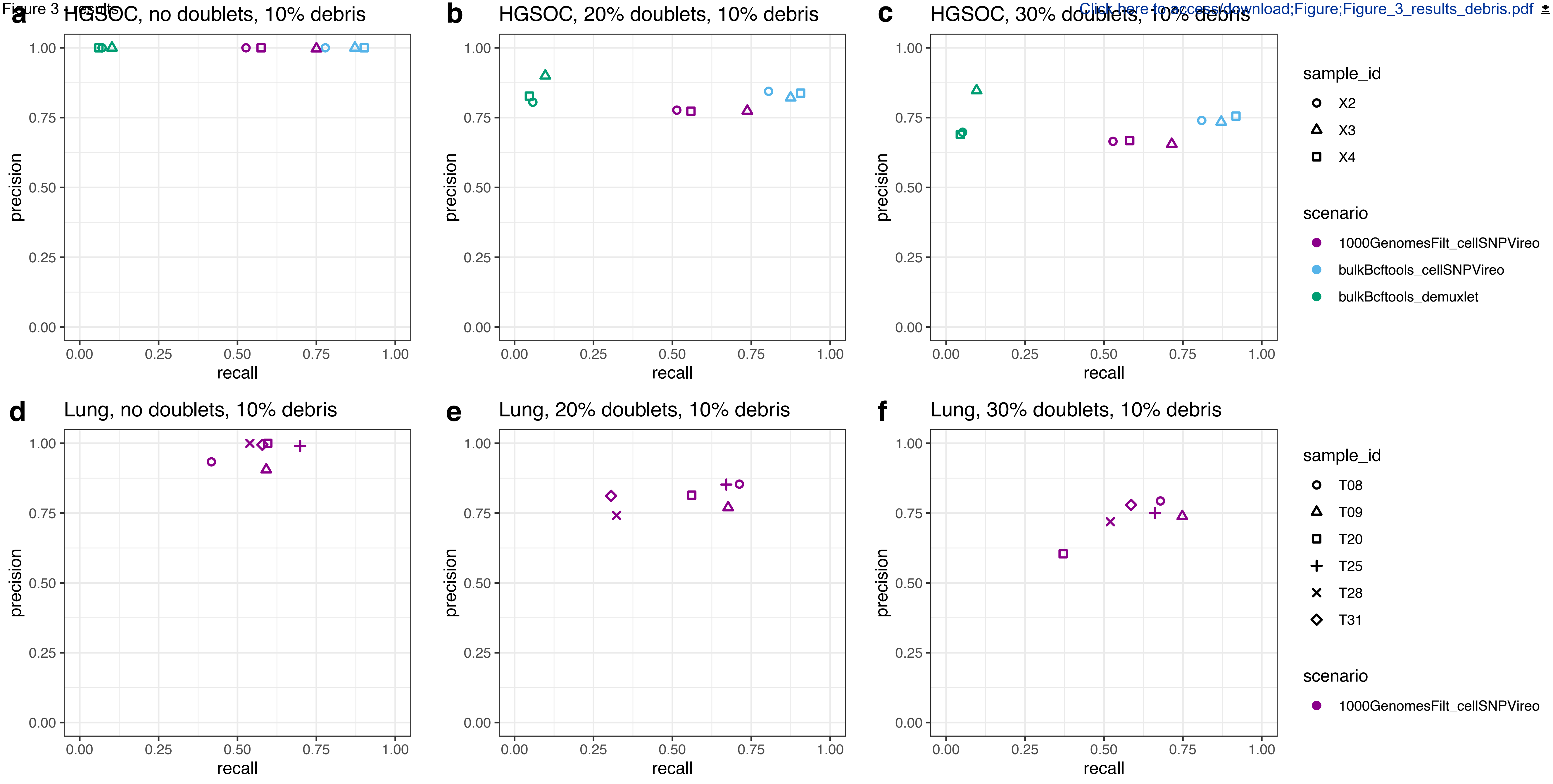

Figure 4 - runtimes

Click here to access/download;Figure;Figure_4_runtimes.pdf

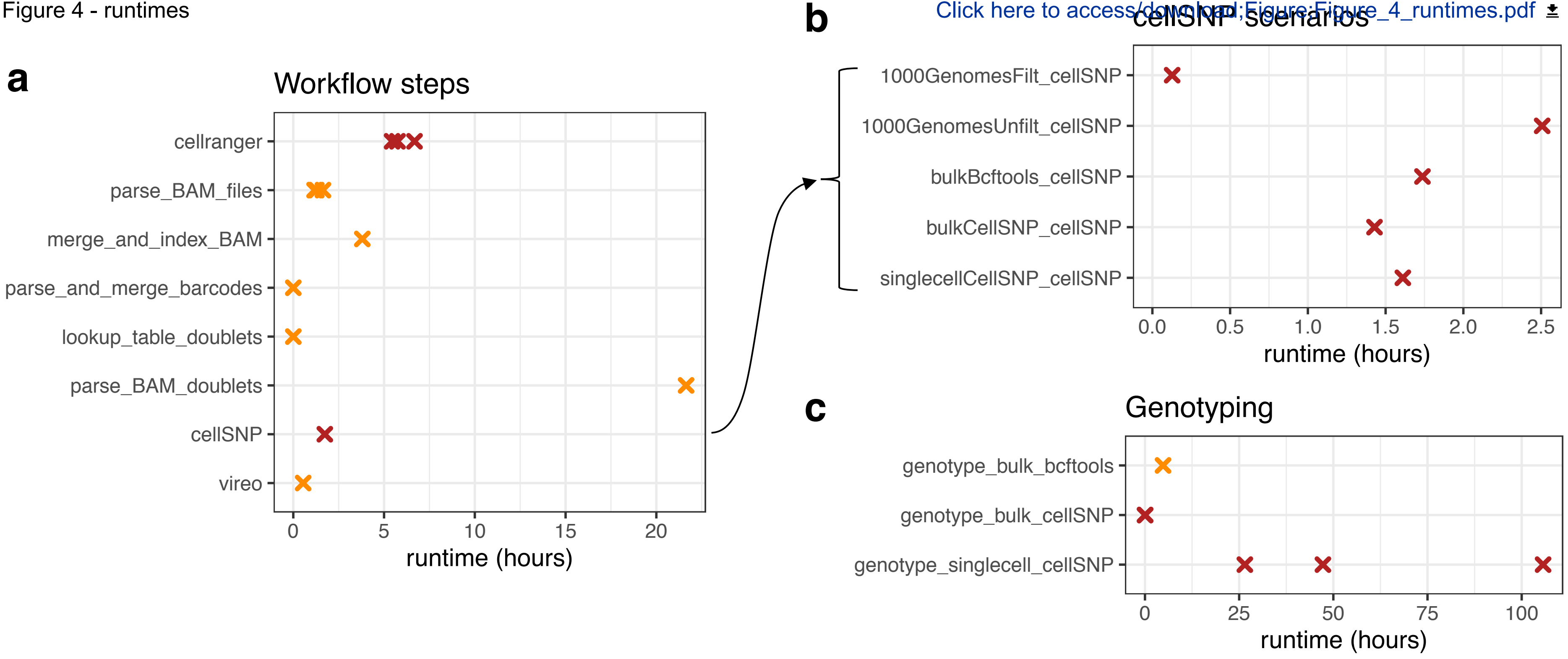

Supplementary Figure 1

Click here to access/download;Figure;Supplementary_Figure_1_debris_20pc.pdf

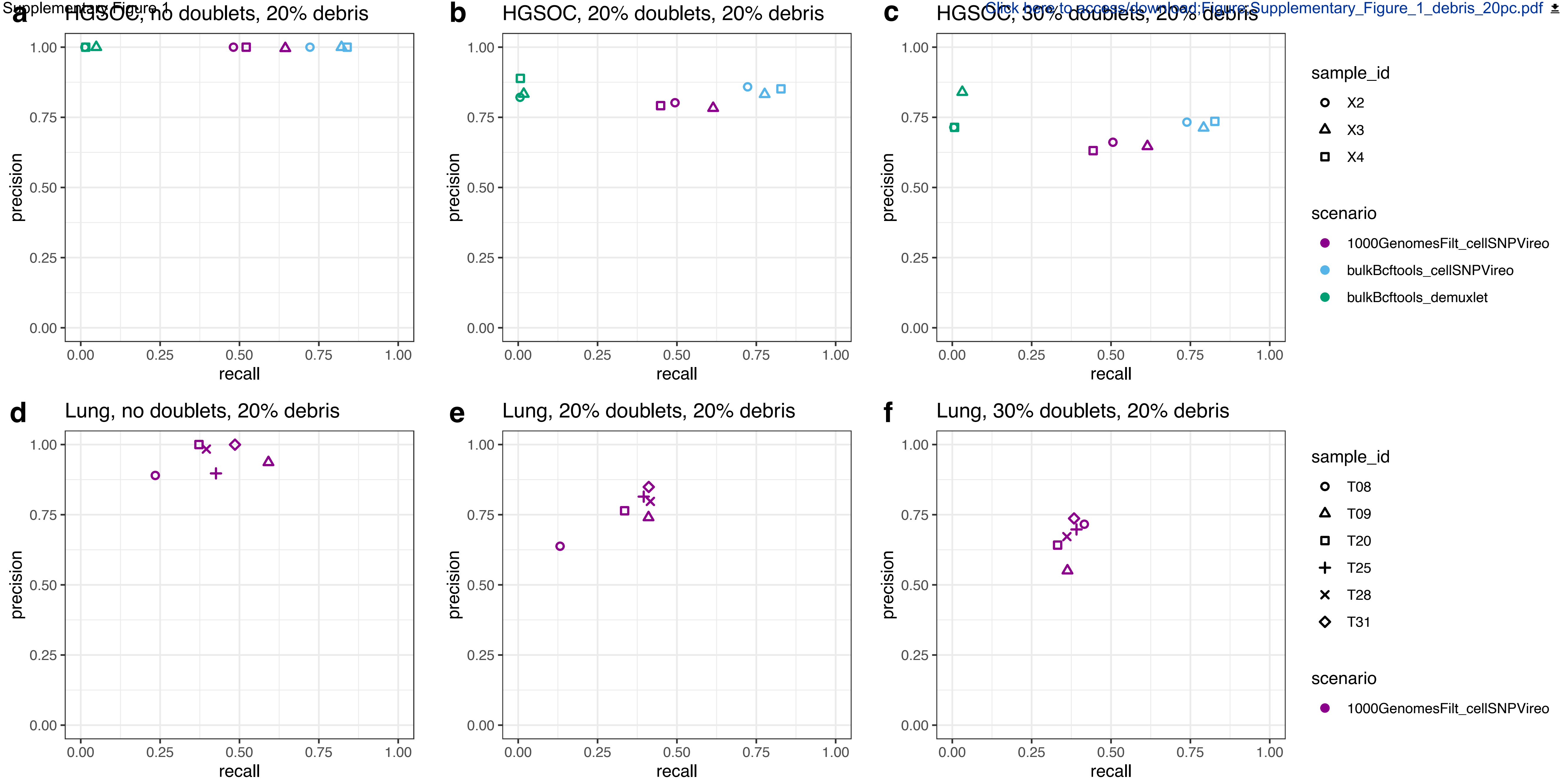

Click here to access/download;Figure;Supplementary_Figure_2_debris_40pc.pdf

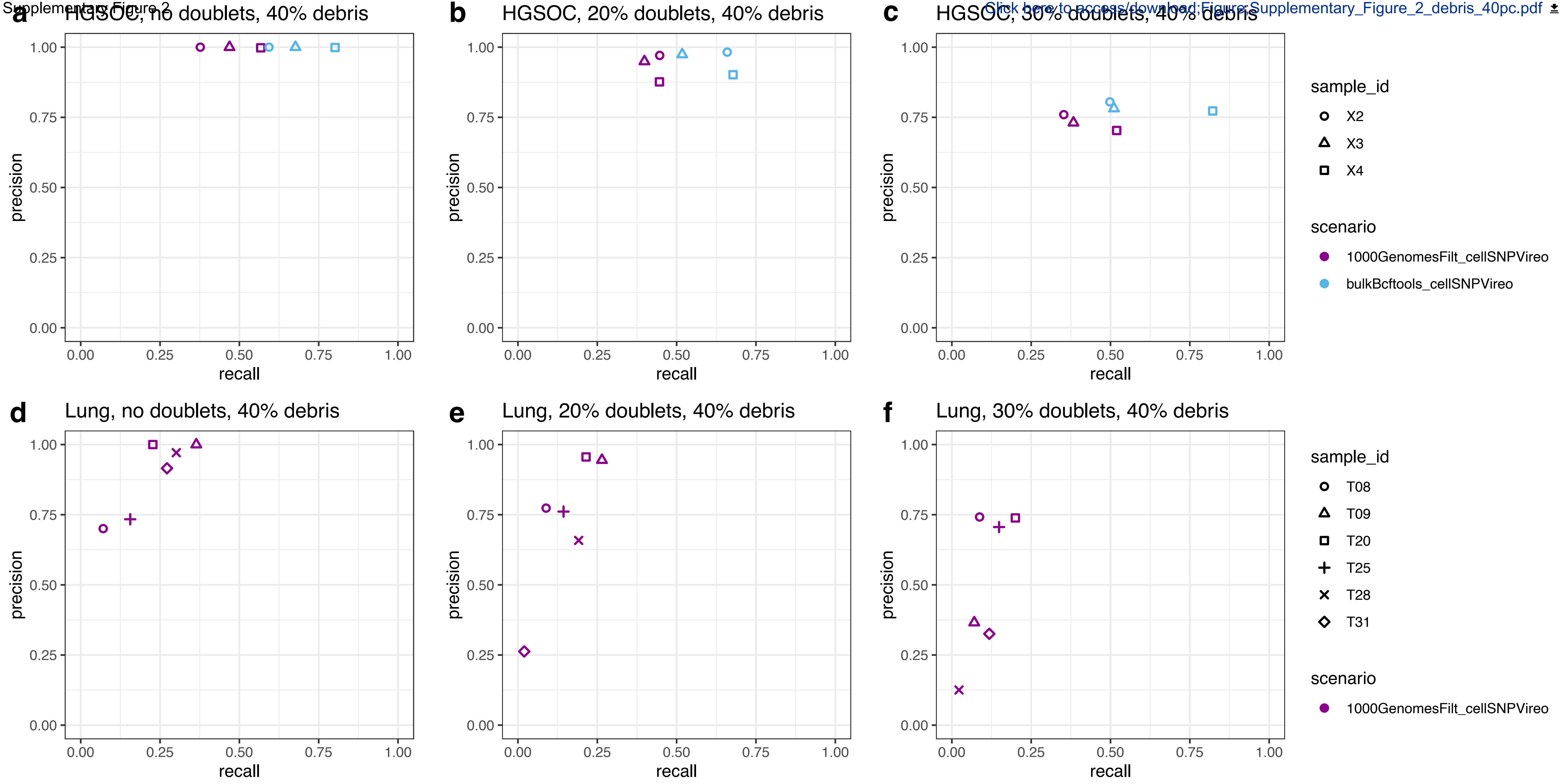

Supplementary Figure 3

Click here to access/download;Figure;Supplementary_Figure_3_SNP_array.pdf

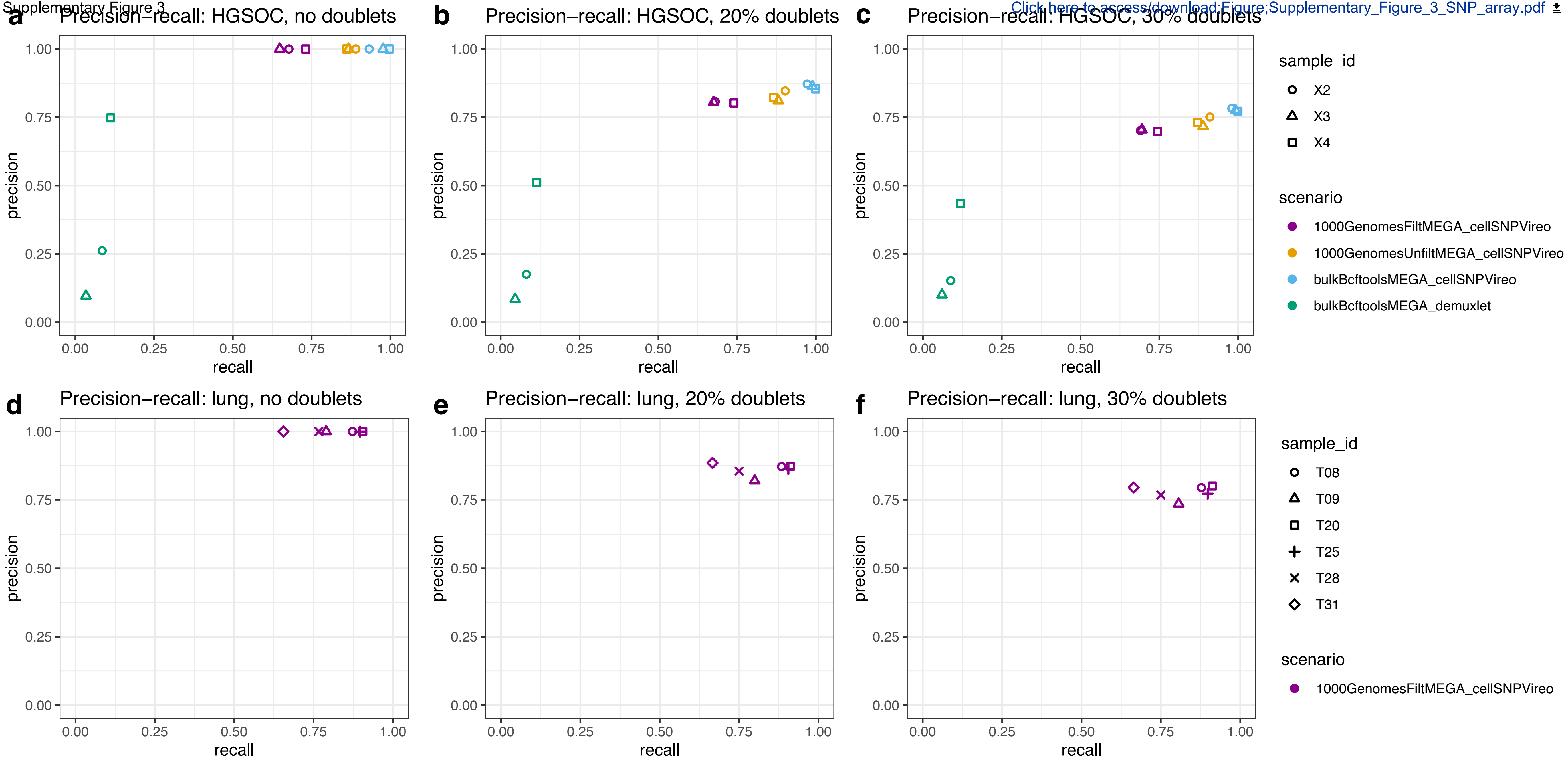

Click here to access/download;Figure;Supplementary_Figure_4_healthy.pdf

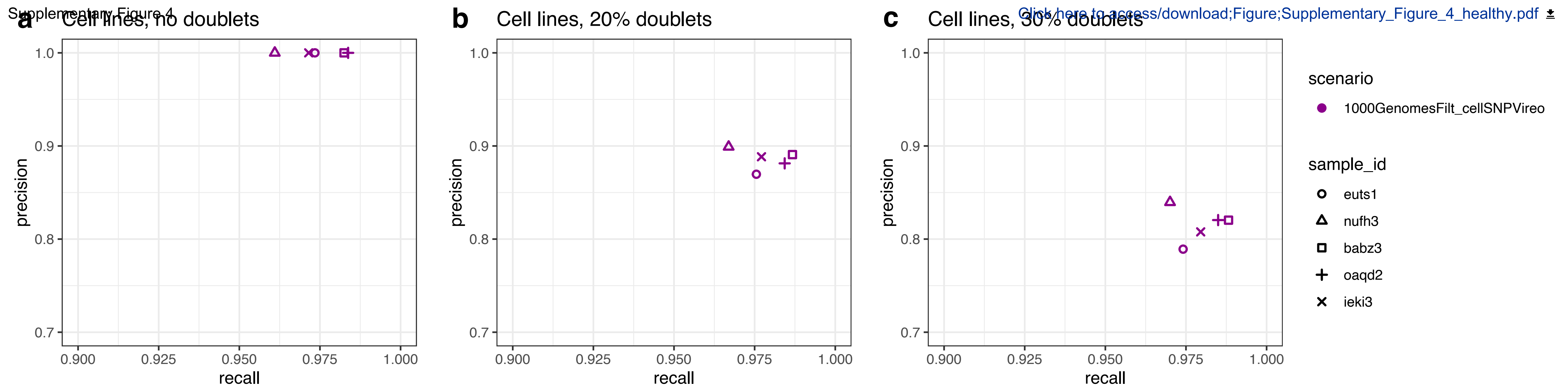

Supplementary Figure 5

Click here to access/download;Figure;Suppleme

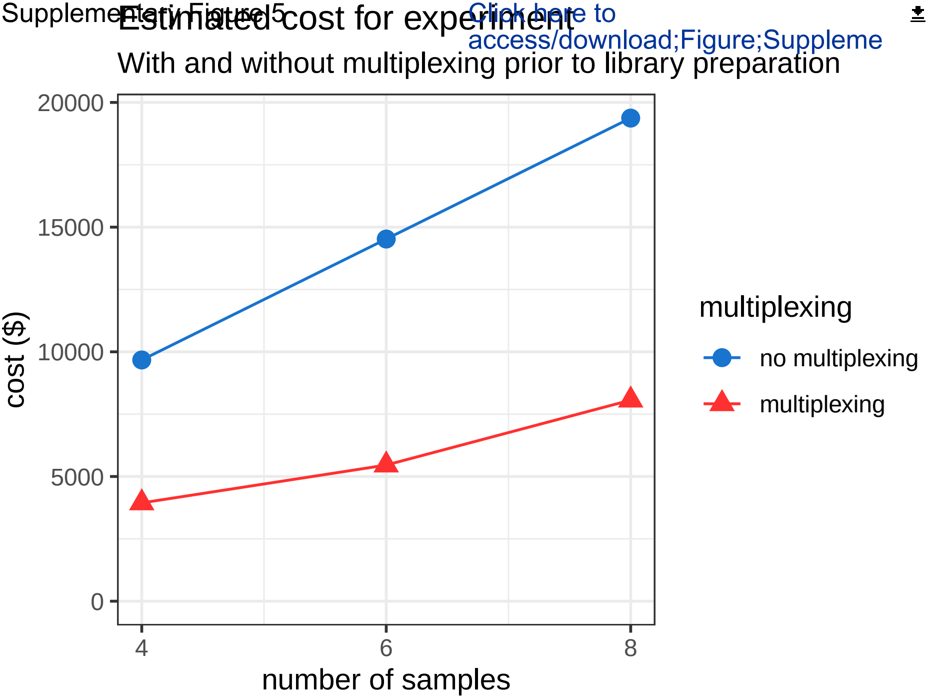

Author response and cover letter
Click here to access/download;Personal Cover;Demultiplexing_author_response_2021-07-19.pdf

July 19, 2021

Dear Dr. Zauner,

Revised submission GIGA-D-21-00074

Thank you for your consideration of our revised manuscript entitled "Genetic demultiplexing of pooled single-cell RNA-sequencing samples in cancer facilitates effective experimental design" to *GigaScience* (**GIGA-D-21-00074**). We appreciate the reviewers' constructive comments, and in our enclosed revision have addressed the comments raised from our original submission.

Briefly, we have included several additional sets of analyses, which strengthen our results and conclusions, and provide useful additional information for readers. In particular, in response to an excellent suggestion by Reviewer 2, we have included evaluation scenarios containing proportions of ambient RNA from simulated cell debris or lysed cells, which may be common in cancer. This reduced overall demultiplexing performance somewhat (although the effect was minimized in our top-performing scenarios), suggesting the importance of considering experimental procedures to remove cell debris from pooled single-cell RNA-seq samples. In addition, we have included scenarios to evaluate demultiplexing performance when using a subset of reference SNPs from a SNP array as the reference; evaluated performance on a healthy cell-line dataset as a baseline comparison; provided additional details on the types of errors in the doublet calls used for the precision and recall calculations in the evaluations; and investigated the use of downstream doublet detection tools. Additional details on these analyses are provided in the response to reviewers below and in the revised manuscript.

Overall, our updated analyses confirm our central conclusion -- genetic variation-based demultiplexing of single-cell RNA-seq samples pooled prior to library preparation works well in the cancer context, especially when reference SNP profiles from matched bulk RNA-seq samples are available -- while providing important additional information about performance in specific settings, such as high proportions of ambient RNA or when using a SNP array reference.

We believe that the modifications in our revised submission have led to a stronger and more coherent manuscript, with strengthened and more balanced evidence for our claims, and we look forward to your reply. All revised text in the manuscript is marked in blue font. Below you will find our point-by-point responses to the reviewers.

Best wishes,

Stephanie Hicks

Stephanie Hicks
Department of Biostatistics
Johns Hopkins Bloomberg School of Public Health
615 N Wolfe St.
Baltimore, MD 21205

<u>Response to Reviewers</u>

<u>Reviewer 1</u>:

Reviewer #1: The authors have investigated methods to demultiplex samples from single-cell RNA sequencing data based on SNPs. Pooling of cancer samples is certainly an important tool for cost reduction and minimizing batch effects as the authors have pointed out. It nice to see the well documented, freely available github repository.

Major Points:
The authors have tested demultiplexing based on matched reference data, publicly available SNPs and without any SNP reference. It has to be noted however, that without a matching reference the samples can be demultiplexed but not assigned to a specific donor. This has to be mentioned in the text as this is a crucial feature.

This point has now been mentioned more clearly in the Introduction (paragraph 2) and Discussion (final paragraph).

In the cancer context it would be important to test also reference data from a SNP array in addition to bulk RNA-seq data. It would be interesting to see which matched reference would perform better. Could the authors provide such an analysis for the HGSOC dataset?

This is an important point, which we had not previously considered. Since we did not have access to SNP array data for the HGSOC samples, we designed an additional simulation strategy based on the existing samples, to test the performance when using a SNP array. In brief, we found that using reference data from a SNP array performs remarkably well and we describe the additional analyses and figures that have now been added to the manuscript in response to this excellent suggestion.

We downloaded the set of SNPs from a widely used SNP array (the Infinium Multi-Ethnic Global-8 v1.0 array from the Multi-Ethnic Global Array or MEGA Consortium, which includes 1.7 million SNPs), and then calculated the sets of overlapping SNPs between the SNP array and (i) our bulk RNA-seq genotype reference (605,367 total SNPs before calculating overlap) and (ii) the 1000 Genomes reference filtered to 3' UTRs (84,853 total SNPs before calculating overlap). Then, we re-ran the benchmark evaluations using these smaller sets of overlapping SNPs. We note that this strategy effectively simulates a worst-case scenario for the SNP array, since we are using only a small subset of the SNPs from the array (stating this another way, we anticipate the following results would improve if we had access to the full 1.7 million SNP array). Compared to the SNP array, the overlapping sets contain 2.6% of SNPs (bulk RNA-seq) or 0.8% of SNPs (1000 Genomes filtered); while compared to our original references the overlapping sets contain 7.6% (bulk RNA-seq) or 16.5% (1000 Genomes filtered) of the original SNPs (see Supplementary Table 1 for a summary of the set sizes).

Despite the extreme reduction in number of SNPs compared to the full array or the original references, demultiplexing performance for cellSNP/Vireo remains remarkably high, especially when using the bulk RNA-seq reference SNPs. However, the performance of demuxlet drops substantially. (See Supplementary Figure 3; Results and Methods). Since we are using only a small subset of the array

SNPs, these results give us confidence that good demultiplexing performance can be achieved when using SNP arrays in the cancer context.

The increased robustness of cellSNP/Vireo compared to demuxlet is also consistent with our observations for the additional supplementary analyses, e.g. adding ambient RNA from simulated debris or lysed cells, as described below in comments for Reviewer 2.

Could the authors run a real demultiplex experiment in addition to mixing reads in silico (e.g. for the HGSOC samples)?

We thank the reviewer for this comment and agree an experimental evaluation (as opposed to an *in silico* evaluation) would strengthen the results. Unfortunately, it was not possible to generate additional experimental data within the revision timeline for this manuscript. However, we have now included additional analyses in our revised manuscript that significantly strengthen the evidence for our central claim: genetic variation-based demultiplexing works well in the cancer context.

In particular, this claim is supported by the additional results of (i) subset of SNPs from SNP array (see above), (ii) simulated ambient RNA from debris or lysed cells (see comments for Reviewer 2 below), and (iii) confirmation of baseline performance in non-cancer data (comments for Reviewer 2), all of which demonstrate that cellSNP/Vireo maintain performance in settings that we had not previously tested.

Finally, we have now expanded the Discussion to note that additional experimental data could be used to further strengthen these claims, if it becomes available. (We have also mentioned that the main difficulty when working with experimental data will be in determining a reliable ground truth.)

Can the authors comment on the number of doublets identified with their technique and run at least one doublet detection tool in comparison? For HGSOC (n=3) half of all doublet should be mixed doublets and half harbours cells from the same sample.

We have added additional results and discussion to address this point, which was also raised by Reviewer 2. (Previously, this information was only included implicitly within the precision and recall calculations.) In Supplementary Tables 3 and 4, we have included tables comparing the true and false doublet calls by sample for the top-performing set of tools (cellSNP/Vireo with bulk RNA-seq reference) and the comparison with demuxlet (with bulk RNA-seq reference) for the HGSOC dataset with 30% doublets. This shows that the doublet calls from cellSNP/Vireo are relatively pure (average of 99.2% true identifiable doublets among called doublets), while the reduction in overall precision is due to additional true identifiable doublets incorrectly called as singlets. By contrast, demuxlet returns a much lower percentage (31.9%) of true identifiable doublets among the doublet calls.

We also ran a recent doublet detection tool (scDblFinder; https://bioconductor.org/packages/scDblFinder) as an illustration to see if we could use these tools to identify any remaining doublets after demultiplexing. However, this did not perform well, giving large proportions of both false positives and false negatives (Supplementary Table 5). We hypothesize that this may be due to the nature of cancer samples, where clustering on expression profiles returns more varied clusters than in non-cancer data. While there are benchmark evaluations of doublet detection tools in the non-cancer setting (e.g. https://doi.org/10.1016/j.cels.2020.11.008), we feel a

comprehensive evaluation of doublet detection tools applied here (e.g. using interactive plots and investigating additional possible reasons for reduced performance in cancer compared to non-cancer data) is outside the scope of this manuscript. Finally, we have extended the Discussion to mention these limitations more clearly, and to suggest directions for future work in this area.

Minor points:
In the discussion the authors state that they compared the best performing tools against alternative tools (page 15, line 344) - however it is essentially cellSNPVireo vs demuxlet (one vs another). Should be clearer in the text.

We have clarified this sentence, and expanded the Introduction and Discussion to make it more clear that we are comparing these two tools, and not performing a comprehensive benchmark for all available tools.

Can the authors comment on hashing and how hashing would perform in comparison to genetic demultiplexing in the discussion?

In general, we would expect cell hashing to be the gold standard in terms of performance, while the performance of genetic variation-based demultiplexing depends largely on the computational tool used and the quality of the genotype reference. However, genetic variation-based demultiplexing also provides significant advantages in terms of simpler sample preparation and cost savings during library preparation. We have added additional text to expand on this in the Discussion, and have also mentioned cell hashing approaches in the Introduction.

Reviewer 2:

Reviewer #2: This paper addresses an important and untested question of whether single cell genotype demultiplexing works on cancer samples.

1. The authors use in-silico mixtures of individual single cell cancer experiments with varying percentages of doublet cell barcodes. While it is understandable due to the "finite and irreplaceable nature of tumor cells", it potentially reduces the value, accuracy, and applicability to real data especially due to my next issue. To me this is not a deal breaker, but a distinct limitation.

We agree that demonstrating demultiplexing performance on real experimental data would further strengthen our conclusions, compared to relying on simulations. However, while acknowledging this limitation, our expanded set of simulation scenarios provides strong evidence for our central claim that genetic variation-based demultiplexing tools perform well for cancer data.

In particular, as mentioned above for Reviewer 1, our additional supplementary analyses on (i) using a subset of overlapping SNPs from the MEGA SNP array (see comments for Reviewer 1), (ii) ambient RNA from debris or lysed cells, and (iii) confirmation of performance in healthy samples, significantly strengthen our evidence for our conclusions compared to our original manuscript. We thank the reviewers for these suggestions.

We have also ensured that our wording in the Introduction and Discussion makes it clear that our results are based on simulations generated by modifying real data.

2. Ambient RNA from lysed cells prior to partitioning is not addressed in either simulation or discussion. I think that it should be at least discussed as necrosis is not uncommon in cancer samples. It is important to note that as ambient RNA increases, demuxlet quickly begins classifying almost everything as a doublet while other methods (vireo, souporcell) are more robust to this type of noise.

This was an important omission in our previous *in silico* benchmark evaluation, and we thank the reviewer for this suggestion. We have now added additional scenarios that include ambient RNA from simulated cell debris or lysed cells by computationally assigning all sequencing reads from a certain percentage of cell barcodes (10%, 20%, or 40%) to other randomly selected cell barcodes, and re-running the demultiplexing tools (results included as new Figure 2 and Supplementary Figures 1-2). These results demonstrate that overall demultiplexing performance decreases in terms of recall, although the effect is minimized when using the top-performing set of tools (cellSNP/Vireo with bulk RNA-seq reference). As suggested above, the performance of demuxlet drops much more substantially, suggesting that cellSNP/Vireo is more robust to this type of noise. We have included additional discussion of this issue in Results and Discussion. In particular, this suggests the importance of considering experimental techniques such as straining to reduce cell debris in cancer samples, when possible.

3. A full normal sample is not shown for comparison to cancer samples. This could be taken from free available datasets. For instance the Hipsci datasets from my souporcell paper could be used. You would want to try to normalize for UMI/cell with subsampling and match roughly the number of cells per individual and then of course match the doublet rate. The number and type of cancer samples tested are fairly limited but adequate for an initial evaluation.

We thank the reviewer for this comment. We have now incorporated additional simulations based on the five individual iPSC cell lines in the souporcell paper (https://doi.org/10.1038/s41592-020-0820-1), as a healthy (non-cancer) baseline comparison, including both 20% and 30% doublet scenarios. Normalizing the UMI counts per sample became too computationally intensive, so we have instead reported additional details (number of cells per sample, detected genes per cell, UMI counts per cell) for all three datasets (HGSOC, lung, cell line) (Supplementary Table 2) to ensure that comprehensive information is provided for readers to compare the datasets. Performance in the cell line data (Supplementary Figure 4) was comparable to the main benchmark scenarios, giving us confidence that our simulation framework can be reliably applied in both cancer and non-cancer settings, and providing a useful additional baseline comparison that is consistent with previous published results in non-cancer data.

3. I think the author's results actually warrant a stronger conclusion in the question of fully supervised and genotype model based methods (demuxlet, which assumes allele fractions must match the stated genotype) vs cluster center based and thus free allele fraction methods (vireo, souporcell etc). This also has a theoretical advantage in cancer samples which will have non-standard allele fractions around copy number alterations and somatic mutations. Vireo clearly outperforms demuxlet in accurate doublet detection (demuxlet's rigid model system results in over-calling doublets which is presumably what is crushing demuxlet's recall in some samples).

We have now provided additional details on performance in terms of types of doublet calls for both cellSNP/Vireo and demuxlet (Supplementary Tables 3 and 4), as well as the additional results for ambient RNA and subsets of SNPs from an array (Figure 2 and Supplementary Figures 1-3). As suggested by the reviewer, cellSNP/Vireo consistently outperforms demuxlet by a wide margin, especially in these more difficult simulation scenarios. We have included additional wording in Results and Discussion to make these comparisons between the two tools more clear for readers.

4. The paper mentions that the authors prefer high recall over precision but does not discuss the potential downsides of low precision.

We have included additional text to clarify this. Specifically, we prefer to retain all true singlet cells (high recall) if possible, since there is the possibility of applying additional downstream analyses (visualizations, doublet detection tools) to identify any remaining doublets (which is one of the possible sources of reduced precision). In addition, we have included illustrative tables of results comparing the types of doublet calls for both cellSNP/Vireo (top-performing scenario with bulk RNA-seq reference, HGSOC, 30% doublets; Supplementary Table 3) and demuxlet (with bulk RNA-seq reference; Supplementary Table 4), as suggested below, which provides additional information on the types of errors. As suggested by Reviewer 1, we also ran a recent doublet detection tool (scDblFinder; https://bioconductor.org/packages/scDblFinder), but this did not perform well, giving large proportions of both false positives and false negatives (Supplementary Table 5), possibly due to the more varied expression in cancer data compared to non-cancer affecting clustering performance. While benchmarks of doublet detection tools exist for non-cancer data (e.g. https://doi.org/10.1016/j.cels.2020.11.008), in our view a comprehensive evaluation of doublet detection tools applied here is outside the scope of this manuscript.

5. Most importantly I think it would also be useful to discuss how low recall and precision could be attained and in outlier samples discuss which error modes were observed. You could attain low recall due to 1. high unassigned cells. 2. overcalling doublets. 3. misassigned cells. And you could attain low precision through 1. misassigned cells 2. undercalling doublets. These different error modes are very different and could affect downstream processing and inference in different ways. I think that it is important to tease out which of these phenomenon is going on with each sample. I grant that it does not lend itself to standard statistical metrics such as precision/recall as there are more than 2 states of truth (single cell correct sample, single cell incorrect sample, doublet cell). A confusion matrix heatmap would be one option but showing this for many samples would be information overload. At the very least a discussion of the outlier samples should have a breakdown of these numbers with a discussion of why this happens (as explained previously). This would be useful for the demuxlet runs on HGSDC explaining low recall and for the relatively low recall on all runs for the HGSDC 30% doublet run.

We thank the reviewer for this suggestion. This information was previously only implicitly contained within the precision and recall calculations, and we agree it is informative to provide an illustration of these error modes for readers. We have included tables of results comparing the types of true and false singlet and doublet calls for two illustrative scenarios: the top-performing set of tools (cellSNP/Vireo with bulk RNA-seq reference) in HGSOC with 30% doublets (Supplementary Table 3), and the comparison for demuxlet (with bulk RNA-seq reference, HGSOC, 30% doublets) (Supplementary Table 4). This illustrates the differences in the types of doublet calls between the two tools. For cellSNP/Vireo, the doublet calls are relatively pure, with 99.2% of called doublets being true identifiable doublets. By

contrast, for demuxlet, this percentage is much lower at 31.9%. These tables also illustrate the distinction between identifiable doublets (two cells from different donors, with different SNP profiles) and non-identifiable doublets (two cells from the same donor, which cannot be distinguished by their SNP profiles). For cellSNP/Vireo, almost all the *non*-identifiable doublets are assigned to the correct individual sample, while for demuxlet these contain additional false positive doublet calls.

6. One specific comment I have is with regards to the following sentence. "More fundamentally, due to the reliance on genetically distinct SNP profiles, genetic demultiplexing tools are expected to work well for human samples from unrelated individuals, but are not applicable to biological samples from inbred mice or hereditary related human populations, or samples from the same individual [3]" Almost all of this is true, but in our souporcell paper we demultiplexed a maternal/fetal sample so these tools are applicable to related humans. Of course power decreases the more related the individuals are (in the case of inbreeding). Perhaps the wording could be changed.

We have re-worded this sentence to clarify this issue, as suggested.

Cover letter (original submission) Click here to access/download;Personal Cover;In_silico_demultiplexing_cover_letter.pdf

Simon Edmunds, PhD
Editor-in-Chief, *GigaScience*

Dear Dr. Edmunds,

Please find enclosed our manuscript submission titled "Genetic demultiplexing of pooled single-cell RNA-sequencing samples in cancer facilitates effective experimental design", for consideration by *GigaScience* as a Research article.

Recent experimental designs have been proposed for single-cell RNA-sequencing (scRNA-seq) where tissue samples are pooled prior to library preparation (instead of after library preparation and prior to sequencing, which is the standard approach in scRNA-seq). Previous work has shown computational demultiplexing algorithms (e.g. Vireo[1] and demuxlet[2]) for this experimental design work well in healthy tissues as well as cancer cell lines from distinct cancer types. Advantages of these demultiplexing algorithms include significant cost savings during library preparation, as well as the ability to reduce batch effects as samples are not processed in separate libraries. However, to the best of our knowledge, this has not yet been shown for the most difficult setting of pooled cancer samples from multiple individuals within the same cancer type, where additional somatic variants might adversely affect the demultiplexing performance.

To address this, here we investigate whether these recently developed computational demultiplexing methods that rely on genetic variation between biological samples from different individuals can be used with these new experimental designs for scRNA-seq in the cancer setting.
Using the raw sequencing scRNA-seq reads from two cancer types with high copy number variation (CNV) and high single nucleotide variation (SNV) burden (HGSOC and lung adenocarcinoma, respectively), we performed a series of simulations with doublets (two cells per droplet) that are likely to occur when overloading the sequencing machines with combined samples. Our evaluations showed excellent demultiplexing performance (recall: 99.9% and precision: 85.9%, averaged across 3 samples and assuming 20% doublets), especially when matched bulk RNA-seq samples are available for genotyping. We believe this is a useful result for readers of GigaScience and the larger single-cell community as it will give researchers the justification to proceed with genetic variation-based demultiplexing designs for scRNA-seq experiments in cancer.

In addition, we have taken great care to develop a reproducible simulation framework and analysis workflow for our benchmark evaluations. This includes a Snakemake workflow consisting of the best-performing set of tools identified in our benchmark evaluation (which can be run with a single command on a Linux cluster), as well as reproducible scripts for all other benchmark scenarios and evaluations. We provide these resources to allow other researchers to adapt our framework to perform similar pilot evaluations for experimental design planning purposes in their own experiments. Our workflow is modular, allowing alternative tools to be substituted. All code is freely available from our code repository on GitHub (https://github.com/lmweber/snp-dmx-cancer).

[1] Huang et al. (2019), Genome Biology: https://pubmed.ncbi.nlm.nih.gov/31836005/
[2] Kang et al. (2018), Nature Biotechnology: https://pubmed.ncbi.nlm.nih.gov/29227470/

The raw data files (FASTQ files) used as input for our simulations contain patient-identifiable information (genetic variants), and are available by controlled access from the Database of Genotypes and Phenotypes (dbGaP) (accession phs002262.v1.p1) and the European Genome-phenome Archive (EGA) (EGAD00001005054). In addition, we have made non-identifiable gene count tables publicly available from the Gene Expression Omnibus (GEO) (GSE158937).

All authors have approved the manuscript for publication, and declare that they do not have any competing interests. The manuscript is not currently under consideration for publication with any other journal.

We are confident that your readers will find this study to be a compelling and scientifically useful result for the single-cell and cancer research communities, as well as a useful and adaptable simulation framework and code and data resource, and will therefore find the submitted manuscript highly relevant.

Thank you for your consideration of the manuscript.

Best wishes,

Stephanie Hicks

Stephanie Hicks
Department of Biostatistics
Johns Hopkins Bloomberg School of Public Health
Johns Hopkins Data Science Lab
