# Supplementary material for: Genetic demultiplexing of pooled single-cell RNA-sequencing samples in cancer facilitates effective experimental design
Source: Gigascience. 2021 Sep 22;10(9):giab062. doi: 10.1093/gigascience/giab062 (PMC8458035; doi:10.1093/gigascience/giab062)
Supplement: giab062_Supplemental_File [file giab062_supplemental_file.pdf]

Supplementary Figures

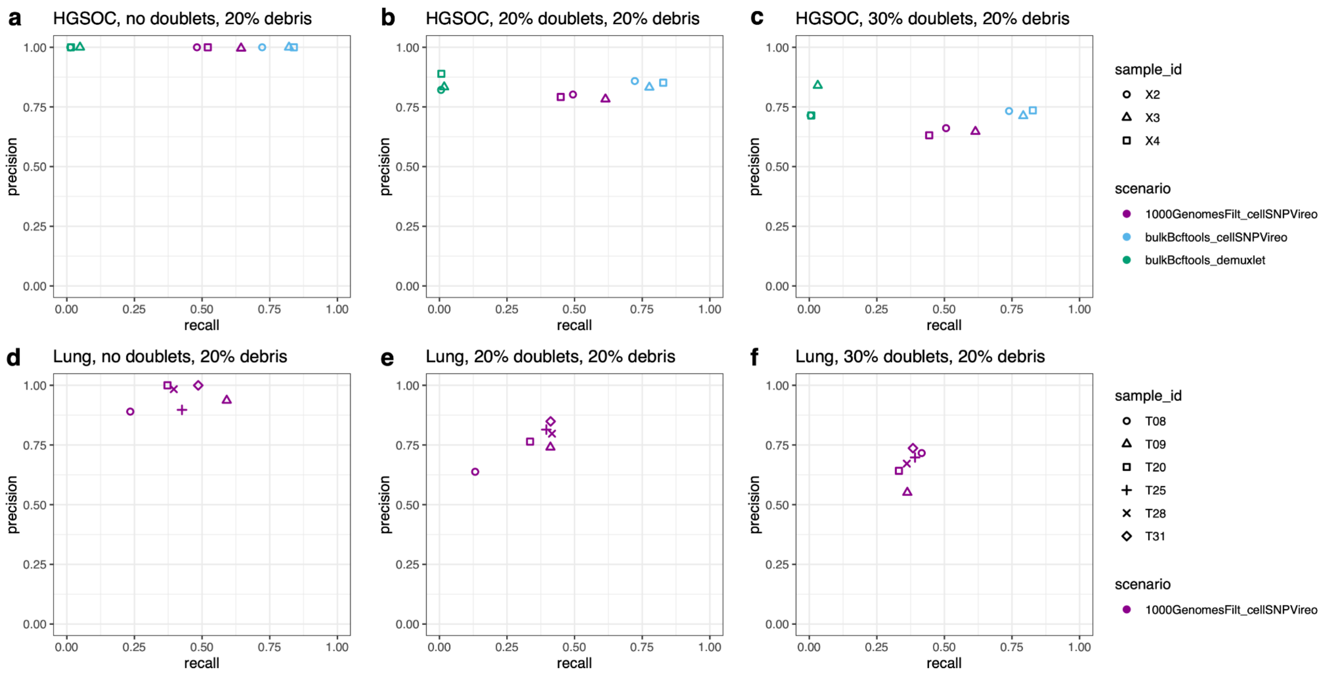

Supplementary Figure S1. Performance evaluations for benchmark scenarios including ambient RNA from simulated cell debris. Top-performing and computationally efficient scenarios for HGSOC dataset **(a-c)** and lung adenocarcinoma dataset **(d-f)**, across three proportions of simulated doublets (no doublets, 20% doublets, 30% doublets), after introducing ambient RNA from simulated cell debris by assigning all reads from 20% of final cell barcodes to other randomly selected cell barcodes. Performance is evaluated in terms of precision (y-axis) and recall (x-axis) for recovering the sample identities of true singlet cells from each scRNA-seq sample. Benchmark scenarios are labeled by color and with the naming scheme “genotypeMethod_demultiplexingMethod”. Samples within each dataset are identified with shapes. Axis limits range from 0 to 1 for all panels.

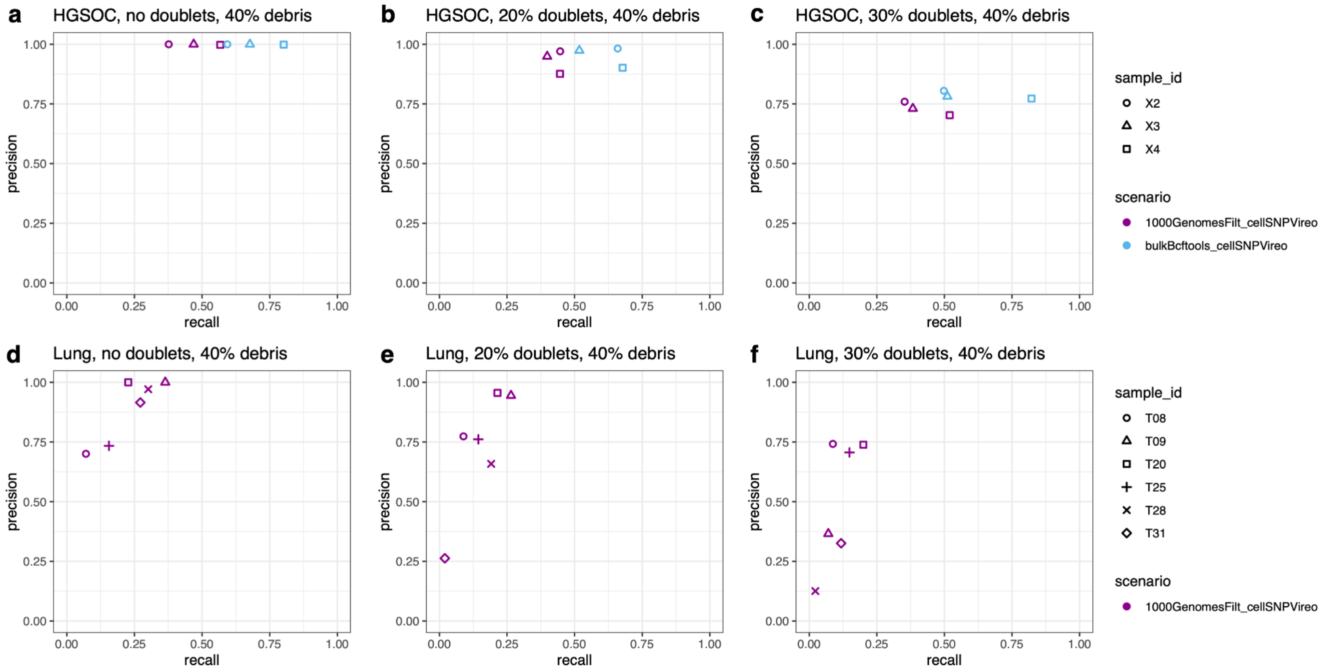

Supplementary Figure S2. Performance evaluations for benchmark scenarios including ambient RNA from simulated cell debris. Top-performing and computationally efficient scenarios for HGSOC dataset **(a-c)** and lung adenocarcinoma dataset **(d-f)**, across three proportions of simulated doublets (no doublets, 20% doublets, 30% doublets), after introducing ambient RNA from simulated cell debris by assigning all reads from 40% of final cell barcodes to other randomly selected cell barcodes. Note that unlike Figure 3 and Supplementary Figure 1, demuxlet is not included for the HGSOC dataset, since this tool did not successfully run with this higher proportion of ambient RNA. Performance is evaluated in terms of precision (y-axis) and recall (x-axis) for recovering the sample identities of true singlet cells from each scRNA-seq sample. Benchmark scenarios are labeled by color and with the naming scheme “genotypeMethod_demultiplexingMethod”. Samples within each dataset are identified with shapes. Axis limits range from 0 to 1 for all panels.

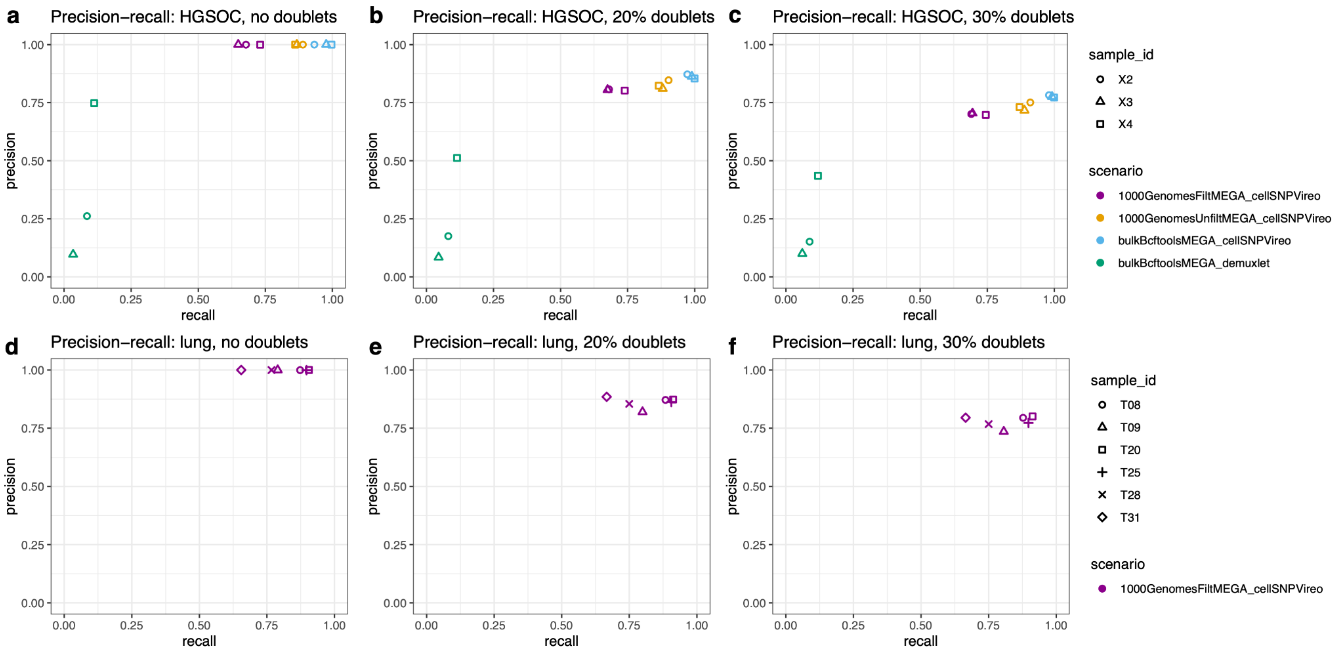

Supplementary Figure S3. Performance evaluations for benchmark scenarios using subset of SNPs from SNP array as genotype reference. Top-performing and computationally efficient scenarios for HGSOC dataset **(a-c)** and lung adenocarcinoma dataset **(d-f)**, across three proportions of simulated doublets (no doublets, 20% doublets, 30% doublets), when using a subset of SNPs from a SNP array (Infinium Multi-Ethnic Global-8 v1.0 array from the Multi-Ethnic Genotyping Array Consortium (MEGA) Consortium) overlapping with either the 1000 Genomes filtered ("1000GenomesFiltMEGA"), 1000 Genomes unfiltered ("1000GenomesUnfiltMEGA"), or bulk RNA-seq ("bulkBcftoolsMEGA") reference as the genotype reference for demultiplexing. Performance is evaluated in terms of precision (y-axis) and recall (x-axis) for recovering the sample identities of true singlet cells from each scRNA-seq sample. Benchmark scenarios are labeled by color and with the naming scheme "genotypeMethod_demultiplexingMethod". Samples within each dataset are identified with shapes. Axis limits range from 0 to 1 for all panels.

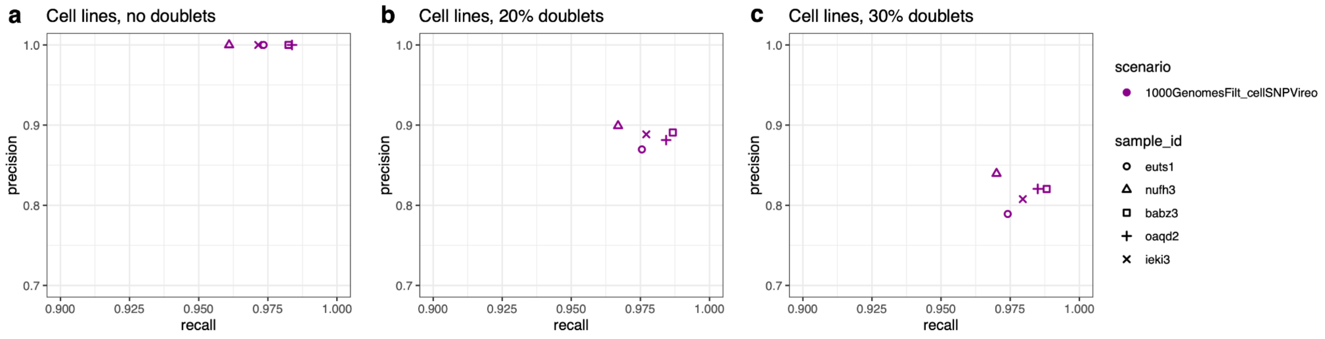

Supplementary Figure S4. Performance evaluations for healthy (non-cancer) cell line dataset. Top-performing and computationally efficient scenario from main results ("1000GenomesFilt_cellSNPVireo") for healthy (non-cancer) induced pluripotent stem cell (iPSC) cell line dataset sourced from [19], consisting of 5 samples, across three proportions of simulated doublets **(a-c)** (no doublets, 20% doublets, and 30% doublets). Performance is evaluated in terms of precision (y-axis) and recall (x-axis) for recovering the sample identities of true singlet cells from each scRNA-seq sample. Benchmark scenarios are labeled by color and with the naming scheme "genotypeMethod_demultiplexingMethod". Samples within each dataset are identified with shapes. Axis limits differ between y-axis and x-axis for improved visibility, and are the same in all panels.

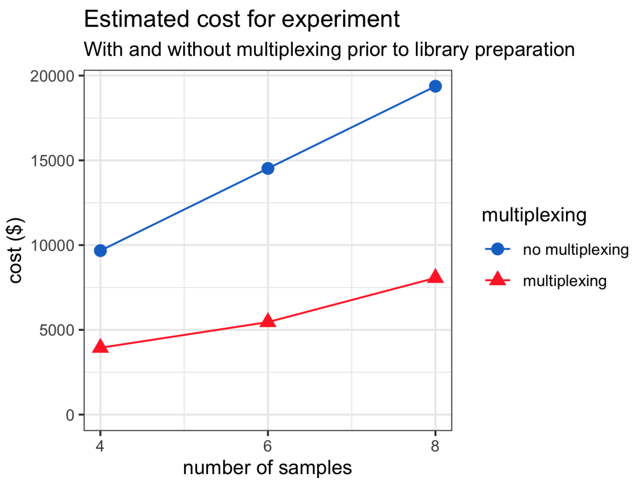

Supplementary Figure S5. Illustration of expected cost savings from multiplexed experimental design prior to library preparation. The figure shows the total of estimated library preparation and sequencing costs, with either no multiplexing or full multiplexing (all samples prepared as a single library and sequenced together), for experiments with 4, 6, or 8 samples. The calculations assume 4,000 desired cells per sample after demultiplexing, after discarding identifiable doublets consisting of cells from multiple samples; library preparation costs of $2,000 per sample or multiplexed set of samples; sequencing costs of $1,500 per 400 million reads with an additional 30% cost due to unaligned reads and adapters; and approximately 20,000 reads per cell. Calculations were performed using the “Cost Per Cell” online calculator provided by the Satija Lab [29].

Supplementary Tables

	bulk RNA-seq (3 HGSOC samples)	1000 Genomes (filtered 3' UTRs)	1000 Genomes (unfiltered)	SNP array (MEGA)
bulk RNA-seq (3 HGSOC samples)	605,367	28,501	356,812	45,843<br>(7.6% of bulk RNA-seq)<br>(2.6% of SNP array)
1000 Genomes (filtered 3' UTRs)		84,853	84,853	14,021<br>(16.5% of 1000 Genomes filtered)<br>(0.8% of SNP array)
1000 Genomes (unfiltered)			7,414,539	638,657<br>(8.6% of 1000 Genomes unfiltered)<br>(36.8% of SNP array)
SNP array (MEGA)				1,733,345

Supplementary Table S1. Number and percentage of SNPs in sets overlapping between the available genotype references (bulk RNA-seq from 3 HGSOC samples, 1000 Genomes filtered to 3' UTRs, 1000 Genomes unfiltered, and MEGA SNP array). The bulk RNA-seq and 1000 Genomes filtered references are used for the main results.

	Number of cells per sample	Median genes detected per cell	Median UMI counts per cell
HGSOC dataset			
X2	7,123	1,194	3,404
X3	1,533	2,045	6,616
X4	6,546	1,498	4,304
Lung dataset			
T08	4,093	1,078	3,427
T09	4,267	1,172	3,618
T20	4,521	1,047	2,924
T25	4,428	934	3,038
T28	5,789	979	2,720
T31	7,069	814	2,008
Cell line dataset			
euts1	5,535	4,755	24,496
nufh3	9,820	3,340	14,331
babz3	14,373	2,923	10,850
oaqd2	7,870	3,946	17,140
ieki3	8,109	4,337	20,139

Supplementary Table S2. Summary of HGSOC, lung adenocarcinoma [39], and healthy induced pluripotent stem cells (iPSC) cell line [19] datasets. Number of cells per sample, median genes detected per cell, and median unique molecular identifier (UMI) counts per cell are shown for each dataset. Median genes detected and median UMI counts per cell were higher for the cell line dataset than for the HGSOC and lung adenocarcinoma datasets.

HGSOC, 30% doublets, bulkBcftools_cellSNPVireo					
	donor1	donor0	donor2	doublet	unassigned
X2	3839	0	8	7	1
X2-X2	757	0	1	2	0
X3	0	826	0	0	0
X3-X3	0	38	0	0	0
X4	0	0	3505	0	0
X4-X4	0	0	647	0	0
dbl-X2-X3	46	107	0	162	1
dbl-X2-X4	223	0	343	859	7
dbl-X3-X4	0	114	41	160	0
% true identifiable doublets	5.5%	20.4%	8.4%	99.2%	88.9%

Supplementary Table S3. Confusion matrix for singlet, doublet, and unassigned calls for the top-performing scenario (cellSNP/Vireo with bulk RNA-seq genotype reference) for 30% doublets scenario for HGSOC dataset (matching the precision-recall values in the main results shown in **Figure 2 c**). Calls by Vireo are shown in columns (singlets: donor0, donor1, donor2 in arbitrary sample order; doublets; unassigned), and true labels from the simulation are shown in rows (singlets: X2, X3, X4; non-identifiable doublets from the same sample: X2-X2, X3-X3, X4-X4; identifiable doublets: dbl-X2-X3, dbl-X2-X4, dbl-X3-X4). Doublets consisting of two cells from the same sample are non-identifiable since these cells contain the same germline SNPs.

HGSOC, 30% doublets, bulkBcftools_demuxlet					
	X2	X3	X4	doublet	ambiguous
X2	2560	0	0	993	0
X2-X2	510	0	0	221	0
X3	0	340	0	486	0
X3-X3	0	13	0	25	0
X4	0	1	1712	1776	3
X4-X4	0	0	302	345	0
dbl-X2-X3	15	14	0	287	0
dbl-X2-X4	38	0	40	1225	0
dbl-X3-X4	0	19	7	289	0
% true identifiable doublets	1.7%	8.5%	2.3%	31.9%	0.0%

Supplementary Table S4. Confusion matrix for singlet, doublet, and ambiguous calls for demuxlet (with bulk RNA-seq genotype reference) for 30% doublets scenario for HGSOC dataset (matching the precision-recall values in the main results shown in **Figure 2 c**). Calls by demuxlet are shown in columns (singlets: X2, X3, X4; doublets; ambiguous), and true labels from the simulation are shown in rows (singlets: X2, X3, X4; non-identifiable doublets from the same sample: X2-X2, X3-X3, X4-X4; identifiable doublets: dbl-X2-X3, dbl-X2-X4, dbl-X3-X4). Doublets consisting of two cells from the same sample are non-identifiable since these cells contain the same germline SNPs.

	HGSOC, 20% doublets			HGSOC, 30% doublets	
	identified as doublet			identified as doublet	
true doublet	TRUE	FALSE	true doublet	TRUE	FALSE
TRUE	600	**1,003**	TRUE	917	**1,408**
FALSE	**3,720**	6,405	FALSE	**3,214**	4,965

	lung, 20% doublets			lung, 30% doublets	
	identified as doublet			identified as doublet	
true doublet	TRUE	FALSE	true doublet	TRUE	FALSE
TRUE	346	**2,221**	TRUE	326	**3,328**
FALSE	**2,088**	17,678	FALSE	**1,129**	14,798

Supplementary Table S5. Summary of doublets identified by applying a downstream doublet detection tool (scDblFinder) to demultiplexed cells after applying top-performing and computationally efficient demultiplexing tools (cellSNP/Vireo with bulk RNA-seq reference for HGSOC dataset; cellSNP/Vireo with 1000 Genomes 3' UTRs filtered reference for lung adenocarcinoma dataset), for 20% and 30% doublets scenarios. scDblFinder was run using default settings, and clusters representing doublets identified using thresholds of 70 (HGSOC) and 10 (lung adenocarcinoma) differentially expressed genes per cluster based on inspection of elbow plots. False positive and false negative doublet calls are shown in bold font.
