# Supplementary material for: Genetic demultiplexing of pooled single-cell RNA-sequencing samples in cancer facilitates effective experimental design
Source: Gigascience. 2021 Sep 22;10(9):giab062. doi: 10.1093/gigascience/giab062 (PMC8458035; doi:10.1093/gigascience/giab062)
Supplement: giab062_Response_to_Reviewer_Comments_Original_Submission [file giab062_response_to_reviewer_comments_original_submission.pdf]

Author's Response To Reviewer Comments

Close

Response to Reviewers

Reviewer 1:

Reviewer #1: The authors have investigated methods to demultiplex samples from single-cell RNA sequencing data based on SNPs. Pooling of cancer samples is certainly an important tool for cost reduction and minimizing batch effects as the authors have pointed out. It nice to see the well documented, freely available github repository.

Major Points:

The authors have tested demultiplexing based on matched reference data, publicly available SNPs and without any SNP reference. It has to be noted however, that without a matching reference the samples can be demultiplexed but not assigned to a specific donor. This has to be mentioned in the text as this is a crucial feature.

This point has now been mentioned more clearly in the Introduction (paragraph 2) and Discussion (final paragraph).

In the cancer context it would be important to test also reference data from a SNP array in addition to bulk RNA-seq data. It would be interesting to see which matched reference would perform better. Could the authors provide such an analysis for the HGSOC dataset?

This is an important point, which we had not previously considered. Since we did not have access to SNP array data for the HGSOC samples, we designed an additional simulation strategy based on the existing samples, to test the performance when using a SNP array. In brief, we found that using reference data from a SNP array performs remarkably well and we describe the additional analyses and figures that have now been added to the manuscript in response to this excellent suggestion.

We downloaded the set of SNPs from a widely used SNP array (the Infinium Multi-Ethnic Global-8 v1.0 array from the Multi-Ethnic Global Array or MEGA Consortium, which includes 1.7 million SNPs), and then calculated the sets of overlapping SNPs between the SNP array and (i) our bulk RNA-seq genotype reference (605,367 total SNPs before calculating overlap) and (ii) the 1000 Genomes reference filtered to 3’ UTRs (84,853 total SNPs before calculating overlap). Then, we re-ran the benchmark evaluations using these smaller sets of overlapping SNPs. We note that this strategy effectively simulates a worst-case scenario for the SNP array, since we are using only a small subset of the SNPs from the array (stating this another way, we anticipate the following results would improve if we had access to the full 1.7 million SNP array). Compared to the SNP array, the overlapping sets contain 2.6% of SNPs (bulk RNA-seq) or 0.8% of SNPs (1000 Genomes filtered); while compared to our original references the overlapping sets contain 7.6% (bulk RNA-seq) or 16.5% (1000 Genomes filtered) of the original SNPs (see Supplementary Table 1 for a summary of the set sizes).

Despite the extreme reduction in number of SNPs compared to the full array or the original references, demultiplexing performance for cellSNP/Vireo remains remarkably high, especially when using the bulk RNA-seq reference SNPs. However, the performance of demuxlet drops substantially. (See Supplementary Figure 3; Results and Methods). Since we are using only a small subset of the array SNPs, these results give us confidence that good demultiplexing performance can be achieved when using SNP arrays in the cancer context.

The increased robustness of cellSNP/Vireo compared to demuxlet is also consistent with our

observations for the additional supplementary analyses, e.g. adding ambient RNA from simulated debris or lysed cells, as described below in comments for Reviewer 2.

Could the authors run a real demultiplex experiment in addition to mixing reads in silico (e.g. for the HGSOC samples)?

We thank the reviewer for this comment and agree an experimental evaluation (as opposed to an in silico evaluation) would strengthen the results. Unfortunately, it was not possible to generate additional experimental data within the revision timeline for this manuscript. However, we have now included additional analyses in our revised manuscript that significantly strengthen the evidence for our central claim: genetic variation-based demultiplexing works well in the cancer context.

In particular, this claim is supported by the additional results of (i) subset of SNPs from SNP array (see above), (ii) simulated ambient RNA from debris or lysed cells (see comments for Reviewer 2 below), and (iii) confirmation of baseline performance in non-cancer data (comments for Reviewer 2), all of which demonstrate that cellSNP/Vireo maintain performance in settings that we had not previously tested.

Finally, we have now expanded the Discussion to note that additional experimental data could be used to further strengthen these claims, if it becomes available. (We have also mentioned that the main difficulty when working with experimental data will be in determining a reliable ground truth.)

Can the authors comment on the number of doublets identified with their technique and run at least one doublet detection tool in comparison? For HGSOC (n=3) half of all doublet should be mixed doublets and half harbours cells from the same sample.

We have added additional results and discussion to address this point, which was also raised by Reviewer 2. (Previously, this information was only included implicitly within the precision and recall calculations.) In Supplementary Tables 3 and 4, we have included tables comparing the true and false doublet calls by sample for the top-performing set of tools (cellSNP/Vireo with bulk RNA-seq reference) and the comparison with demuxlet (with bulk RNA-seq reference) for the HGSOC dataset with 30% doublets. This shows that the doublet calls from cellSNP/Vireo are relatively pure (average of 99.2% true identifiable doublets among called doublets), while the reduction in overall precision is due to additional true identifiable doublets incorrectly called as singlets. By contrast, demuxlet returns a much lower percentage (31.9%) of true identifiable doublets among the doublet calls.

We also ran a recent doublet detection tool (scDblFinder; https://bioconductor.org/packages/scDblFinder) as an illustration to see if we could use these tools to identify any remaining doublets after demultiplexing. However, this did not perform well, giving large proportions of both false positives and false negatives (Supplementary Table 5). We hypothesize that this may be due to the nature of cancer samples, where clustering on expression profiles returns more varied clusters than in non-cancer data. While there are benchmark evaluations of doublet detection tools in the non-cancer setting (e.g. https://doi.org/10.1016/j.cels.2020.11.008), we feel a comprehensive evaluation of doublet detection tools applied here (e.g. using interactive plots and investigating additional possible reasons for reduced performance in cancer compared to non-cancer data) is outside the scope of this manuscript. Finally, we have extended the Discussion to mention these limitations more clearly, and to suggest directions for future work in this area.

Minor points:

In the discussion the authors state that they compared the best performing tools against alternative tools (page 15, line 344) - however it is essentially cellSNPVireo vs demuxlet (one vs another). Should be clearer in the text.

We have clarified this sentence, and expanded the Introduction and Discussion to make it more clear that we are comparing these two tools, and not performing a comprehensive benchmark for all available tools.

Can the authors comment on hashing and how hashing would perform in comparison to genetic

demultiplexing in the discussion?

In general, we would expect cell hashing to be the gold standard in terms of performance, while the performance of genetic variation-based demultiplexing depends largely on the computational tool used and the quality of the genotype reference. However, genetic variation-based demultiplexing also provides significant advantages in terms of simpler sample preparation and cost savings during library preparation. We have added additional text to expand on this in the Discussion, and have also mentioned cell hashing approaches in the Introduction.

Reviewer 2:

Reviewer #2: This paper addresses an important and untested question of whether single cell genotype demultiplexing works on cancer samples.

1. The authors use in-silico mixtures of individual single cell cancer experiments with varying percentages of doublet cell barcodes. While it is understandable due to the "finite and irreplaceable nature of tumor cells", it potentially reduces the value, accuracy, and applicability to real data especially due to my next issue. To me this is not a deal breaker, but a distinct limitation.

We agree that demonstrating demultiplexing performance on real experimental data would further strengthen our conclusions, compared to relying on simulations. However, while acknowledging this limitation, our expanded set of simulation scenarios provides strong evidence for our central claim that genetic variation-based demultiplexing tools perform well for cancer data.

In particular, as mentioned above for Reviewer 1, our additional supplementary analyses on (i) using a subset of overlapping SNPs from the MEGA SNP array (see comments for Reviewer 1), (ii) ambient RNA from debris or lysed cells, and (iii) confirmation of performance in healthy samples, significantly strengthen our evidence for our conclusions compared to our original manuscript. We thank the reviewers for these suggestions.

We have also ensured that our wording in the Introduction and Discussion makes it clear that our results are based on simulations generated by modifying real data.

2. Ambient RNA from lysed cells prior to partitioning is not addressed in either simulation or discussion. I think that it should be at least discussed as necrosis is not uncommon in cancer samples. It is important to note that as ambient RNA increases, demuxlet quickly begins classifying almost everything as a doublet while other methods (vireo, souporcell) are more robust to this type of noise.

This was an important omission in our previous in silico benchmark evaluation, and we thank the reviewer for this suggestion. We have now added additional scenarios that include ambient RNA from simulated cell debris or lysed cells by computationally assigning all sequencing reads from a certain percentage of cell barcodes (10%, 20%, or 40%) to other randomly selected cell barcodes, and re-running the demultiplexing tools (results included as new Figure 2 and Supplementary Figures 1-2). These results demonstrate that overall demultiplexing performance decreases in terms of recall, although the effect is minimized when using the top-performing set of tools (cellSNP/Vireo with bulk RNA-seq reference). As suggested above, the performance of demuxlet drops much more substantially, suggesting that cellSNP/Vireo is more robust to this type of noise. We have included additional discussion of this issue in Results and Discussion. In particular, this suggests the importance of considering experimental techniques such as straining to reduce cell debris in cancer samples, when possible.

3. A full normal sample is not shown for comparison to cancer samples. This could be taken from free available datasets. For instance the Hipsci datasets from my souporcell paper could be used. You would want to try to normalize for UMI/cell with subsampling and match roughly the number of cells per individual and then of course match the doublet rate. The number and type of cancer samples tested are fairly limited but adequate for an initial evaluation.

We thank the reviewer for this comment. We have now incorporated additional simulations based on the

five individual iPSC cell lines in the souporcell paper (https://doi.org/10.1038/s41592-020-0820-1), as a healthy (non-cancer) baseline comparison, including both 20% and 30% doublet scenarios. Normalizing the UMI counts per sample became too computationally intensive, so we have instead reported additional details (number of cells per sample, detected genes per cell, UMI counts per cell) for all three datasets (HGSOC, lung, cell line) (Supplementary Table 2) to ensure that comprehensive information is provided for readers to compare the datasets. Performance in the cell line data (Supplementary Figure 4) was comparable to the main benchmark scenarios, giving us confidence that our simulation framework can be reliably applied in both cancer and non-cancer settings, and providing a useful additional baseline comparison that is consistent with previous published results in non-cancer data.

3. I think the author's results actually warrant a stronger conclusion in the question of fully supervised and genotype model based methods (demuxlet, which assumes allele fractions must match the stated genotype) vs cluster center based and thus free allele fraction methods (vireo, souporcell etc). This also has a theoretical advantage in cancer samples which will have non-standard allele fractions around copy number alterations and somatic mutations. Vireo clearly outperforms demuxlet in accurate doublet detection (demuxlet's rigid model system results in over-calling doublets which is presumably what is crushing demuxlet's recall in some samples).

We have now provided additional details on performance in terms of types of doublet calls for both cellSNP/Vireo and demuxlet (Supplementary Tables 3 and 4), as well as the additional results for ambient RNA and subsets of SNPs from an array (Figure 2 and Supplementary Figures 1-3). As suggested by the reviewer, cellSNP/Vireo consistently outperforms demuxlet by a wide margin, especially in these more difficult simulation scenarios. We have included additional wording in Results and Discussion to make these comparisons between the two tools more clear for readers.

4. The paper mentions that the authors prefer high recall over precision but does not discuss the potential downsides of low precision.

We have included additional text to clarify this. Specifically, we prefer to retain all true singlet cells (high recall) if possible, since there is the possibility of applying additional downstream analyses (visualizations, doublet detection tools) to identify any remaining doublets (which is one of the possible sources of reduced precision). In addition, we have included illustrative tables of results comparing the types of doublet calls for both cellSNP/Vireo (top-performing scenario with bulk RNA-seq reference, HGSOC, 30% doublets; Supplementary Table 3) and demuxlet (with bulk RNA-seq reference; Supplementary Table 4), as suggested below, which provides additional information on the types of errors. As suggested by Reviewer 1, we also ran a recent doublet detection tool (scDblFinder; https://bioconductor.org/packages/scDblFinder), but this did not perform well, giving large proportions of both false positives and false negatives (Supplementary Table 5), possibly due to the more varied expression in cancer data compared to non-cancer affecting clustering performance. While benchmarks of doublet detection tools exist for non-cancer data (e.g. https://doi.org/10.1016/j.cels.2020.11.008), in our view a comprehensive evaluation of doublet detection tools applied here is outside the scope of this manuscript.

5. Most importantly I think it would also be useful to discuss how low recall and precision could be attained and in outlier samples discuss which error modes were observed. You could attain low recall due to 1. high unassigned cells. 2. overcalling doublets. 3. misassigned cells. And you could attain low precision through 1. misassigned cells 2. undercalling doublets. These different error modes are very different and could affect downstream processing and inference in different ways. I think that it is important to tease out which of these phenomenon is going on with each sample. I grant that it does not lend itself to standard statistical metrics such as precision/recall as there are more than 2 states of truth (single cell correct sample, single cell incorrect sample, doublet cell). A confusion matrix heatmap would be one option but showing this for many samples would be information overload. At the very least a discussion of the outlier samples should have a breakdown of these numbers with a discussion of why this happens (as explained previously). This would be useful for the demuxlet runs on HGSDC explaining low recall and for the relatively low recall on all runs for the HGSDC 30% doublet run.

We thank the reviewer for this suggestion. This information was previously only implicitly contained within the precision and recall calculations, and we agree it is informative to provide an illustration of these error modes for readers. We have included tables of results comparing the types of true and false

singlet and doublet calls for two illustrative scenarios: the top-performing set of tools (cellSNP/Vireo with bulk RNA-seq reference) in HGSOC with 30% doublets (Supplementary Table 3), and the comparison for demuxlet (with bulk RNA-seq reference, HGSOC, 30% doublets) (Supplementary Table 4). This illustrates the differences in the types of doublet calls between the two tools. For cellSNP/Vireo, the doublet calls are relatively pure, with 99.2% of called doublets being true identifiable doublets. By contrast, for demuxlet, this percentage is much lower at 31.9%. These tables also illustrate the distinction between identifiable doublets (two cells from different donors, with different SNP profiles) and non-identifiable doublets (two cells from the same donor, which cannot be distinguished by their SNP profiles). For cellSNP/Vireo, almost all the non-identifiable doublets are assigned to the correct individual sample, while for demuxlet these contain additional false positive doublet calls.

6. One specific comment I have is with regards to the following sentence. "More fundamentally, due to the reliance on genetically distinct SNP profiles, genetic demultiplexing tools are expected to work well for human samples from unrelated individuals, but are not applicable to biological samples from inbred mice or hereditary related human populations, or samples from the same individual [3]" Almost all of this is true, but in our souporcell paper we demultiplexed a maternal/fetal sample so these tools are applicable to related humans. Of course power decreases the more related the individuals are (in the case of inbreeding). Perhaps the wording could be changed.

We have re-worded this sentence to clarify this issue, as suggested.

Close
