# Supplementary material for: Genetic demultiplexing of pooled single-cell RNA-sequencing samples in cancer facilitates effective experimental design
Source: Gigascience. 2021 Sep 22;10(9):giab062. doi: 10.1093/gigascience/giab062 (PMC8458035; doi:10.1093/gigascience/giab062)
Supplement: giab062_Reviewer_2_Report_Original_Submission — Haynes Heaton, MD -- 4/2/2021 Reviewed [file giab062_reviewer_2_report_original_submission.pdf]

Reviewer Report

Title: Genetic demultiplexing of pooled single-cell RNA-sequencing samples in cancer facilitates effective experimental design

Version: Original Submission **Date:** 4/2/2021

Reviewer name: Haynes Heaton, MD

Reviewer Comments to Author:

This paper addresses an important and untested question of whether single cell genotype demultiplexing works on cancer samples.
1. The authors uses in-silico mixtures of individual single cell cancer experiments with varying percentages of doublet cell barcodes. While it is understandable due to the "finite and irreplaceable nature of tumor cells", it potentially reduces the value, accuracy, and applicability to real data especially due to my next issue. To me this is not a deal breaker, but a distinct limitation.
2. Ambient RNA from lysed cells prior to partitioning is not addressed in either simulation or discussion. I think that it should be at least discussed as necrosis is not uncommon in cancer samples. It is important to note that as ambient RNA increases, demuxlet quickly begins classifying almost everything as a doublet while other methods (vireo, souporcell) are more robust to this type of noise.
3. A full normal sample is not shown for comparison to cancer samples. This could be taken from free available datasets. For instance the Hipsci datasets from my souporcell paper could be used. You would want to try to normalize for UMI/cell with subsampling and match roughly the number of cells per individual and then of course match the doublet rate. The number and type of cancer samples tested are fairly limited but adequate for an initial evaluation.
3. I think the author's results actually warrant a stronger conclusion in the question of fully supervised and genotype model based methods (demuxlet, which assumes allele fractions must match the stated genotype) vs cluster center based and thus free allele fraction methods (vireo, souporcell etc). This also has a theoretical advantage in cancer samples which will have non-standard allele fractions around copy number alterations and somatic mutations. Vireo clearly outperforms demuxlet in accurate doublet detection (demuxlet's rigid model system results in over-calling doublets which is presumably what is crushing demuxlet's recall in some samples).
4. The paper mentions that the authors prefer high recall over precision but does not discuss the potential downsides of low precision.
5. Most importantly I think it would also be useful to discuss how low recall and precision could be attained and in outlier samples discuss which error modes were observed. You could attain low recall due to 1. high unassigned cells. 2. overcalling doublets. 3. misassigned cells. And you could attain low precision through 1. misassigned cells 2. undercalling doublets. These different error modes are very different and could affect downstream processing and inference in different ways. I think that it is important to tease out which of these phenomenon is going on with each sample. I grant that it does not lend itself to standard statistical metrics such as precision/recall as there are more than 2 states of truth (single cell correct sample, single cell incorrect sample, doublet cell). A confusion matrix heatmap

would be one option but showing this for many samples would be information overload. At the very least a discussion of the outlier samples should have a breakdown of these numbers with a discussion of why this happens (as explained previously). This would be useful for the demuxlet runs on HGSDC explaining low recall and for the relatively low recall on all runs for the HGSDC 30% doublet run.
6. One specific comment I have is with regards to the following sentence. "More fundamentally, due to the reliance on genetically distinct SNP profiles, genetic demultiplexing tools are expected to work well for human samples from unrelated individuals, but are not applicable to biological samples from inbred mice or hereditary related human populations, or samples from the same individual [3]" Almost all of this is true, but in our souporcell paper we demultiplexed a maternal/fetal sample so these tools are applicable to related humans. Of course power decreases the more related the individuals are (in the case of inbreeding). Perhaps the wording could be changed.

Methods

Are the methods appropriate to the aims of the study, are they well described, and are necessary controls included? Choose an item.

Conclusions

Are the conclusions adequately supported by the data shown? Choose an item.

Reporting Standards

Does the manuscript adhere to the journal's guidelines on [minimum standards of reporting?]() Choose an item.

Choose an item.

Statistics

Are you able to assess all statistics in the manuscript, including the appropriateness of statistical tests used? Choose an item.

Quality of Written English

Please indicate the quality of language in the manuscript: Choose an item.

Declaration of Competing Interests

Please complete a declaration of competing interests, considering the following questions:

- Have you in the past five years received reimbursements, fees, funding, or salary from an organisation that may in any way gain or lose financially from the publication of this manuscript, either now or in the future?

- Do you hold any stocks or shares in an organisation that may in any way gain or lose financially from the publication of this manuscript, either now or in the future?
- Do you hold or are you currently applying for any patents relating to the content of the manuscript?
- Have you received reimbursements, fees, funding, or salary from an organization that holds or has applied for patents relating to the content of the manuscript?
- Do you have any other financial competing interests?
- Do you have any non-financial competing interests in relation to this paper?

If you can answer no to all of the above, write 'I declare that I have no competing interests' below. If your reply is yes to any, please give details below.

1. I received a salary from 10x Genomics most recently in march of 2017 2. I no longer hold stock in txg but did so as recently as march 2020 3. no 4. see 1 5. no 6. no

I agree to the open peer review policy of the journal. I understand that my name will be included on my report to the authors and, if the manuscript is accepted for publication, my named report including any attachments I upload will be posted on the website along with the authors' responses. I agree for my report to be made available under an Open Access Creative Commons CC-BY license (http://creativecommons.org/licenses/by/4.0/). I understand that any comments which I do not wish to be included in my named report can be included as confidential comments to the editors, which will not be published.

Choose an item.

To further support our reviewers, we have joined with Publons, where you can gain additional credit to further highlight your hard work (see: https://publons.com/journal/530/gigascience). On publication of this paper, your review will be automatically added to Publons, you can then choose whether or not to claim your Publons credit. I understand this statement.

Yes Choose an item.
